# Multi-Omic, Histopathologic, and Clinicopathologic Effects of Once-Weekly Oral Rapamycin in a Naturally Occurring Feline Model of Hypertrophic Cardiomyopathy: A Pilot Study

**DOI:** 10.3390/ani13203184

**Published:** 2023-10-12

**Authors:** Victor N. Rivas, Joanna L. Kaplan, Susan A. Kennedy, Stuart Fitzgerald, Amanda E. Crofton, Aisling Farrell, Louise Grubb, Carina E. Jauregui, Gabriela Grigorean, Eunju Choi, Samantha P. Harris, Joshua A. Stern

**Affiliations:** 1 Department of Medicine and Epidemiology, School of Veterinary Medicine, University of California-Davis, Davis, CA 95616, USA; 2 Department of Clinical Sciences, College of Veterinary Medicine, North Carolina State University, Raleigh, NC 27607, USA; 3 TriviumVet, X91 DEC4 Waterford, Irelandstuart.fitzgerald@triviumvet.com (S.F.);; 4 Proteomics Core Facility, University of California-Davis, Davis, CA 95616, USA; 5 Department of Pathology, Microbiology, and Immunology, School of Veterinary Medicine, University of California-Davis, Davis, CA 95616, USA; eachoi@ucdavis.edu; 6 Department of Physiology, College of Medicine-Tucson, University of Arizona, Tucson, AZ 85724, USA

**Keywords:** mTOR (mammalian/mechanistic target of rapamycin), transcriptomics (RNA sequencing), proteomics (LCMS), sirolimus, autophagy, cat, translational, large animal model

## Abstract

**Simple Summary:**

Hypertrophic cardiomyopathy (HCM) is the single most common cardiomyopathy in cats with no available therapeutic options in the asymptomatic period of the disease. The mechanisms by which rapamycin may exert effects at the level of the heart muscle remains elusive. This pilot study investigates the effects of oral intermittent low-dose rapamycin in HCM-affected cats at the level of the heart muscle, plasma, and urine. Treatment with rapamycin remains well tolerated in HCM-affected cats with no adverse events. Anti-inflammatory effects of rapamycin treatment in cardiac tissues were observed. Dose-responsive effects of rapamycin on autophagy were identified. Changes in the abundance of metabolic protein in tissue and plasma were observed. Rapamycin leads to an underabundance of complement and coagulation cascade proteins. Minimal effects on fat and cholesterol metabolism, lipid, neuron projection, and blood microparticle proteins were observed in the plasma and urine samples of cats pre- and post-treatment with rapamycin. Taken altogether, rapamycin treatment continues to show great mechanistic promise in addressing the pathophysiologic features of HCM as well as the possible disease sequalae of thromboembolism.

**Abstract:**

Hypertrophic cardiomyopathy (HCM) remains the single most common cardiomyopathy in cats, with a staggering prevalence as high as 15%. To date, little to no direct therapeutical intervention for HCM exists for veterinary patients. A previous study aimed to evaluate the effects of delayed-release (DR) rapamycin dosing in a client-owned population of subclinical, non-obstructive, HCM-affected cats and reported that the drug was well tolerated and resulted in beneficial LV remodeling. However, the precise effects of rapamycin in the hypertrophied myocardium remain unknown. Using a feline research colony with naturally occurring hereditary HCM (n = 9), we embarked on the first-ever pilot study to examine the tissue-, urine-, and plasma-level proteomic and tissue-level transcriptomic effects of an intermittent low dose (0.15 mg/kg) and high dose (0.30 mg/kg) of DR oral rapamycin once weekly. Rapamycin remained safe and well tolerated in cats receiving both doses for eight weeks. Following repeated weekly dosing, transcriptomic differences between the low- and high-dose groups support dose-responsive suppressive effects on myocardial hypertrophy and stimulatory effects on autophagy. Differences in the myocardial proteome between treated and control cats suggest potential anti-coagulant/-thrombotic, cellular remodeling, and metabolic effects of the drug. The results of this study closely recapitulate what is observed in the human literature, and the use of rapamycin in the clinical setting as the first therapeutic agent with disease-modifying effects on HCM remains promising. The results of this study establish the need for future validation efforts that investigate the fine-scale relationship between rapamycin treatment and the most compelling gene expression and protein abundance differences reported here.

## 1. Introduction

The mammalian/mechanistic target of rapamycin (mTOR) serine/threonine protein kinase is a master regulator of eukaryotic cell growth, metabolism, and promotion of protein synthesis [1,2]. In mammals, mTOR functions in two distinct multi-subunit complexes, mTOR complex 1 (mTORC1) and mTOR complex 2 (mTORC2) [1,3,4]. Rapamycin (sirolimus) is a specific inhibitor of the mTORC1 protein complex, which acts as a central regulator of cell growth and nutrient response [2]. It has been used extensively in clinical medicine in humans, principally to prevent the rejection of organ transplants. Experimental evidence suggests that mTORC1 inhibition is associated with several beneficial cardiovascular effects, including reducing cardiac hypertrophy and improving cardiac function in the presence of pressure overload and genetically induced cardiomyopathies, reducing ischemic injury after acute and chronic myocardial infarction, and reactivating cardiac autophagy [5,6,7,8,9,10]. Published research suggests that these effects are likely dependent on the fact that partial inhibition of mTORC1 abolishes the detrimental effects of mTORC1’s maladaptive functions during cardiac stress, while still maintaining its physiological functions [2,11]. Treatment with rapamycin has been shown to improve markers of cardiac health and function in dogs, rodents, and humans [5,6,10,12,13,14,15]. However, chronic or high-dose rapamycin therapy leads indirectly to down-regulation of mTORC2; the precise mechanisms by which this occurs are unclear, as rapamycin does not interact directly with this complex. Two hypotheses prevail: (1) mTOR depletion and (2) the existence of an as-yet undefined downstream and indirect counterregulatory signaling mechanism [16,17,18,19]. Due to a lack of specific mTORC2 inhibitors and the fact that disruption of mTORC2 due to chronic rapamycin treatment is cell-type and context specific, research to date has focused on mTORC1 inhibition.

Hypertrophic cardiomyopathy (HCM) remains the most common cardiomyopathy in cats, with a staggering prevalence of ~15% in the general population [20,21,22]. The disease is largely considered genetic, commonly caused by mutations with pathogenic effects resulting in cardiac sarcomere hypercontractility [23]. The disease is characterized by concentric hypertrophy of the heart’s left ventricular (LV) posterior wall or interventricular septum (IVS) in the absence of pressure overload or other metabolic disorders, leading to reduced LV lumen volume. To date, direct treatment options for mitigating cardiac hypertrophy do not exist, however, limited therapies for treatment of the most sinister disease outcomes (e.g., left-sided congestive heart failure [CHF], thromboembolic events, and sudden cardiac death) are available in veterinary and human patients. Mitigation of left ventricular outflow tract obstruction in humans is achieved through the recent advent of myosin-inhibiting compounds [24,25,26,27]. Collectively, these data emphasize the need for a pharmaceutical compound to treat the primary feature of HCM (maladaptive LV hypertrophy) in veterinary and human patients alike.

Significant evidence suggests autophagy is inhibited in aortic-banded models of LV hypertrophy, human septal myectomy samples, and mice with HCM-causing genetic mutations [5,8,28]. mTORC1 is activated during pathologic hypertrophy induced by transverse aortic constriction and spontaneous hypertension and may be later inactivated when cardiac function deteriorates and heart failure develops [2]. Inhibition of mTORC1 results in the inhibition of hypertrophy [5,6,10]. Alterations in both autophagy and mitophagy are involved in the pathogenesis of several cardiomyopathies [29]. Rapamycin treatment and caloric restriction have been shown to have positive effects in different models of pressure overload-induced and age-related hypertrophy. Treatment with rapamycin or caloric restriction to inhibit mTORC1, and thereby activate autophagy, partially rescued heart failure and the HCM phenotype and restored autophagic flux in knock-in mice [8]. These data agree with previous findings showing that rapamycin administration to mice harboring pathogenic HCM genetic mutations resulted in improvements in cardiac structure and function [9,10]. We previously embarked on a study aimed to evaluate echocardiographic, biochemical, and cardiac biomarker effects of chronic (six-month) delayed-release (DR) rapamycin dosing in a client-owned population of subclinical, non-obstructive, HCM-affected cats [30]. We observed that a 0.30 mg/kg dose resulted in LV remodeling, supported by a decrease in LV maximal wall thickness (MWT) when compared to a placebo group (*p* < 0.01) [30]. Treatment of rapamycin was well tolerated by all cats with no significant differences in adverse events between groups. Though rapamycin is currently a promising compound for veterinary use in cats, the precise mechanisms of action of the drug in the hypertrophied myocardium remain unknown. Using a feline research colony with naturally occurring hereditary HCM (n = 9), the aim of this study was to examine the tissue-, urine-, and plasma-level proteomic and tissue-level transcriptomic effects of an intermittent low dose (0.15 mg/kg) and high dose (0.30 mg/kg) of DR oral veterinary rapamycin once weekly. The chosen dose rates were supported by previous work in cats with HCM, a toxicokinetic study in healthy cats, and a study in dogs supporting cardiac benefits at a comparable dose [12,30]. Although this study was originally designed as an exploratory pilot, we hypothesized that rapamycin treatment would lead to dose-response effects in genes/peptides involved in mTOR-associated processes such as autophagy, peptide synthesis, and mitochondrial biogenesis in cats with subclinical HCM.

## 2. Methods

### 2.1. Ethics Statement

All procedures were approved by the Institutional Animal Care and Use Committee of the University of California-Davis (Davis, CA, USA) and carried out in accordance with the ARRIVE guidelines (https://arriveguidelines.org/; accessed on 9 August 2023) and regulations (protocol #22376).

### 2.2. Animals and Study Design

**Figure 1:**
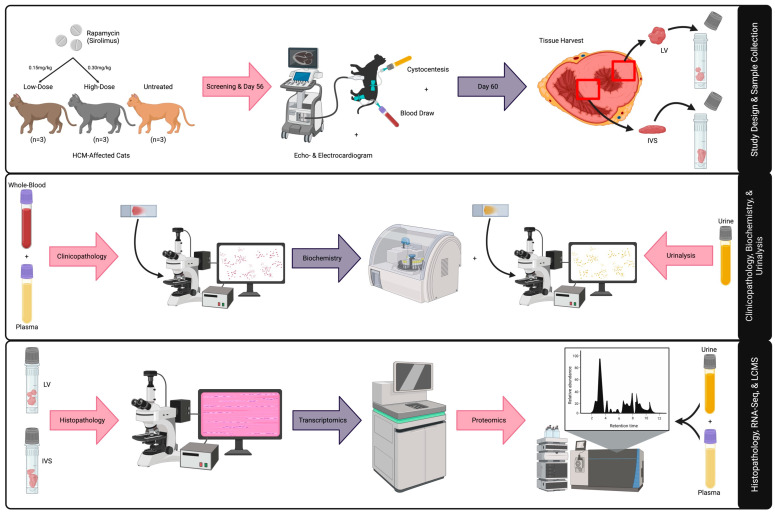
Graphic summary of study design and methods. An illustrative representation of study design and multi-omic techniques for LV, IVS, blood, and urine specimens is provided. Abbreviations: HCM = hypertrophic cardiomyopathy, LV = left ventricle, IVS = interventricular septum, LCMS = liquid chromatography mass spectrometry.

This is a blinded, randomized, preclinical trial assessing the effects of intermittent dosing of a novel DR rapamycin product (https://patents.google.com/patent/WO2021124264A1/en; accessed on 9 August 2023) in an intact purpose-bred research colony cats with non-obstructive HCM [31]. A total of nine HCM-affected cats, previously confirmed by a board-certified veterinary cardiologist (J.L.K.), were included in this study (Supplemental Table S1). Six of the nine cats were equally randomized to either low-dose (0.15 mg/kg; average age [years] = 2.2) or high-dose (0.30 mg/kg; average age [years] = 1.8) DR rapamycin; cats were given the study drug once every seven days (±two) for a 56-day period. The tissues from three previously collected and archived HCM-positive control cats were used in this study (average age [years] = 10.3). All members of the study team, except for the treatment dispenser (C.E.J.), were blinded to treatment groups throughout the study. Cats with non-obstructive HCM confirmed by an echocardiographic diagnosis (2D or M-mode LVd > 6 mm, excluding insertion sites of moderator bands), that were >1 and <10 years of age, with no evidence of CHF, and that were otherwise healthy were included in the study. The primary data and sample collection study days of the in-life phase occurred upon screening (no more than seven days prior to Day 0/Dose 1) and on Day 56. Sampling on these days included blood and urine collection for proteomic analysis. Additional data collected on these days include physical exam, systolic blood pressure, echocardiogram, electrocardiogram (ECG), comprehensive biochemistry panel, complete blood cell count (CBC), N-terminal prohormone of brain natriuretic peptide (NTproBNP), and urinalysis data. Daily observations were carried out from Day 0 to Day 56 and recorded on daily observation sheets for adverse event monitoring. Removal from the study was planned for any one of the following reasons: adverse event observation that was deemed necessary by the investigator team, necessity for further medical therapy, or observed protocol violations (Figure 1).

### 2.3. Sample Collection

Upon screening (pre-) and on Day 56 (post-treatment), EDTA and whole blood and urine samples were collected via venipuncture and cystocentesis, respectively, for CBC, biochemistry, and urinalysis (IDEXX, Westbrook, ME, USA) (Supplemental Figure S1). Immediately after, additional EDTA blood plasma samples were phosphorylation- and protease-inhibited with 1X PhoSTOP and EDTA-free complete Mini Inhibitor Cocktail (Roche, Basel, Switzerland); urine samples were protease inhibited via room temperature centrifugation at 1000× *g* for 15 min for proteomics. Under the influence of intramuscular (IM) sedation (1–2 mg/kg alfaxalone and 0.30 mg/kg midazolam), humane euthanasia with an overdose of intravenous (IV) pentobarbital (≥100 mg/kg) was performed at Day 60 for tissue collection; LV and IVS tissues were collected no more than 30 min post-euthanasia and immediately flash-frozen upon harvest and stored at −80 °C for subsequent liquid chromatography mass spectrometry (LCMS) and RNA sequencing (RNA-seq) analyses. Convenience samples of LV and IVS tissues from three previously euthanized research colony cats were used as treatment controls for the assessment of tissue-level treatment effects within -omic comparisons. A detailed schedule of study events is provided in Supplemental Table S2.

### 2.4. Cardiologic Evaluations

Oral gabapentin (100 mg) was administered approximately one-hour prior to screening examinations. A complete echocardiogram was conducted upon screening and on Day 56. Routine 2D, M-mode, color, and spectral Doppler echocardiography were performed under the influence of IM sedation (1–2 mg/kg alfaxalone and 0.30 mg/kg midazolam) for ease of patient handling. Measurements of chamber size and wall thickness were obtained by both 2D and M-mode measurements from the right parasternal imaging window. Measurements were recorded as an average of three consecutive cardiac cycles when possible, avoiding any cycles during or immediately following cardiac arrhythmias. Measurement methodology was carried out in accordance with the ACVIM consensus statement guidelines for the classification, diagnosis, and management of cardiomyopathies in cats using leading edge-to-leading edge, leading edge-to-trailing edge, and leading edge-to-leading edge methodologies for M-mode, 2D septal, and 2D LV posterior wall measures of wall thickness, respectively [21].

A three-minute, six-lead, continuous ECG was conducted upon screening and on Day 56. Observed cardiac arrhythmias, if any, were recorded. ECGs were obtained in awake or sedated cats in right lateral recumbency using a six-lead standard frontal plain system. Electrocardiograms were saved at 50 mm/s and 20 mm/mV calibration. All echo- and electrocardiographic assessments were performed by a board-certified veterinary cardiologist (J.L.K.). Illustrative interpretations of the study’s select echocardiographic variables are presented in Supplemental Figure S2.

### 2.5. Histopathology

Cat heart tissues were appropriately fixed in 10% neutral buffered formalin for 24–48 h. Briefly, formalin-fixed tissue samples were routinely processed to produce 4 µm hematoxylin and eosin-stained and Masson’s trichrome-stained slides according to service specifications (VMTH Histopathology Laboratory, Anatomic Pathology Service, UC Davis, Davis, CA, USA). These slides were examined with a light microscope by a board-certified veterinary pathologist (E.C.).

Heart slides were examined and scored (mild, moderate, and severe) for LV endocardial fibrosis, myocardial disarray, and interstitial fibrosis as previously described [32]. Cellular hypertrophy was scored with modifications. LV endocardial fibrosis was considered mild, moderate, and severe when 30–75 µm, 76–150 µm, and >150 µm segments were observed, respectively. Myocardial disarray was considered mild, moderate, and severe when affecting <25%, 26–50%, and >50% of the myocardial area, respectively. Cellular hypertrophy was considered mild when cardiomyocyte thickness averaged 3–4 red blood cells (RBCs) (18–24 µm), moderate when it averaged 4–5 RBCs (25–30 µm), and severe when it averaged 5+ RBCs (>30 µm). Interstitial fibrosis was considered absent when affecting <5% of the section, mild when affecting 6–15% of the section, moderate when affecting 16–25% of the section, and severe when affecting >25% of the section. Coronal vessel remodeling was considered mild when segmental thickening was observed in a few arteries, moderate when segmental to diffuse thickening was observed in a few arteries, and severe when diffuse thickening was frequently observed in arteries. Coronary vessel narrowing was noted as present or absent.

### 2.6. RNA Sequencing

To investigate tissue-level effects of DR rapamycin administration, RNA-seq was employed to determine differentially expressed genes (DEGs) associated with low- and high-dose rapamycin administration compared to control sex- and weight-matched HCM phenotype-positive cats. Tissues of the LV posterior wall and IVS were collected for RNA extraction. Mature RNA transcripts were selected using the polyA selection method. High-quality mature RNA (RNA integrity number [RIN] = 7–10) was converted to cDNA and NEBNext Ultra II stranded library preparations were made. Samples were later used for paired-end 150 bp RNA-seq on the Illumina HiSeq platform with a targeted read depth of 50 million reads per sample (Azenta Life Sciences, South Plainfield, NJ, USA). Sequenced reads underwent quality control to remove Illumina adapter sequences, low quality reads, and duplicated reads with Trimmomatic software (v.0.36). High quality reads were mapped to the annotated ENSEMBL *Felis catus* reference genome (felCat_9.0) using STAR aligner software (v.2.5.2b), where BAM files were generated. Unique gene hit counts were calculated using featureCounts from the Subread package (v.1.5.2) and later used for downstream differential expression analysis. Gene expression values were normalized to adjust for variations in sequencing depth and to accurately determine DEGs. Sample distances from each tissue type were plotted using each sample’s corresponding expression values, and similarities within and between groups were assessed by performing a principal component analysis as a quality check. DESeq2 was used to compare gene expression between low- and high-dose rapamycin and treatment and control HCM cats. Wald’s statistical testing was used to generate *p*-values and log2FoldChanges. Genes with an adjusted *p*-value < 0.05 and a log2FoldChange > 1 or <−1 were labeled as DEGs for each comparison.

### 2.7. Liquid Chromatography Mass Spectrometry

LV and IVS tissues were cut into 0.30 cm pieces and placed in 1 mL of solubilization buffer (5% SDS, 50 mM triethyl ammonium bicarbonate, complete protease inhibitor cocktail [Roche], pH 7.5) inside MagNA Lyser tubes containing ceramic beads (Roche) and were later homogenized with a MagNA Lyser instrument (Roche) to achieve full mechanical disruption. Tissue homogenates, plasma, and precipitated urine protein samples were subjected to proteolysis by using suspension-trap (ProtiFi) devices. All operations of reduction, alkylation, tryptic digestion, and peptide clean-up were carried out within these devices. The eluted tryptic peptides were dried in a vacuum centrifuge and re-constituted in 0.1% trifluoroacetic acid. Peptides were injected into an LCMS system, where they were first trapped on a Thermo PepMap trap for desalting and separated on PepSep 100 μm × 25 cm analytical columns. Chromatography was performed with a Dionex Ultimate 3000 nUPLC at 600 nL/min. Peptides were eluted directly into a Tribrid Lumos Mass Spectrometer (ThermoFisher, Waltham, MA, USA) using a gradient mix of 0.1% formic acid in water and 0.1% formic acid in acetonitrile over a 60 min period. The samples were run in data-independent analysis (DIA) mode; mass spectra were acquired using a collision energy of 35, M1 resolution of 120 k, and MS2 of 30 k, using overlapped isolation windows of 47 Da in a 360–1080 *m*/*z* range and 3 s cycle time.

DIA data was analyzed using Spectronaut 16 (Biognosys AG, Schlieren, Switzerland) and the directDIA workflow with default settings. Briefly, trypsin/P specificity was set for the enzyme, allowing two missed cleavages. Fixed modifications were set for carbamidomethyl, and variable modifications were set to acetyl (protein N-term) and oxidation. For DIA search identification, the peptide-spectrum match (PSM) and protein group false discovery rate (FDR) were set at 0.01%. A minimum of two peptides per protein group was required for quantification. Differential expression was calculated in Spectronaut 16 using its default settings with a combined MS1 precursor and MS2 transition data [33]. Quantitation was normalized to local total ion chromatograms. Statistical comparison of relative protein changes was performed with paired *t*-tests. Finally, proteins identified with less than one unique peptide were excluded from the assay. The significance level was set to a *p*-value_adjusted_ < 0.05 (*Q*-value) and log2 ratio more than 0.58. Both DIA analysis results were filtered within Spectronaut by a 1% FDR on the peptide and protein levels using a target-decoy approach, corresponding to a *Q*-value of <0.01. Differentially expressed peptides (DAPs) with a *Q*-value < 0.05 were considered statistically significant.

### 2.8. Gene Ontology Term and KEGG Pathway Analyses

Up- and downregulated genes were split from the total statistically significant DEGs between the treatment and HCM groups; from the identified list of statistically significant protein abundances, over- and underabundant protein-specific peptides were split. DEGs and DAPs were used for biological process gene ontology (GO) term and KEGG pathway analyses with the use of the ShinyGO tool. To achieve the most reliable results, a total of 15,813 ENSEMBL cat genes and 2813 LV, 2839 IVS, 326 plasma, and 1077 urine cat proteins that were interrogated for RNA-seq and LCMS were set as background. Because the cat genome annotation was not available for ShinyGO enrichment analyses at the time of this study, the GO functional categorizations, gene ID mappings, and quantitative gene characteristics for the human ENSEMBL species were employed to recognize the greatest number of cat gene names possible. Redundancy removal, a pathway size minimum of two, a pathway size maximum of 2000, and an FDR cut-off of 0.05 were set for all term analyses. For proteomic GO and KEGG analyses, in the case that a given protein had >1 statistically significant peptides, only one representative DAP was included in the analyses to minimize false enrichment.

### 2.9. Physical Exam and Clinico- and Histopathology Statistical Analyses

Given the sample size of this study, all data were considered non-normally distributed. Descriptive statistics for demographic and physical exam data are presented as medians (min–max). Comparisons between pre- and post-treatment timepoints for biochemistry, CBC, and urinalysis were performed using a Wilcoxon matched-pairs signed rank test. Statistically significant histopathological differences between the HCM control, low-, and high-dose groups were interrogated via a Kruskal–Wallis test followed by Dunn’s multiple comparison testing. A *p*-value < 0.05 was considered statistically significant for all analyses; sample statistics were not performed for any of the HCM control cats as no paired data was obtained.

## 3. Results

### 3.1. Cardiac, Physical, Clinico-, and Histopathologic Evaluations

No adverse events were reported throughout the study. All screening and Day 56 physical exams were unremarkable. A statistically significant increase in Day 56 body weight (BW) from screening was noted in only the pooled comparison (*p* < 0.03); no other physical exam variables were statistically significant within individual and/or pooled treatment groups (Supplemental Table S3 and Figure S3). No presence of bacteria, casts, glucose, or ketones were observed in urinalysis at any timepoint; no statistically significant pertinent urinalysis variables were identified in any comparison (Supplemental Table S4). Biochemistry analyses were carried out for the following variables: alkaline phosphatase, aspartate transaminase, alanine transaminase, creatine kinase, gamma-glutamyltransferase, albumin, total bilirubin, total protein, globulin, conjugated bilirubin, blood urea nitrogen, creatinine, cholesterol, glucose, calcium, phosphorus, bicarbonate/total CO_2_, chloride, potassium, albumin:globulin ratio, sodium, blood urea nitrogen:creatinine ratio, unconjugated bilirubin, sodium:potassium ratio, anion gap, fructosamine, symmetric dimethylarginine, hemolysis index, lipemia index, and total thyroxine (T4); none of the obtained blood samples were hemolyzed or lipemic. Of all biochemistry measures, only bicarbonate/total CO_2_ was statistically significant between the screening and Day 56, when treatment groups were pooled (*p* < 0.03) (Supplemental Table S5 and Figure S3); however, the values at Day 56 remained well within the reference range and thus are deemed clinically irrelevant. No statistically significant differences between hematologic measures and histopathologic scores were observed between individual and pooled treatment groups (Supplemental Table S6 and Supplemental Table S7, respectively); representative hematoxylin and eosin (H&E) and Masson’s trichrome-stained histopathologic images from the LV posterior wall are provided for all nine study subjects in Supplemental Figure S4 and Supplemental Figure S5, respectively. Electrocardiographic abnormalities were not observed in any of the cats of this study. No statistically significant changes between treated and non-treated cats were observed in any echocardiographic measures (Supplemental Figure S2 and Table S8).

### 3.2. RNA Sequencing

A total of 172 LV (62 down- and 110 upregulated) and 19 IVS (nine down- and 10 upregulated) global DEGs were identified in the tissues of low-dose DR rapamycin-treated cats when compared to controls. Compared to the HCM control cats, 74 LV (38 down- and 36 upregulated) and 49 IVS (20 down- and 29 upregulated) total DEGs were identified in the high-dose treatment group. A total of seven down- and 10 upregulated LV genes were shared between the treatment groups, whereas only one down- and two upregulated genes were shared in the IVS tissues of low- and high-dose groups. When treatment groups were pooled together in the comparison (all doses vs. HCM), 52 (19 down- and 33 upregulated) and 15 (three down- and 12 upregulated) LV and IVS DEGs were identified, respectively. Lastly, to determine dose-dependent effects on the transcriptomic profiling of treated groups, LV and IVS comparisons between low- and high-dose DR rapamycin-treated cats were performed; a total of 49 LV (12 down- and 37 upregulated) DEGs were found, and only one upregulated IVS DEG was observed in the low-dose group when compared to the high-dose. A summary detailing RNA-seq results can be found in Table 1.

We sought to determine down- and upregulated DEGs shared between LV and IVS tissues (Supplemental Table S9). In the low-dose vs. HCM comparison, three down- (*ECRG4*, *P2RX1*, and *TAAR1*) and four upregulated (*DCLK1*, *KERA*, *MYL1*, and *VTN*) genes were shared across tissue types. Four down- (*AS3MT*, *FAM177B*, *HSD17B8*, and *PPDPFL*) and five upregulated (*CNGA1*, *FREM1*, *GRAP2*, *ITGB8*, and *KIT*) DEGs were shared between LV and IVS in the high-dose vs. HCM comparison. Three (*FBLN5*, *FREM1*, and *ITGB8*) and one (*DIPK1A*) shared upregulated DEGs were observed between tissues within the pooled (all doses vs. HCM) and dose-dependent (low- vs. high-dose) comparisons, respectively; no shared downregulated DEGs were present in either of these groups.

### 3.3. Liquid Chromatography Mass Spectrometry

#### 3.3.1. Cardiac Tissues

When compared to HCM control cats, a total of 399 LV (106 under- and 293 overabundant) and 382 IVS (78 under- and 304 overabundant) DAPs were identified in cats receiving low-dose DR rapamycin. In the high-dose treatment group, 449 LV (86 under- and 363 overabundant) and 238 IVS (27 under- and 211 overabundant) DAPs were found. A total of 37 under- and 193 overabundant LV DAPs were shared between treatment groups, whereas 14 under- and 113 overabundant IVS peptides were shared between low- and high-dose groups. In the pooled dose group (all doses vs. HCM), 531 LV (99 under- and 432 overabundant) and 505 IVS (45 under- and 460 overabundant) DAPs were identified. Similar to RNA-seq, to determine dose-dependent effects on the proteomic profiling of treated groups, comparisons between low- vs. high-dose groups revealed two LV (zero under- and two overabundant) and four IVS (one under- and three overabundant) DAPs. A summary detailing proteomics results can be found in Supplemental Table S10.

We also assessed under- and overabundant DAPs shared between LV and IVS tissues (Table 2). In the low-dose vs. HCM comparison, 149 (38 under- and 111 overabundant) DAPs were shared between tissues. A total of 112 (13 under- and 99 overabundant) DAPs were shared between the high-dose vs. HCM comparisons. When all doses were pooled together in the same comparison (all doses vs. HCM) 235 (29 under- and 206 overabundant) DAPs were found to be shared between tissue types. Lastly, no DAP was shared between tissues in the low- vs. high-dose comparison.

#### 3.3.2. Plasma and Urine

DAPs were not identified in either the low- or high-dose group plasma samples after rapamycin treatment; however, when treatment groups were pooled (all doses pre- vs. post-treatment), seven underabundant (ACE2, CXC, IGFBP7, LOC101080826, THBS1, TIMP2, and VWF) and four overabundant (APOA1, APOA4, APOC4, and VNN1) plasma DAPs were found. After treatment administration, 31 (26 under- and five overabundant) DAPs were identified in the low-dose group, although no statistically significant peptides were discovered in the high-dose group. A total of 89 (50 under- and 39 overabundant) urine DAPs were identified after rapamycin treatment when low and high doses were pooled (all doses pre- vs. post-treatment). No DAPs shared between low- and high-dose groups were identified in either plasma or urine samples. A complete list of plasma and urine protein names representative of each DAP are listed in Supplemental Table S11.

### 3.4. Gene Ontology Term and KEGG Pathway Analyses

Due to the extensive results provided in the current study and the breadth of data generated, special attention to the results of LV transcriptomics and proteomics and plasma and urine proteomics has been given in the primary text of this report. The authors have provided complete illustrative results of IVS transcriptomics and proteomics in the Supplementary Files for further information (Supplemental Figures S6–S11).

#### 3.4.1. Differentially Expressed Gene Enrichment of the Left Ventricle

Of the total 48 possible enrichment analyses for all transcriptomic comparisons, seven GO cellular components (CC), 10 GO biological processes (BP), and six KEGG enrichments for DEGs were identified (Supplemental Table S12).

**Figure 2:**
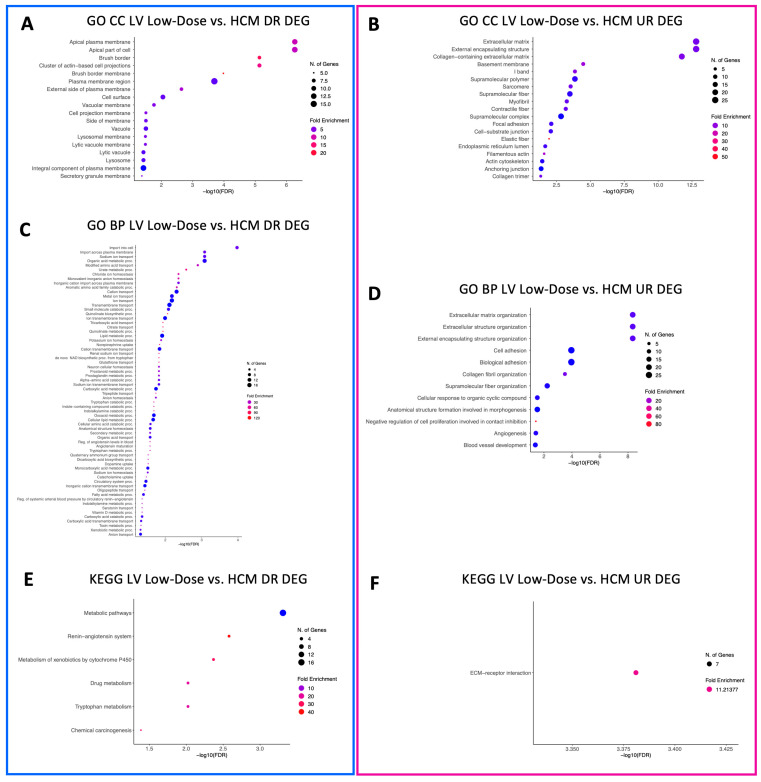
Enriched terms for LV DEGs in the low-dose vs. HCM comparison. Enrichment plots for GO CC (**A**,**B**), GO BP (**C**,**D**), and KEGG pathway (**E**,**F**) term analyses are presented for down- (blue box) and upregulated (pink box) DEGs. The *y*-axis depicts identified enriched terms, whereas the *x*-axis depicts the terms’ significance (−log10[FDR]). The terms are sorted according to statistical significance; the top-most terms on the *y*-axis constitute the most statistically significant terms. The size of individual points depicts the total number of genes binned to a given enriched term; high or low fold enrichment is represented by red or blue coloring, respectively. Abbreviations: HCM = hypertrophic cardiomyopathy, LV = left ventricle, GO = gene ontology, CC = cellular components, BP = biological processes, UR = upregulated, DR = downregulated, DEG = differentially expressed gene(s).

**Figure 3:**
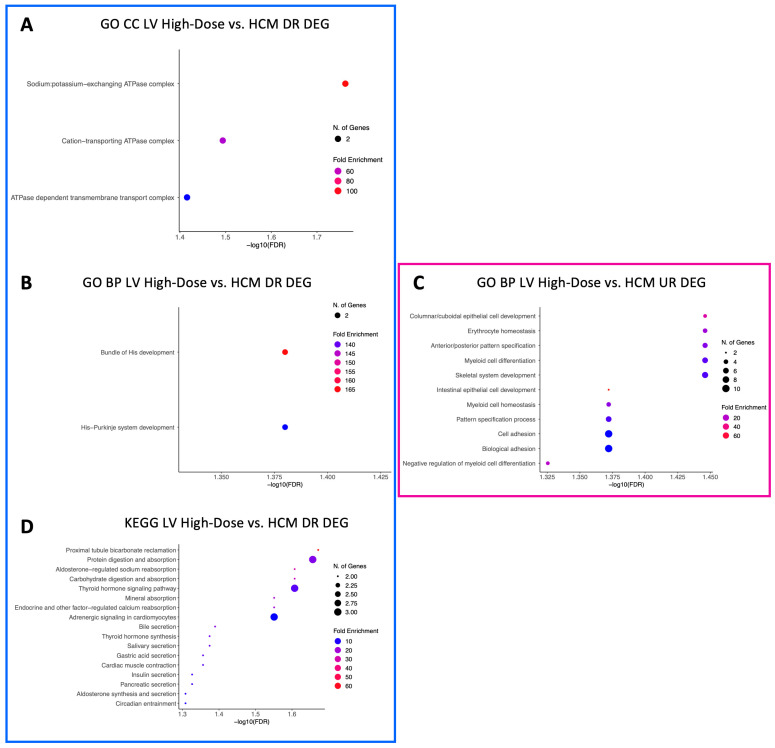
Enriched terms for LV DEGs in the high-dose vs. HCM comparison. Enrichment plots for GO CC (**A**), GO BP (**B**,**C**), and KEGG pathway (**D**) term analyses are presented for down- (blue box) and upregulated (pink box) DEGs. The *y*-axis depicts identified enriched terms, whereas the *x*-axis depicts the terms’ significance (−log10[FDR]). The terms are sorted according to statistical significance; the top-most terms on the *y*-axis constitute the most statistically significant of terms. The size of individual points depicts the total number of genes binned to a given enriched term; high or low fold enrichment is represented by red or blue coloring, respectively. Abbreviations: HCM = hypertrophic cardiomyopathy, LV = left ventricle, GO = gene ontology, CC = cellular components, BP = biological processes, UR = upregulated, DR = downregulated, DEG = differentially expressed gene(s).

**Figure 4:**
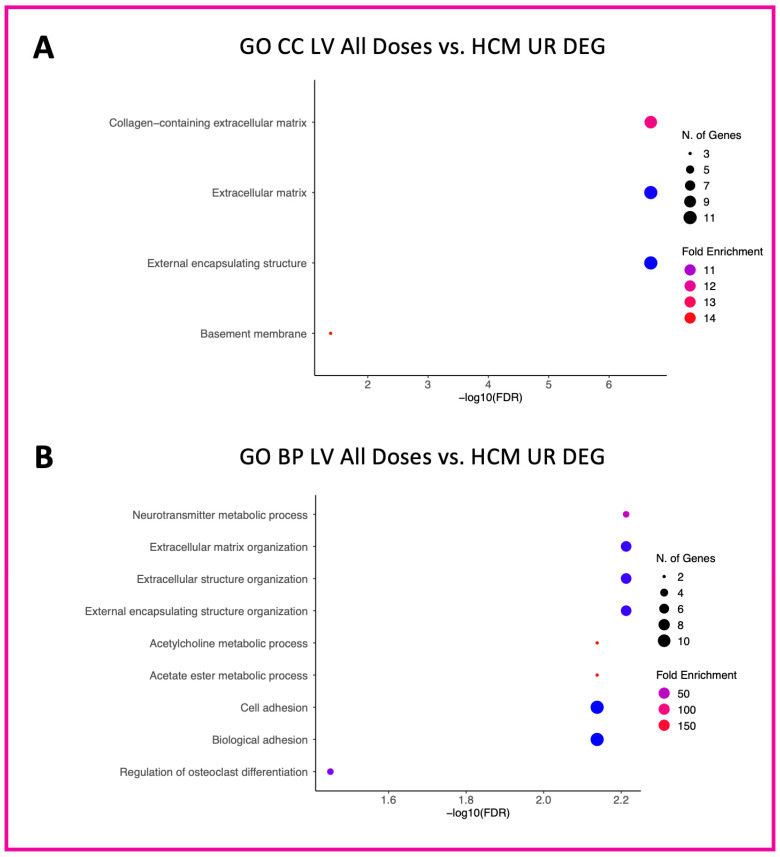
Enriched terms for upregulated LV DEGs in the pooled doses vs. HCM comparison. Enrichment plots for GO CC (**A**) and GO BP (**B**) term analyses are presented for upregulated (pink box) DEGs. The *y*-axis depicts identified enriched terms, whereas the *x*-axis depicts the terms’ significance (−log10[FDR]). The terms are sorted according to statistical significance; the top-most terms on the *y*-axis constitute the most statistically significant of terms. The size of individual points depicts the total number of genes binned to a given enriched term; high or low fold enrichment is represented by red or blue coloring, respectively. Abbreviations: HCM = hypertrophic cardiomyopathy, LV = left ventricle, GO = gene ontology, CC = cellular components, BP = biological processes, UR = upregulated, DEG = differentially expressed gene(s).

**Figure 5:**
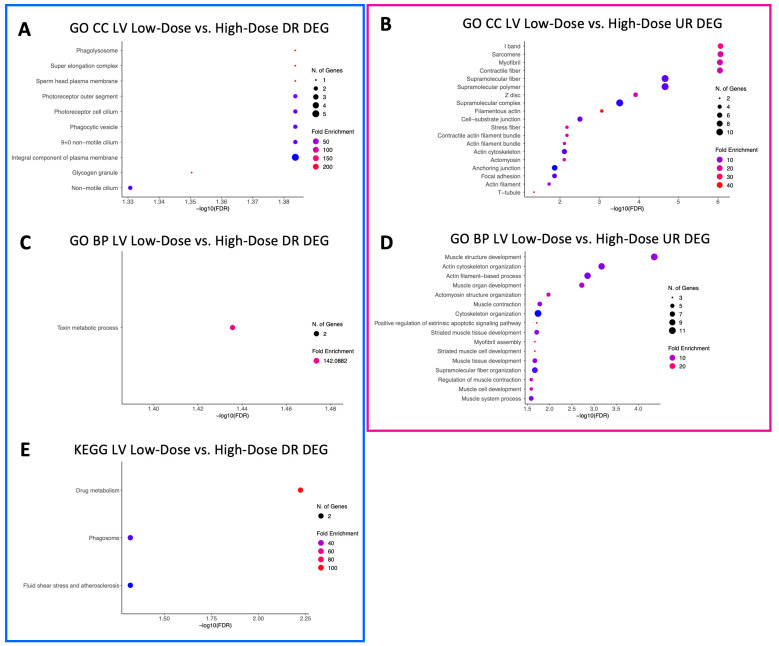
Enriched terms for LV DEGs in the low- vs. high-dose comparison. Enrichment plots for GO CC (**A**,**B**), GO BP (**C**,**D**), and KEGG pathway (**E**) term analyses are presented for down- (blue box) and upregulated (pink box) DEGs. The *y*-axis depicts identified enriched terms, whereas the *x*-axis depicts the terms’ significance (−log10[FDR]). The terms are sorted according to statistical significance; the top-most terms on the *y*-axis constitute the most statistically significant of terms. The size of individual points depicts the total number of genes binned to a given enriched term; high or low fold enrichment is represented by red or blue coloring, respectively. Abbreviations: HCM = hypertrophic cardiomyopathy, LV = left ventricle, GO = gene ontology, CC = cellular components, BP = biological processes, UR = upregulated, DR = downregulated, DEG = differentially expressed gene(s).

A total of 18 GO CC terms were identified for downregulated LV genes in the low-dose vs. HCM comparison, mostly from brush border, projections, vacuole, lysosomal, and plasma membrane commonalities (Figure 2A). Only three GO CC terms (sodium:potassium-exchanging ATPase complex, cation-transporting ATPase complex, and ATPase-dependent transmembrane transport complex) were identified within downregulated DEGs in the high-dose vs. HCM comparison (Figure 3A). Nineteen GO CC terms were enriched in the low-dose vs. HCM comparison for upregulated DEGs; these were congruent with terms relative to the fibers, membrane, extracellular matrix, sarcomere, adhesion, cell junction, and cytoskeleton (Figure 2B). GO CC enrichment for upregulated DEG was not found for the high-dose vs. HCM group; however, when treatment groups were pooled (all doses vs. HCM), four enriched terms were discovered for upregulated DEGs (Figure 4A): basement membrane, collagen-containing extracellular matrix, extracellular matrix, and external encapsulation structures. Rapamycin treatment dose-dependent effects were identified for downregulated DEGs for the low- vs. high-dose comparison; ten enriched terms were identified, including phagolysosome, super elongation complex, sperm head plasma membrane, glycogen granule, photoreceptor outer segment, photoreceptor and non-motile cell cilium (two), and integral component of plasma membrane (Figure 5A). A total of 19 GO CC terms for upregulated DEGs in the low- vs. high-dose comparison was discovered (mostly pertaining to terms of filament, fiber, myofibril, cell-substrate, cytoskeleton, junctions, and supramolecular complex) (Figure 5B).

A total of 66 enriched terms for downregulated DEGs in the low-dose vs. HCM comparison, while only two (bundle of His development and His–Purkinje system development) in the high-dose vs. HCM comparison were identified (Figure 2C and Figure 3B, respectively). In the low- vs. high-dose comparison, only one enriched term was found for downregulated DEG (toxin metabolic process) (Figure 5C). GO BP terms relative to negative regulation of cell proliferation, collagen fibril organization, extracellular matrix/structure organization, external encapsulating structure organization, angiogenesis, cellular response to organic cyclic compounds, fiber organization, blood vessel development, cell and biological adhesion, and anatomical structure formation involved in morphogenesis were identified for upregulated DEGs in the low-dose vs. HCM comparison (Figure 2D); of these, the cell and biological adhesion terms were shared with the high-dose vs. HCM comparison of upregulated DEGs, although additional terms of epithelial cell development, erythrocyte and myeloid homeostasis, pattern specification, myeloid cell differentiation, skeletal system development, and pattern specification process were present in this comparison (Figure 3C). When treatment groups were grouped (all doses vs. HCM), upregulated DEGs had enrichment for the following GO BP terms: acetylcholine, acetate ester, neurotransmitter metabolic processes, regulation of osteoclast differentiation, extracellular matrix and structure organization, and cell and biological adhesion (Figure 4B). Between the low- and high-dose comparison of upregulated DEGs, 16 terms encompassing extrinsic apoptotic signaling, myofibril assembly, muscle development, contraction, organization, and cytoskeleton organization were discovered (Figure 5D).

KEGG pathway terms relative to the renin–angiotensin system, metabolism of xenobiotics by cytochrome P450, chemical carcinogenesis, drug metabolism, tryptophan metabolism, and metabolic pathways were enriched for LV downregulated DEGs in the low-dose vs. HCM comparison (Figure 2E); however, they were not present in the high-dose vs. HCM groups. Instead, pathways pertaining to sodium, calcium, mineral, and carbohydrate absorption, cardiomyocyte adrenergic signaling, secretion (i.e., aldosterone and insulin), cardiac muscle contraction, and thyroid hormone synthesis were identified (Figure 3D). Only the extracellular matrix–receptor interaction term was enriched in LV upregulated DEGs for the low-dose vs. HCM comparison (Figure 2F), whereas none were identified in the high-dose vs. HCM comparison. Enrichment for dose-dependent comparisons (low- vs. high-dose) was identified for downregulated DEGs; three pathways were enriched, including the drug metabolism, phagosome, and fluid shear stress and atherosclerosis pathways (Figure 5E). No GO CC, GO BP, and KEGG pathway-enriched terms were found for downregulated DEGs in the all doses vs. HCM comparisons.

#### 3.4.2. Differentially Abundant Peptide Enrichment of the Left Ventricle

Fourteen GO CC, 12 GO BP, and 13 KEGG enrichment analyses for DAPs were identified out of the total 84 possible enrichments for proteomic comparisons (Supplemental Table S12).

**Figure 6:**
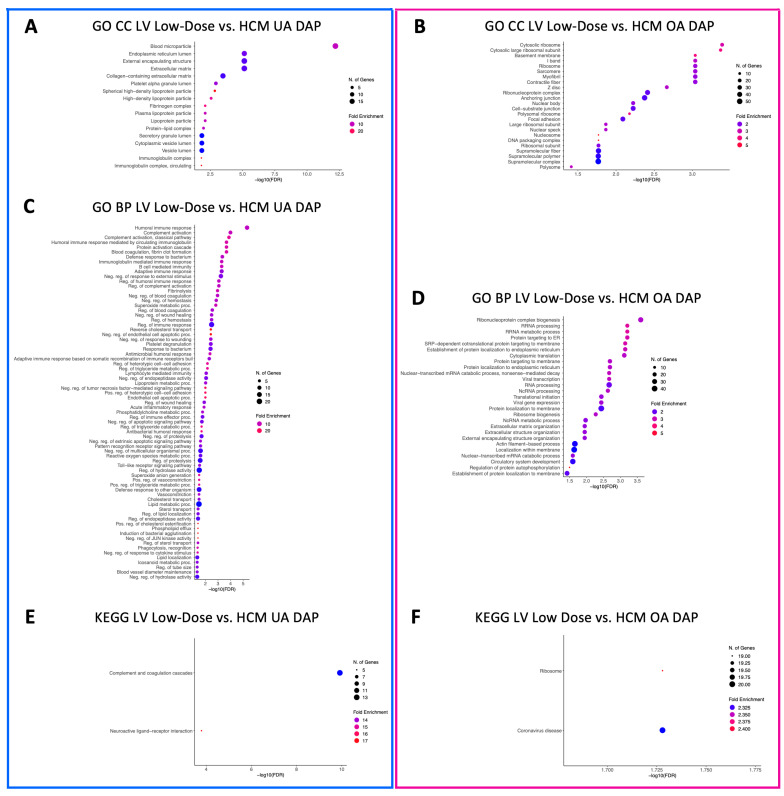
Enriched terms for LV DAPs in the low-dose vs. HCM comparison. Enrichment plots for GO CC (**A**,**B**), GO BP (**C**,**D**), and KEGG pathway (**E**,**F**) term analyses are presented for under- (blue box) and overabundant (pink box) DAPs. The *y*-axis depicts identified enriched terms, whereas the *x*-axis depicts the terms’ significance (−log10[FDR]). The terms are sorted according to statistical significance; the top-most terms on the *y*-axis constitute the most statistically significant of terms. The size of individual points depicts the total number of proteins binned to a given enriched term; high or low fold enrichment is represented by red or blue coloring, respectively. Abbreviations: HCM = hypertrophic cardiomyopathy, LV = left ventricle, GO = gene ontology, CC = cellular components, BP = biological processes, UA = underabundant, OA = overabundant, DAP = differentially abundant peptide(s).

**Figure 7:**
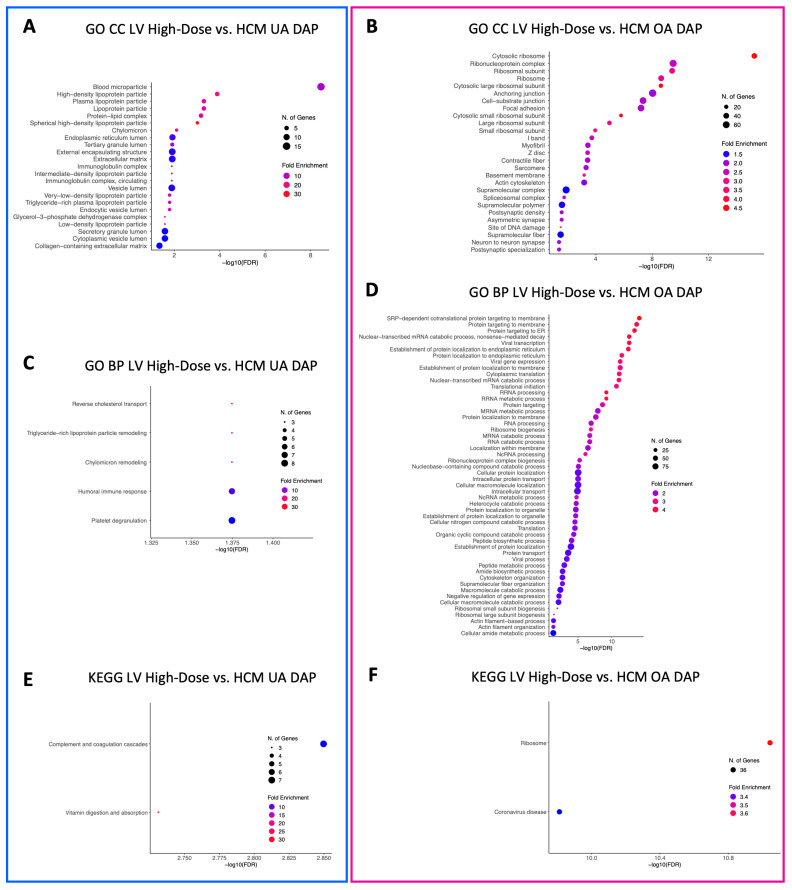
Enriched terms for LV DAPs in the high-dose vs. HCM comparison. Enrichment plots for GO CC (**A**,**B**), GO BP (**C**,**D**), and KEGG pathway (**E**,**F**) term analyses are presented for under- (blue box) and overabundant (pink box) DAPs. The *y*-axis depicts identified enriched terms, whereas the *x*-axis depicts the terms’ significance (−log10[FDR]). The terms are sorted according to statistical significance; the top-most terms on the *y*-axis constitute the most statistically significant of terms. The size of individual points depicts the total number of proteins binned to a given enriched term; high or low fold enrichment is represented by red or blue coloring, respectively. Abbreviations: HCM = hypertrophic cardiomyopathy, LV = left ventricle, GO = gene ontology, CC = cellular components, BP = biological processes, UA = underabundant, OA = overabundant, DAP = differentially abundant peptide(s).

**Figure 8:**
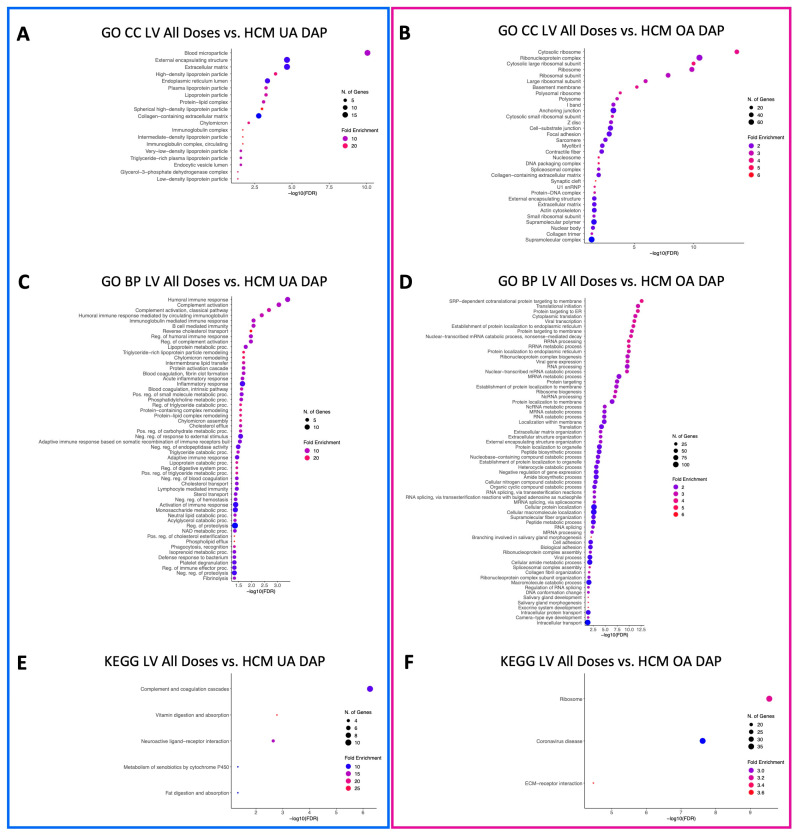
Enriched terms for LV DAPs in the pooled all doses vs. HCM comparison. Enrichment plots for GO CC (**A**,**B**), GO BP (**C**,**D**), and KEGG pathway (**E**,**F**) term analyses are presented for under- (blue box) and overabundant (pink box) DAPs. The *y*-axis depicts identified enriched terms, whereas the *x*-axis depicts the terms’ significance (−log10[FDR]). The terms are sorted according to statistical significance; the top-most terms on the *y*-axis constitute the most statistically significant of terms. The size of individual points depicts the total number of proteins binned to a given enriched term; high or low fold enrichment is represented by red or blue coloring, respectively. Abbreviations: HCM = hypertrophic cardiomyopathy, LV = left ventricle, GO = gene ontology, CC = cellular components, BP = biological processes, UA = underabundant, OA = overabundant, DAP = differentially abundant peptide(s).

GO CC terms relative to blood microparticles, the collagen-containing extracellular matrix, the cytoplasmic and endoplasmic vesicle lumen, the external encapsulating lumen, the extracellular matrix, lipoprotein particles, immunoglobulin particles, the protein–lipid complex, the secretory granule lumen, and the vesicle lumen were identified in LV-underabundant DAPs in both the low-dose vs. HCM and high-dose vs. HCM comparisons (Figure 6A and Figure 7A, respectively). When doses were grouped together, all but cytoplasmic, secretory, and vesicle lumen terms were no longer enriched for LV-underabundant DAPs; however, glycerol-3-phosphate dehydrogenase complex and endocytic vesicle lumen terms were newly identified (Figure 8A). Commonalities between the anchoring and cell-substrate junction, basement membrane, contractile fiber/myofibril, ribosomal subunits, focal adhesion, I-band, ribonucleoprotein complex, sarcomere, supramolecular complex fiber and polymer, and Z-disc component terms for overabundant DAPs were identified in both the low-dose vs. HCM and high-dose vs. HCM comparisons (Figure 6B and Figure 7B, respectively); interestingly, terms pertaining to the ribosome were most enriched in both of these comparisons. When both dose groups were pooled (all doses vs. HCM), all but the supramolecular fiber term were retained and nucleosome, DNA packing complex, polysome, collagen trimmer, spliceosome complex, extracellular matrix, nuclear body, and external encapsulating structure terms were gained for overabundant LV DAPs (Figure 8B).

A total of 73 enriched GO BP terms for underabundant DAPs were identified in the LV tissues in the low-dose vs. HCM comparison, whereas only five were identified in the high-dose vs. HCM comparison (Figure 6C and Figure 7C, respectively). Between these two comparisons, terms involving reverse cholesterol transport, humoral immune response, and platelet degranulation were shared. A total of 54 enriched terms were identified in the LV-underabundant all doses vs. HCM comparison (Figure 8C); 36 total terms were shared with the low-dose vs. HCM and high-dose vs. HCM comparison, mostly of cholesterol transport and esterification, lipoprotein remodeling and meta-/catabolic processes, phagocytosis, immune response, blood coagulation (i.e., fibrin clot formation and platelet degranulation), protein activation, endopeptidase activity, and negative regulation of proteolysis and hemostasis. Twenty-one shared terms between the low-dose vs. HCM and the high-dose vs. HCM comparison for overabundant DAPs, primarily of ncRNA and rRNA processing and metabolic processes, translation, endoplasmic reticulum processes, nonsense-mediated decay, transcription, ribosome biogenesis, and protein localization, were found (Figure 6D and Figure 7D, respectively); all 21 terms were retained when both dose groups were pooled against HCM control cats, and an additional 44 enriched terms were found of the same aforementioned commonalities in addition to spliceosome, catabolic processes, extracellular matrix, and amide biosynthesis (Figure 8D).

Two KEGG pathway terms were identified for the low-dose vs. HCM underabundant comparison (neuroactive ligand–receptor interaction and complement and coagulation cascades) (Figure 6E). Similarly, two KEGG pathways were discovered for the high-dose vs. HCM underabundant comparison, one pathway (complement and coagulation cascades) was shared between the low-dose vs. HCM comparison, whereas the vitamin digestion and absorption term was specific to this comparison (Figure 7E). When dose groups were pooled together (all doses vs. HCM), all but the vitamin digestion and absorption pathway term were no longer enriched in the underabundant DAP within both treatment groups when compared to HCM controls; instead, new terms of fat digestion, absorption, and metabolism of xenobiotics by cytochrome P450 were discovered (Figure 8E). Two KEGG terms (ribosome and coronavirus disease) were enriched in the overabundant DAPs in both the low-dose vs. HCM and high-dose vs. HCM comparisons (Figure 6F and Figure 7F, respectively) and both shared terms remained enriched when both treatment groups were pooled together, however, an additional KEGG term (ECM-receptor interaction) was identified for overabundant DAPs (Figure 8F).

No statistically significant enriched GO CC, GO BP, or KEGG pathway terms were identified in either under- or overabundant LV DAPs in the low-dose vs. high-dose comparison.

#### 3.4.3. Differentially Abundant Peptide Enrichment of Plasma and Urine

Of the 18 possible plasma enrichment analyses, only three resulted in the identification of enriched terms; these include GO CC, GO BP, and KEGG pathway analyses for when both treatment groups were pooled (all doses pre- and post-treatment) for overabundant DAPs. In the GO CC analysis, eight terms were enriched (lipoprotein particle [lipoprotein, very-low density, triglyceride-rich plasma, plasma, high-density], protein-lipid complex, chylomicron, and early endosome) (Supplemental Figure S12A). A total of 109 GO BP terms were enriched in this comparison, mostly pertaining to esterification, catabolic, metabolic, fatty acid biosynthetics, immunity, phospholipid biosynthetics, the digestive system, cholesterol biosynthetics, alcohol biosynthetic metabolism, and homeostatic processes, as well as cell-cell adhesion, lipid localization, lipoprotein particle assembly, and immune response (Supplemental Figure S12B). Four enriched KEGG pathway terms were found in this comparison (fat and vitamin digestion and absorption, cholesterol metabolism, lipids, and atherosclerosis) (Supplemental Figure S12C).

Only two analyses (GO CC) resulted in enriched terms, out of the 18 total possible urine pre- and post-treatment analyses; analyses that resulted in enriched terms were for overabundant DAPs in the low-dose pre- vs. post-treatment and when all doses were pooled together in the all doses pre- vs. post-treatment comparisons. In the low-dose pre- vs. post-treatment comparison, only the neuron projection terminus enriched term was identified (Supplemental Figure S13A); when treatment groups were pooled together (all doses pre- vs. post-treatment), this term was no longer enriched, instead, a new term (blood microparticle) was found (Supplemental Figure S14A).

### 3.5. Congruent Expression between -Omic Approaches in Cardiac Tissues

We sought to determine congruent expression of genes in both the transcript and protein level within the LV and IVS tissues of cats for each given comparison. In the low-dose vs. HCM comparison, *CES2* and *SERPINB2* were downregulated in the LV tissue, whereas *AS3MT* and *PHOSPHO1* were downregulated in the high-dose vs. HCM comparison. Twelve genes (*ANKRD1*, *ANKRD2*, *ASPN*, *DPYSL3*, *FBLN5*, *FHL1*, *LYPLA1*, *MSN*, *MYL1*, *PTGFRN*, *TGFBI*, and *XIRP2*) were upregulated in the low-dose vs. HCM comparison; when treatment groups were pooled (all doses vs. HCM), *ASPN*, *COL6A3*, *FBLN5*, and *TGFBI* remained upregulated between the -omic approaches. No other gene names were congruently expressed between LV transcriptomic and proteomic data. Only one gene (*FBLN5*) was upregulated in both techniques for IVS tissue in the all doses vs. HCM comparison. Interestingly, *FBLN5* was the only gene that remained upregulated across both cardiac tissues (Table 3). Taken together, these gene and peptide expression commonalities across -omic techniques reinforce the previously identified impacts of rapamycin on the extracellular matrix, coagulation, metabolism, lipid homeostasis, and autophagy.

## 4. Discussion

It is well accepted that HCM results in myocardial hypertrophy and resulting adverse cardiac remodeling. The distinct mechanism of action by which mTOR inhibition reduces cardiac hypertrophy remains to be fully elucidated; however, it has been previously demonstrated that inhibition of mTOR reduces cellular anabolic activities. In pressure-overload models, selective inhibition of mTORC1 has been shown to reduce cardiac hypertrophy [5,6,7,34].

In comparison with the high-dose treatment group, low-dose LV tissues demonstrated markedly upregulated DEGs associated with myofibrillar and contractile cellular components, consistent with greater suppression of myocardial hypertrophy by the higher 0.30 mg/kg dose.

Downregulation of phagolysosome and phagocytic vesicle cellular components in the lower-dose group, relative to the higher-dose group, likely reflect dose-responsive effects on autophagy, the process by which damaged or dysfunctional cellular components undergo lysosomal degradation. Autophagic activity increases in response to mTOR suppression as part of a physiologic starvation response; decreased autophagy is well established as a feature of cardiac hypertrophy in animals and humans; therefore, activation of autophagy may ameliorate cardiomyopathy in cats, as previously seen in a *Mybpc3*-targeted knock-in mouse model [8].

Inefficiencies in sarcomeric contraction and metabolic changes have previously been reported in hypertrophied cardiomyocytes of humans and mouse models [35,36]. Increased ATP utilization in HCM-affected animals lead to impaired cellular mechanisms for regulating calcium, homeostasis, and metabolism, leading to sarcomeric relaxation inabilities [37]. Cardiomyocytes generate adenosine triphosphate (ATP) via substrate metabolism, mostly of fatty acids, carbohydrates, and amino acids; tissue-level differential expression of genes and peptides of catabolic and metabolic commonalities may represent cardiac remodeling occurring at the tissue level by homeostatic mechanisms and changes in substrate adaptations [38]. An overabundance of plasma proteins associated with fat and cholesterol metabolism (APOA1, APO41, APOC4, and VNN1) was observed when pre- and post-treatment samples were compared in the treated animals, consistent with the expected metabolic effects of rapamycin in mouse models [39]. Additionally, the role of impaired fatty acid oxidation and shifts toward glucose metabolism in ischemic heart disease and across many stages of hypertrophic cardiomyopathy is reported [40,41]. Our data suggests enrichment of biological processes associated with fatty acid metabolism in plasma, perhaps signaling at least a partial shift from glucose reliance to fatty acid metabolism toward restoration of normal phenotypes. This finding warrants future investigation.

In comparison with the tissues of untreated cats, the LV tissues of treated cats expressed higher levels of genes associated with the extracellular matrix and structures and ribosomal protein, and may represent a potential signal associated with myocardial remodeling toward amelioration of the HCM phenotype; however, this pattern may also be reflective of differences in the disease phenotype between treated and untreated cats due to their varied age.

An underabundance of proteins associated with complement activation, humoral immunity, and inflammatory responses may suggest an anti-inflammatory effect of rapamycin in these tissues; this may reflect a direct disease-modifying effect or a degree of immune suppression. However, there were no clinical or clinicopathological findings to suggest immune suppression. Although rapamycin is employed therapeutically as an immunosuppressant in humans under high-dose regimens, low or intermittent dosing with rapamycin or other mTOR inhibitors has, on the contrary, been demonstrated to enhance vaccine responses and resistance to challenge in cell cultures and live animals, including humans [42,43,44,45]. There is a paucity of evidence to support that the dose regimens employed in the current study would exert immunosuppressive effects; the observed differences in complement, immune, and inflammatory pathways are noteworthy findings.

This study has several limitations for consideration. For the purpose of this initial, exploratory, multi-omic experiment, we aimed to perform preliminary comparisons solely to study tissue-level effects of rapamycin treatment in HCM-diagnosed cats. Special focus on the balance of sex, weight, and severity of HCM was given to the study design; however, the ages of the HCM control cats used in this study are notably greater than the cats that received low- or high-dose rapamycin, and represents a limitation of this study’s design principally due to the extreme value and limited availability of this unique large animal model of HCM. At the time of study design, no opportunity to identify additional young animals with HCM suitable for a terminal experiment and no availability to identify older animals for the treatment groups was possible; due to this limited availability of younger HCM phenotype-positive colony cats, along with the authors’ intent to limit the terminal use of cats wherever possible, the tissues of the three HCM control cats used for this study were obtained as part of previous studies, and only six cats receiving rapamycin treatment were specifically selected for this study.

Though the samples from the HCM control population are older than the cats in the low- and high-dose treatment group, all cats used in this study are categorically considered to be mature cats by the American Association of Feline Practitioners (AAFP): seniors when >11 years, geriatric when >15 years, and mature when >1 and <11 (the range that all cats in this study fall within). Importantly, the cats in this study represented both sexes within all treatment groups and were all unaltered (sexually intact). Additionally, we have demonstrated sample homogeneity across treated and untreated cats through H&E and Masson’s trichrome staining. Coupled with our histopathologic and echocardiographic assessments, HCM control cats in this study represent a mature cat population with a tight molecular and gross disease phenotype (HCM stage B1). In other words, cats in the untreated group remained subclinical for HCM and it is important to note that no other differences between the treated and untreated cats, in terms of clinical signs of disease, HCM phenotype, or disease outcome was apparent, perhaps imparting a more stable disease phenotype when compared to the younger cats with HCM [46]. However, this remains a limitation of the study to be addressed in future work.

All cats used in this study are considered equal representatives of their HCM disease status, both echocardiographically and histopathologically and regardless of age. Despite the imbalance in age, overlapping between target pathways previously shown to play a role in HCM and to be impacted by intermittent mTORC1 inhibition was identified in this study. For example, when compared to the LV and IVS tissues of untreated cats, rapamycin-treated counterparts expressed overabundant DAPs with known metabolic activity ontologies. Autophagic and metabolic protein expression is canonically decreased in aged individuals [47,48,49,50]. In our study, rapamycin treatment led to statistically significant decreased tissue-level expression of these proteins. Results such as this highlight statistically significant, biologically relevant, and consistent changes with previously published results on rapamycin-treatment effects and emphasize the reliability of the results presented here. For example, despite imbalances in age between groups, our proteomics data revealed statistically significant increased levels of metabolic proteins and, therefore, enriched pathways associated with autophagic flux after rapamycin treatment in younger cats where we would expect the opposite to be true, thus, highlighting rapamycin treatment effects on autophagy.

In a previous nonpivotal exploratory clinical trial aiming to assess the short-term effects of intermittent, delayed-release, once-weekly, rapamycin dosing in cats affected by non-obstructive subclinical HCM, we observed that a 0.30 mg/kg dose resulted in LV remodeling, supported by a decrease in LV MWT when compared to a placebo (*p* < 0.01) at the Day 180 timepoint [30]. However, in this much shorter and smaller study, we did not observe statistically significant changes in echocardiographic parameters from the screening to Day 56 timepoints. Thus, the authors speculate that this study’s duration or a type-II error may cloud our ability to appreciate morphologic changes related to treatment. Although markers of reverse myocardial remodeling were identified via our RNA-seq and LCMS methods after rapamycin treatment, maintaining these expression profiles is necessary to invoke beneficial pathologic change. In a long-term (6–18 week) aortic banding pressure overload-induced rat model, regression of LV hypertrophy following the removal of the aortic band was not echocardiographically apparent until 12 weeks and was not related to the exposure time of the pressure overload [51]. This study supports our conclusion that future studies involving long-term intermittent low-dose rapamycin dosing are needed to fully understand the time course of expected myocardial remodeling. Alternatively, the addition of more sensitive imaging modalities (e.g., cardiac magnetic resonance imaging) may aid in the earlier detection of reverse remodeling that would otherwise remain echocardiographically silent.

Rapamycin has been previously shown to enhance tissue factor (TF) activity, leading to an increased risk of thromboembolic events [52,53]; however, studies have also shown that rapamycin treatment helps mitigate coagulopathies via D-dimer reduction [54,55]. To the authors’ knowledge, studies looking at the pre- vs. post-treatment effects of low intermittent rapamycin on blood in a large non-human model organism have not been published. In our study, a significant underabundance of complement and coagulation cascade proteins was observed in the LV tissues of treated cats in comparison with controls. For example, Von Willebrand Factor (VWF), a primary hemostatic protein, was found to be downregulated in our plasma dataset. Given the propensity of cats with advanced subclinical HCM and left atrial enlargement to experience arterial thromboembolism (ATE), and the established/expected clinical benefits of clopidogrel and other antithrombotic agents in these cats, the observed dose-dependent decrease in VWF protein after rapamycin treatment supports that rapamycin may be protective of not only maladaptive hypertrophy, but of at least one of the disease’s outcomes as well (i.e., ATEs) [56,57,58,59]. This result highlights the potential of future studies using rapamycin, fills knowledge gaps over rapamycin’s potential role in coagulopathies and thrombopathies, and is a further interesting observation of potential therapeutic relevance in veterinary and human patients.

Despite the sample size per group, successful differential expression studies using as little as six to eight biological replicates have been performed in domesticated animals [60,61]. A survey of best practices for RNA-seq data analysis suggests that three biological replicates is a sufficient number of samples per group to detect truly biologically relevant results [62]. An additional report suggests that a minimum of three biological replicates in each group is necessary to achieve statistical significance [63]. In this study, we performed RNA-seq at a targeted average of 50 million reads per sample; this above-average read count results in a more reliable RNA-seq experiment, particularly compared to those that are not aimed to detect low-abundance transcripts [64].

A majority of the DEG/DAP conclusions data was rendered from the treatment-related comparisons (treated vs. untreated). Though differential expressions/abundances of genes and peptides were found in the low- vs. high-dose comparisons, conclusions were drawn from enriched pathways for the clustering of gene names that were either up- or downregulated within a given transcriptomic or proteomic comparison rather than biasing conclusions from a priori selected genes. With this said, enriched terms for low- vs. high-dose comparisons were identified for only six out of the 24 possible comparisons listed in Supplemental Table S12; dose-dependent terms deemed biologically and clinically relevant by the authors were discussed in Section 4. However, more robust future follow-up studies, with a greater focus on dose-related differences, are needed and likely to proceed from this study.

Mitochondrial dysfunction and impaired autophagic flux are known components of myocardial remodeling and likely modulated the HCM disease severity in the cat. Specifically, impaired cardiac mitochondrial oxidative phosphorylation (OXPHOS) and stress has been noted in cats afflicted by HCM [65]. Given the variability of RNA and protein yield from feline heart tissues and a low population size requiring a greater than normal RNA-seq target of 50 million/reads per sample for accurate differential analyses, significant IVS and LV tissue quantities of all study subjects were used in this study. Unfortunately, the remainder tissue samples were not enough to create a reliable library of cells for subsequent mitochondrial function (i.e., Agilent SeaHorse Cell-Mito Stress Test) or to extract high-quality RNA/protein for RTqPCR/Western blot analyses of autophagy on the most promising markers (e.g., LC3, mTOR, and p62). However, the observed downregulation of phagolysosome and phagocytic vesicle cellular components in the lower-dose group relative to the higher-dose group previously highlighted may be used as a proxy for future efforts aiming to validate the results presented here.

Lastly, only minimal proteomic changes were observed in the urine and plasma samples of cats pre- and post-treatment with rapamycin. This more subtle proteomic treatment effect could be explained by the comparatively lower rapamycin dose used in comparison to what is commonly seen in studies involving mouse models. However, our small sample size cannot be ruled out as a contributing factor, further representing the need for continued research efforts.

Taking all of the aforementioned findings together, the present study represents a pilot study interrogating the tissue-level, blood, and urine effects of rapamycin dosing in subclinical HCM-affected cat colony cats. The results presented here establish the need for future validation studies aiming to interrogate such findings at a greater resolution. These studies are likely to investigate myocardial effects of rapamycin treatment on the most compelling DEGs/DAPs reported here (Table 3) using fine-scale molecular (i.e., Western blot, flow cytometry, targeted LCMS) and genetic (RT-qPCR, single-cell RNA-seq, and targeted RNA-Seq) approaches in a large-scale population of naturally occurring animal models of HCM.

## 5. Conclusions

This is the first study to report multi-omic tissue, plasma, and urine findings from cats with subclinical HCM. Following repeated weekly dosing with a novel DR rapamycin product, transcriptomic differences between the low- and high-dose groups support dose-responsive suppressive effects on myocardial hypertrophy and stimulatory effects on autophagy. Differences in the myocardial proteome between treated and control cats suggest potential anti-coagulant/-thrombotic effects of the drug that may be of further therapeutic benefit and warrant further investigation. Other than alterations in lipid metabolism, there were no significant differences in plasma or urinary analyses to suggest adverse effects of DR rapamycin when administered at 0.15 or 0.30 mg/kg once weekly.

## Figures and Tables

**Table 1:** Total global DEGs of LV and IVS tissues. Global LV and IVS DEGs of all study comparisons are reported. Bolded gene symbols represent shared DEGs between low-dose vs. HCM and high-dose vs. HCM comparisons per tissue type. All down- (log2FoldChange < −1) and upregulated (log2FoldChange > 1) DEGs reported here were statistically significant (*p*-value_adjusted_ < 0.05). * Down-/upregulation was assigned respective to the asterisk-marked groups in the comparison column. Abbreviations: HCM = hypertrophic cardiomyopathy, DEG = differentially expressed gene(s), LV = left ventricle, IVS = interventricular septum.

	LV		IVS	
*Comparison*	Downregulated	Upregulated	Downregulated	Upregulated
Low-Dose * vs. *HCM*	*ACMSD, ACSL5, ANPEP, AOC1, ATP6V1B1, CDO1, CES2, CPAMD8, CSRP2, CUBN, CYP4F8, DHDH, DHRS11, ECRG4, ENPEP, ERCC6L, **FAM177B**, FMO1, G6PC, HAAO, HAO2, HJV, HPD, HSD11B1, IFT27, INCENP, IZUMO2, **KCNJ3**, LRP2, MGST1, MLPH, MME, MOGAT3, NAPSA, NR1I3, P2RX1, PDZK1, PIGR, **PPDPFL**, RAB7B, RASGEF1A, **RPS6KA5**, **SBK3**, SERPINB2, SGPP2, SLC12A3, SLC13A3, SLC22A1, SLC22A11, SLC25A26, SLC45A3, SLC5A2, SLC6A18, SLC6A19, SLC6A2, **SPATA22**, TAAR1, TLCD4, TPRN, **TRPV3**, UMOD, UPB1* (n = 62) **(n = 7)**	*ACHE, ADAM33, ADAMTS14, ADAMTS17, **AFF3**, AMPH, ANGPTL2, ANKRD1, ANKRD2, ASPN, BAZ1A, **BICC1**, CACNA1S, CCDC136, CCL2, CCN2, CLMP, CLU, CNN1, CNTN1, COL12A1, COL23A1, COL4A1, COL6A3, CSAD, CSPG5, CTNND2, CTXN1, CYLD, DCLK1, DLK2, DPYSL3, DSEL, DYNLL1, EGR1, EHBP1L1, EPHA3, FAP, FAT1, FBLN1, FBLN5, FHL1, **FREM1**, FSHR, GALNT16, GAS1, **GNAI1**, GPR153, GPR158, **GRAP2**, HTRA1, IGF2BP3, IGFBP5, **ITGB8**, ITIH5, KERA, **KLF5**, KRT80, LOXL1, LOXL2, **LRRC17**, LYPLA1, **MOXD1**, MRC2, MSN, MYL1, MYOT, NIBAN1, NMT2, NPPB, NPR3, NXPH3, PAK1, PALD1, PCDH9, PEX5L, PGAP4, PHEX, PLCL1, PODN, PSAT1, PTGFRN, RAB17, RET, ROBO3, **SDC2**, SEMA3B, SEPTIN6, SLC14A1, SLC22A15, SLC38A1, SLC4A7, SMOC2, SMTNL2, SNAP91, SOBP, SPP1, SUCNR1, SULT1C4, SV2C, SYNPO2, TGFB2, TGFBI, TMEM178A, TRIM2, TRIM36, TSHZ3, VTN, WSCD2, XIRP2* (n = 110) **(n = 10)**	*ECRG4, FABP9, GPR75, IFI6, L3MBTL4, P2RX1, TAAR1, TENT5B, **TESC*** (n = 9) **(n = 1)**	*CASQ1, DCLK1, **IGSF10**, JPH4, **KERA**, MYL1, PHYHIP, SLC1A3, SPOCK1, VTN* (n = 10) **(n = 2)**
High-Dose * vs. *HCM*	*ADAMTS3, ANKRD42, AS3MT, ATF3, ATP1A4, ATP1B4, C10orf90, CPLX1, CXCL14, DBX2, **FAM177B**, FGF11, FRMPD1, HOPX, HSD17B8, IFI6, IRX3, IRX5, **KCNJ3**, MTRF1, MYH13, NQO1, OTUD1, PHOSPHO1, **PPDPFL**, PYROXD2, RCAN1, **RPS6KA5**, **SBK3**, SLC38A2, SLC6A6, **SPATA22**, TAS2R9, TENT5B, TMEM176B, TRABD2B, **TRPV3**, UNC5CL* (n = 38) **(n = 7)**	***AFF3**, **BICC1**, CDH1, CDK6, CLDN4, CNGA1, COL14A1, DDAH1, DIPK2A, ELF3, EPPK1, **FREM1**, **GNAI1**, **GRAP2**, HOXA5, HOXB6, HOXB7, **ITGB8**, KIRREL1, KIT, **KLF5**, **LRRC17**, **MOXD1**, N4BP3, NAV1, P4HA3, PAWR, PBX3, PCSK6, **SDC2**, STON2, TSLP, VSTM2A, WDR36* (n = 36) **(n = 10)**	*AS3MT, BTNL9, CCN3, CDO1, ETNPPL, FAM177B, FTCD, GNAO1, HSD17B8, HVCN1, INCENP, KLHL33, MAMDC4, NCF2, PDE4A, PPDPFL, SCN10A, **TESC**, TMEM145, TRIM72* (n = 20) **(n = 1)**	*ABCA9, ADA, BPHL, C7, CACNA2D2, CCDC159, CNGA1, EEF1AKMT1, FBLN5, FBN2, FREM1, GRAP2, **IGSF10**, IRX5, ITGB8, KCNG1, **KERA**, KIT, MEIS1, MMP7, MYOT, PDGFRA, PLTP, RIMKLB, SLC1A6, SNAP91, SVEP1, TCFL5, VSIG10* (n = 29) **(n = 2)**
All Doses * vs. *HCM*	*ANKRD24, ECRG4, FGF11, HOPX, IFI6, IFT27, KCNJ3, MTRF1, MYH13, NR2F6, PHACTR1, PPDPFL, RAB7B, RPS6KA5, SBK2, SBK3, SPATA22, TLCD4, TRPV3* (n = 19)	*ACHE, AFF3, ASPN, BAZ1A, BICC1, CLMP, COL6A3, DIPK2A, EPPK1, FAP, FBLN5, FREM1, FSHR, GNAI1, GPR153, GPR158, GRAP2, ITGB8, KIRREL1, KLF5, LRRC17, LRRC71, MOXD1, MRC2, PAWR, PODN, SDC2, SEMA3B, SLC4A7, SOBP, TGFB2, TGFBI, TMEM178A* (n = 33)	*ETNPPL, FAM177B, TESC* (n = 3)	*BPHL, FBLN5, FREM1, IGSF10, ITGB8, JPH4, KCNG1, KERA, PLTP, SOX11, SPOCK1, TAC3* (n = 12)
Low- * vs. *High-Dose*	*AFF3, CIDEC, CNGA1, FASN, FMO1, MGST1, NCF2, NIPAL1, OPN4, RAB7B, SLC22A1, TSHR* (n = 12)	*ANKRD2, APBA1, ATF3, ATP1A4, CACNA1S, CASQ1, CCN2, CNN1, COL23A1, CREB5, CSPG4, CXCL14, CYLD, DIPK1A, DPYSL3, EHBP1L1, FHL1, FSTL3, GADL1, IQCA1L, IRX5, LDHA, LYPLA1, NR2C1, NRAP, PALD1, PDLIM3, RAB17, RCAN1, SFRP4, SLC37A3, SYNPO2, TGFB2, TNFRSF12A, TNNT1, TSPAN2, XIRP2* (n = 37)	- (n = 0)	*DIPK1A* (n = 1)

**Table 2:** Shared under- and overabundant DAPs between LV and IVS tissues. The total number of shared DAPs between LV and IVS tissues are presented for each group comparison. * Under-/overabundance was assigned respective to the asterisk-marked groups in the comparison column. Abbreviations: HCM = hypertrophic cardiomyopathy, DAP = differentially abundant peptide(s), LV = left ventricle, IVS = interventricular septum.

*Comparison*	Underabundant	Overabundant
Low-Dose * **vs. *HCM***	ACOT4, AFM, ALDH1A1, APOA1, APOA4, AS3MT, C3, C5, CES2, CES5A, CLU, COL28A1, CP, CPN2, CRP, CTSD, EPHX2, F13A1, FUOM, GSTK1, GSTP1, HP, HYI, IGHM, IGLL5, ITIH2, JCHAIN, KNG1, MDH1, MIA3, MYBPC3, NOL3, PON1, PSAP, S100A1, SERPINB2, SERPIND1, SERPING1 (n = 38)	ABCC9, AGK, ANKRD2, AP1G1, APEX1, ATP2B4, BANF1, BCKDK, BDH1, BPHL, CAMK2A, CAVIN4, CD59, CD9, CLPX, COBL, COL15A1, DDRGK1, DNAJB4, DNAJC11, DPYSL3, EIF3H, EIF3I, EIF4A3, ENAH, EPB41L3, FBLN5, FHL1, FLII, FLNC, H1-0, H1-4, H2AX, H2B, H3F3A, HNRNPA3, HNRNPR, HNRNPUL2, HP1BP3, HSPG2, HYOU1, IDH3B, ITIH3, LAMA2, LAMA4, LAMA5, LAMB1, LIMS1, LIPA, LMNB1, LMO7, LOC101083645, MACF1, MACROH2A1, MAPT, METTL7A, MLF1, MTFR1L, MVP, MYADM, MYO18A, MYO1B, NDEL1, NDRG1, NEXN, NID2, NONO, NRAP, NUDT21, PALLD, PARP1, PARVA, PPM1K, PPP2R5A, PTGFRN, PXN, RAP2C, RBMXL1, RPL10A, RPL13, RPL14, RPL15, RPL22, RPL23, RPL24, RPL27A, RPL30, RPL4, RPS18, RPS21, RPS23, RPS24, RPS6, RPS6KA3, RRAD, SLC2A1, SNRPD1, SNRPE, SORBS1, SORBS2, SRSF1, SUN2, SYNPO, SYNPO2L, TGFBI, TLN2, TOMM20, TRIM54, U2AF1, XIRP1, XIRP2 (n = 111)
High-Dose * **vs. *HCM***	AS3MT, CLU, CPT2, CTSD, GPD1L, GSTO1, IGLL5, ITIH2, KNG1, NCEH1, PSAP, S100A1, SERPINB2 (n = 13)	ADSL, ANKRD1, ANXA7, ARPC2, BANF1, CD81, CKAP4, CLPX, COBL, COL15A1, COL18A1, COPB1, COPG1, CTNNA2, DAG1, DNAJA1, DSC2, DSG2, DYNC1LI2, EIF3D, EPB41L2, ERP29, FARSB, FHL1, FLII, FLOT1, GTF2I, H1-10, H1-4, H2B, H3F3A, HNRNPA1, HNRNPA3, HNRNPF, HNRNPL, HNRNPM, HNRNPUL2, HSPB3, HSPG2, HYOU1, IDH3B, ILF2, KIF13B, LGALS3, LMNB1, LYPLA1, MACF1, MCAM, METTL7A, MRPL39, MTFR1L, MVP, MYADM, NARS1, NDEL1, NDUFAF2, NEXN, NONO, NRAP, PARVA, PCYOX1, POGLUT3, PPIB, PRPF8, PTGFRN, PXN, RPL10, RPL14, RPL15, RPL22, RPL24, RPL7A, RPS11, RPS12, RPS23, RPS24, RPS28, RPS3A, RPS6, RRAD, RTN4, RUVBL2, SAE1, SDCBP, SEC22B, SERBP1, SNRPD1, SORBS1, SRI, SRSF1, STBD1, SUCLG2, SUN2, SVIL, SYNCRIP, SYNPO2L, TMED2, TRIM54, U2AF1 (n = 99)
All Doses * **vs. *HCM***	AFM, APOA1, APOA4, AS3MT, ATPAF1, CES5A, CLPTM1, CLU, COL28A1, CP, CTSD, F13A1, FUOM, GPD1L, GSTK1, GSTP1, HP, IGHM, IGLL5, ITIH2, JCHAIN, KNG1, MDH1, MYBPC3, NNMT, PON1, PSAP, S100A1, SERPINB2 (n = 29)	ABCC9, ABLIM1, AGK, AGRN, ANKRD1, ANKRD2, ANXA7, APEX1, ATP2B4, BANF1, BCKDK, BPHL, CA3, CAMK2A, CAPG, CAVIN4, CD44, CD59, CD81, CD9, CKAP4, CLPX, COBL, COL15A1, COL18A1, COL4A1, COL4A2, COL6A1, COL6A2, COMTD1, COPB1, CSPG4, CTNNA1, CTNNA2, CYB5A, DAG1, DDRGK1, DHX9, DNAJB4, DNAJC11, DNAJC8, DPYSL3, DSG2, DYNC1LI2, ECHDC3, EFEMP1, ENAH, EPB41L2, EPB41L3, FAH, FARSB, FBLN5, FBN1, FHL1, FLII, FLOT1, GNB1, GNG12, H1-0, H1-10, H1-4, H2A, H2AX, H2B, H3F3A, HACD1, HDAC2, HMGB1, HNRNPA1, HNRNPA3, HNRNPAB, HNRNPR, HNRNPUL2, HP1BP3, HSPB3, HSPG2, HYOU1, IDH3B, ILF2, ITGA7, KIF13B, KTN1, LAMA4, LAMA5, LAMB1, LDB3, LGALS3, LMNB1, LMO7, LOC101083645, LYPLA1, MACF1, MACROH2A1, MAP1B, MAPT, MCAM, METTL7A, MLF1, MLIP, MOCS1, MSI2, MSN, MTFR1L, MVP, MYADM, MYO1B, MYO1D, NARS1, NDEL1, NDUFAF2, NDUFV3, NES, NEXN, NFIA, NIBAN1, NID2, NONO, NRAP, OBSL1, OMA1, PARP1, PARVA, PCYOX1, PDLIM3, PLGRKT, PLN, POGLUT3, POSTN, PPIB, PPM1B, PPP2R5A, PRNP, PRPF8, PTBP1, PTGFRN, PXN, RAP2C, RBM39, RBMXL1, RPL10, RPL10A, RPL14, RPL15, RPL18, RPL22, RPL23A, RPL24, RPL27A, RPL30, RPL31, RPL39, RPL7A, RPS11, RPS12, RPS15, RPS15A, RPS21, RPS23, RPS24, RPS6, RPS6KA3, RPS8, RRAD, RRBP1, RUVBL2, SAFB, SDCBP, SEMA3C, SERBP1, SF1, SLC2A1, SNRNP70, SNRPD1, SNRPE, SORBS1, SORBS2, SORT1, SRSF1, SSPN, STBD1, SUN2, SVIL, SYNCRIP, SYNPO, SYNPO2L, TGFBI, TIMM29, TINAGL1, TLN2, TMED2, TNKS1BP1, TNXB, TOMM20, TRIM54, TRIM63, TRIP6, TUBB, TXNDC5, U2AF1, USP28, VASP, VCAN, VNN1, XIRP1, XIRP2, YBX1 (n = 206)
Low- * **vs. *High-Dose***	- (n = 0)	- (n = 0)

**Table 3:** Congruent transcript and protein expression of differentially expressed genes/peptides in LV and IVS tissues. The total number of genes/peptides congruently differentially expressed in LV and IVS tissues are presented for each group comparison. Bolded gene symbols represent shared genes/peptides between LV and IVS tissues. * Under-/overabundance was assigned respective to the asterisk-marked groups in the comparison column. Abbreviations: HCM = hypertrophic cardiomyopathy, LV = left ventricle, IVS = interventricular septum.

*Comparison*	LV		IVS	
	Underexpression	Overexpression	Underexpression	Overexpression
Low-Dose * **vs. *HCM***	CES2, SERPINB2 (n = 2) **(n = 0)**	ANKRD1, ANKRD2, ASPN, DPYSL3, FBLN5, FHL1, LYPLA1, MSN, MYL1, PTGFRN, TGFBI, XIRP2 (n = 12) **(n = 0)**	- (n = 0) **(n = 0)**	- (n = 0) **(n = 0)**
High-Dose * **vs. *HCM***	AS3MT, PHOSPHO1 (n = 2) **(n = 0)**	- (n = 0) **(n = 0)**	- (n = 0) **(n = 0)**	- (n = 0) **(n = 0)**
All Doses * **vs. *HCM***	- (n = 0) **(n = 0)**	ASPN, COL6A3, **FBLN5**, TGFBI (n = 4) **(n = 1)**	- (n = 0) **(n = 0)**	**FBLN5** (n = 1) **(n = 1)**
Low- * **vs. *High-Dose***	- (n = 0) **(n = 0)**	- (n = 0) **(n = 0)**	- (n = 0) **(n = 0)**	- (n = 0) **(n = 0)**

## Data Availability

Raw RNA-Seq (LV and IVS) and LCMS (LV, IVS, plasma, and urine) files for all study subjects are available in a publicly accessible data repository (https://doi.org/10.25338/B82P9D, accessed 9 August 2023). Additional data is available upon reasonable request from the corresponding author.

## Supplementary Materials

The following supporting information can be downloaded at: https://www.mdpi.com/article/10.3390/ani13203184/s1, Table S1: Subject information and affection status; Table S2: Study design and schedule of events; Table S3: Age and physical exam findings; Table S4: Urinalysis findings on rapamycin-treated cats; Table S5: Biochemistry findings on rapamycin-treated cats; Table S6: Hematology findings on rapamycin-treated cats; Table S7: Histopathologic findings for treatment and HCM control cats; Table S8: Echocardiographic findings for treatment cats; Table S9: Shared down- and upregulated DEGs between LV and IVS tissues; Table S10: Total global DAPs of LV and IVS tissues; Table S11: Total global DAPs of plasma and urine samples; Table S12: Total GO and KEGG term analyses on proteomic and transcriptomic comparisons; Figure S1: Methodology and proteomic comparisons of pre- and post-treatment urine and plasma samples; Figure S2: Key echocardiographic measurements and treatment-related changes; Figure S3: Statistically significant physical exam and biochemistry variables; Figure S4: Representative H&E staining of all nine study cats; Figure S5: Representative Masson’s trichrome staining of all nine study cats; Figure S6: Enriched terms for IVS DEGs in the low-dose vs HCM comparison; Figure S7: Enriched terms for IVS DAPs in the low-dose vs HCM comparison; Figure S8: Enriched terms for IVS DEGs in the high-dose vs HCM comparison; Figure S9: Enriched terms for IVS DAPs in the high-dose vs. HCM comparison; Figure S10: Enriched terms for IVS DEGs in the pooled all dose vs HCM comparison; Figure S11: Enriched terms for IVS DAPs in the pooled all doses vs HCM comparison; Figure S12: Enriched terms for plasma pre- and post-treatment DAPs in the pooled all doses group; Figure S13: Enriched term for urine pre- and post-treatment DAPs in the low-dose group; Figure S14: Enriched term for urine pre- and post-treatment DAPs in the pooled all doses group.
