# Supplementary material for: Multi-Omic, Histopathologic, and Clinicopathologic Effects of Once-Weekly Oral Rapamycin in a Naturally Occurring Feline Model of Hypertrophic Cardiomyopathy: A Pilot Study
Source: Animals (Basel). 2023 Oct 12;13(20):3184. doi: 10.3390/ani13203184 (PMC10603660; doi:10.3390/ani13203184)
Supplement: Supplementary file 1 [file animals-13-03184-s001.zip › animals-2579698-supplementary.pdf]

SUPPLEMENTAL TABLES

Subject	**A31P Genotype**	**Disease Status**	**Treatment Group**	**Given Dose**	**Sex**	**Age**
Cat 1	Heterozygous	HCM ACVIM stage B1	HCM control	-	M	9.99
Cat 2	Wildtype	HCM ACVIM stage B1	HCM control	-	M	9.97
Cat 3	Heterozygous	HCM ACVIM stage B1	HCM control	-	M	11.11
Cat 4	Heterozygous	HCM ACVIM stage B1	High-Dose	0.24mg/kg	M	1.92
Cat 5	Homozygous	HCM ACVIM stage B1	High-Dose	0.31mg/kg	M	1.87
Cat 6	Homozygous	HCM ACVIM stage B1	High-Dose	0.30mg/kg	F	1.47
Cat 7	Homozygous	HCM ACVIM stage B1	Low-Dose	0.13mg/kg	F	3.39
Cat 8	Homozygous	HCM ACVIM stage B1	Low-Dose	0.13mg/kg	M	1.47
Cat 9	Homozygous	HCM ACVIM stage B1	Low-Dose	0.15mg/kg	F	1.67

Supplemental Table S1. Subject information and affection status. The *MYBPC3* A31P genotype, disease status, treatment allocation, DR rapamycin dose, sex, and age for each cat used in this study are presented.
Abbreviations: *MYBPC3 = myosin-binding protein C3*, DR = delayed-release, HCM = hypertrophic cardiomyopathy, ACVIM = American college of veterinary internal medicine.

Event	**Screening**	**Day 0**	**Day 1-55; 57, 58, 59**	**Day 56**	**Day 60**	**Post In-Life**
Physical Exam	**X**			**X**		
Echocardiogram	**X**			**X**		
Electrocardiogram	**X**			**X**		
Clinical Pathology	**X**			**X**		
Urinalysis	**X**			**X**		
Blood Collection (Proteomics)	**X**			**X**		
Urine Collection (Proteomics)	**X**			**X**		
Randomization		**X**				
Weekly Dosing		**X**	**X**	**X**		
Body Weight	**X**			**X**	**X**	**X**
Daily Observations			**X**			
Euthanasia					**X**	
Necropsy					**X**	
Proteomics & RNA-Seq Analysis						**X**

Supplemental Table S2. Study design and schedule of events. Events for each study timepoint are presented.

Subject	Treatment Group	Age (yrs)	Screening BW (kg)	Day 56 BW (kg)		Screening HR	Day 56 HR		Screening RR	Day 56 RR		Screening BP	Day 56 BP	
Cat 1	HCM	9.99		5.7			238						WNL	
Cat 2	HCM	9.97		7.5			245						WNL	
Cat 3	HCM	11.11		4.61			172						WNL	
Cat 4	High-Dose	1.92	6.39	6.74		132	190		20	50		86	126	
Cat 5	High-Dose	1.87	4.96	5.04	0.25	168	180	1	20	34	0.75	99	102	0.25
Cat 6	High-Dose	1.47	2.7	2.89		216	130		48	32		121	128	
Cat 7	Low-Dose	3.39	5.34	5.64		208	170		28	36		120	111	
Cat 8	Low-Dose	1.47	5.15	5.46	0.25	208	150	0.25	56	40	0.75	97	112	1
Cat 9	Low-Dose	1.67	2.78	2.93		168	150		48	40		95	95	
Pooled Statistics		1.77(1.47-3.39)	5.06(2.70-6.39)	5.25(2.89-6.74)	0.03*	188 (132-216)	160(130-190)	0.34	38(20-56)	38(32-50)	1	98(86-121)	112(95-128)	0.31

Supplemental Table S3. Age and physical exam findings. Descriptive statistics for age and pertinent physical exam variables are provided for all cats used in this study at available time points. For the HCM control group (Cat 1, 2, and 3) the blood pressure was deemed within normal limits as a component of a prior study through confirmation of a Doppler systolic blood pressure <160mmHg, no evidence of abnormality on fundic examination, and no evidence of renal disease via routine serum biochemical profile. Blood pressure and respiratory rate were not recorded for the purposes of this study in the HCM control group. Results from a Wilcoxon matched-pairs signed rank test between pre- (Screening) and post-treatment (Day 56) variables are presented for individual treatment groups (low- or high-dose) as well as for pooled treatment groups (low- + high-dose); only BW was statistically significant between timepoints in the pooled comparison. *$P<0.05$.

Abbreviations: yrs = years, BW = bodyweight, kg = kilogram, HR = heart rate, RR = respiration rate, BP = blood pressure, WNL = within normal limits.

Subject	**Treatment Group**	**Screening Crystals**	**Day 56 Crystals**		**Screening pH**	**Day 56 pH**		**Screening [Protein]**	**Day 56 [Protein]**		**Screening Specific Gravity**	**Day 56 Specific Gravity**		**Screening WBC**	**Day 56 WBC**	
Cat 4	High-Dose	2	0		7.5	7.0		0	1		1.048	1.041		2	2	
Cat 5	High-Dose	1	5	1	7.0	7.0	0.50	1	1	0.50	1.055	1.058	0.75	2	2	1
Cat 6	High-Dose	1	0		7	6.5		0	1		1.059	1.058		2	5	
Cat 7	Low-Dose	2	1		7.5	6.0		0	0		1.044	1.056		2	2	
Cat 8	Low-Dose	1	0	1	8.0	6.0	0.50	1	1	1	1.034	1.070	0.25	2	2	1
Cat 9	Low-Dose	0	1		6.5	6.5		1	1		1.053	1.056		2	2	
Pooled Statistics				0.78			0.13			0.50			0.31			1

Supplemental Table S4. Urinalysis findings on rapamycin-treated cats. Results from urinalysis of HCM cats receiving low- or high-dose DR rapamycin are presented. Results from a Wilcoxon matched-pairs signed rank test between pre- (Screening) and post-treatment (Day 56) variables are presented for individual treatment groups (low- or high-dose) as well as for pooled treatment groups (low- + high-dose); no statistically significant variables between timepoints were found in any of the comparisons. $^*P<0.05$.
Abbreviations: HCM = hypertrophic cardiomyopathy, DR = delayed-release, WBC= white blood cell.

Subject	Treatment Group	Screening ALT	Day 56 ALT		Screening Bicarbonate	Day 56 Bicarbonate		Screening BUN	Day 56 BUN		Screening Creatinine	Day 56 Creatinine		Screening Fructosamine	Day 56 Fructosamine		Screening Glucose	Day 56 Glucose		Screening NTproBNP	Day 56 NTproBNP		Screening SDMA	Day 56 SDMA		Screening T4	Day 56 T4	
Cat 4	High-Dose	40	60		21	20		21	20		1.3	1.1		193	209		80	94		24	55		18	13		1.9	2.2	
Cat 5	High-Dose	54	63	0.75	21	19	0.25	21	19	0.50	1.3	1.0	0.25	190	185	0.50	100	84	0.75	1500	1426	0.75	13	11	0.75	2.0	2.4	0.25
Cat 6	High-Dose	82	67		17	15		23	23		1.1	1.0		184	191		68	97		1070	1500		9	11		2.5	2.6	
Cat 7	Low-Dose	36	33		18	17		19	19		1.1	0.9		182	169		94	88		124	106		14	12		2.9	1.9	
Cat 8	Low-Dose	35	55	0.50	18	17	0.25	18	22	1	1.0	0.9	0.50	219	174	0.25	103	112	0.50	774	1353	0.50	5	9	1	1.4	1.0	0.25
Cat 9	Low-Dose	62	82		15	14		26	20		1.3	1.3		199	184		97	158		123	179		16	12		2.0	1.8	
Pooled Statistics		47(35-82)	61.5(33-82)	0.19	18(15-21)	17(14-20)	0.03*	21(18-26)	20(19-23)	0.63	1.2(1-1.3)	1(0.9-1.3)	0.06	191.5(182-219)	184.5(169-209)	0.56	95.5(68-103)	95.5(84-158)	0.31	449(24-1500)	766(55-1500)	0.31	13.5(5-18)	11.5(9-13)	0.53	2(1.4-2.9)	2.05(1-2.6)	0.72

Supplemental Table S5. Biochemistry findings on rapamycin-treated cats. Results from pertinent biochemistry measures of HCM cats receiving low- or high-dose DR rapamycin are presented. Results from a Wilcoxon matched-pairs signed rank test between pre- (Screening) and post-treatment (Day 56) variables are presented for individual treatment groups (low- or high-dose) as well as for pooled treatment groups (low- + high-dose); only bicarbonate/total CO_2 measures were statistically significant between timepoints in the pooled comparison. *P<0.05.

Abbreviations: HCM = hypertrophic cardiomyopathy, DR = delayed-release, ALT = alanine transaminase, BUN = blood urea nitrogen, NTproBNP = N-terminal prohormone of brain natriuretic peptide, SDMA = symmetric dimethylarginine, T4 = total thyroxine.

Subject	Treatment Group	Screening [EOS]	Day 56 [EOS]		Screening HCT(%)	Day 56 HCT(%)		Screening [HGB]	Day 56 [HGB]		Screening [LYMPH]	Day 56 [LYMPH]		Screening MCH	Day 56 MCH	
Cat 4	High-Dose	800	959		30.0	44.2		9.7	15.2		1950	3128		14.3	14.6	
Cat 5	High-Dose	686	393	0.50	30.3	41.1	0.25	10.5	13.7	0.25	5684	6747	1	14.1	13.9	0.75
Cat 6	High-Dose	750	323		30	39.5		9.9	13.8		3450	1856		14.1	13.4	
Cat 7	Low-Dose	258	148		44.0	29.7		13.7	10.4		2387	1685		14.6	14.2	
Cat 8	Low-Dose	283	441	1	50.0	33.2	0.25	15.7	11.8	0.25	2707	2608	0.75	13.5	13.5	0.50
Cat 9	Low-Dose	476	356		45.0	41.1		14.1	13.6		1768	2261		14.5	13.9	
Pooled Statistics		581(258-800)	374.5(148-959)	0.56	37.15(30-50)	40.3(29.7-44.2)	0.84	12.1(9.7-15.7)	13.65(10.4-15.2)	0.72	2547(1768-5684)	2435(1685-6747)	1	14.2(13.5-14.6)	13.9(13.4-14.6)	0.19

Subject	Treatment Group	Screening MCV	Day 56 MCV		Screening [MONO]	Day 56 [MONO]		Screening [NEUT]	Day 56 [NEUT]		Screening [Platelet]	Day 56 [Platelet]		Screening [RBC]	Day 56 [RBC]		Screening [WBC]	Day 56 [WBC]	
Cat 4	High-Dose	44	42		50	140		2200	3565		359	141		6.76	10.42		5.0	7.8	
Cat 5	High-Dose	41	42	0.50	196	288	0.50	3234	5672	0.25	252	232	1	7.43	9.86	0.25	9.8	13.1	0.25
Cat 6	High-Dose	43	39		300	277		3000	5244		33	328		7.04	10.27		7.5	7.7	
Cat 7	Low-Dose	47	41		170	159		3978	3307		358	211		9.36	7.33		6.8	5.3	
Cat 8	Low-Dose	43	38	0.25	707	290	0.75	16503	9261	0.75	139	91	0.75	11.62	8.76	0.50	20.2	12.6	0.75
Cat 9	Low-Dose	46	42		136	249		4352	6034		27	151		9.70	9.75		6.8	8.9	
Pooled Statistics		43.5(41-47)	41.5(38-42)	0.06	183(50-707)	263(140-290)	0.84	3606(2200-16503)	5458(3307-9261)	0.56	195.5(27-359)	181(91-328)	0.84	8.395(6.76-11.62)	9.805(7.33-10.42)	0.44	7.15(5-20.2)	8.35(5.3-13.1)	0.69

Supplemental Table S6. Hematology findings on rapamycin-treated cats. Results from pertinent hematology measures of HCM cats receiving low- or high-dose DR rapamycin are presented. Results from a Wilcoxon matched-pairs signed rank test between pre- (Screening) and post-treatment (Day 56) variables are presented for individual treatment groups (low- or high-dose) as well as for pooled treatment groups (low- + high-dose); no statistically significant variables between timepoints were found in any of the comparisons. *$P<0.05$.

Abbreviations: HCM = hypertrophic cardiomyopathy, DR = delayed-release, [EOS] = eosinophil concentration, HCT = hematocrit, [HGB] = hemoglobin concentration, [LYMPH] = lymphocyte concentration, MCH = mean corpuscular hemoglobin, MCV = mean corpuscular volume, [MONO] = monocyte concentration, [NEUT] = neutrophil concentration, [RBC] = red blood cell concentration, [WBC] = white blood cell concentration.

Subjects	**Treatment Group**	**LVEF**	**MD**	**CH**	**IF**	**CVR**	**CVN**	**NSPC**
Cat 1	HCM	1	1	1	1	1	1	11
Cat 2	HCM	2	2	1	0	3	0	9
Cat 3	HCM	2	1	1	1	1	1	6
Cat 4	High-Dose	3	1	1	0	0	1	5
Cat 5	High-Dose	2	3	2	0	1	1	4
Cat 6	High-Dose	3	2	2	1	1	1	5
Cat 7	Low-Dose	2	1	1	0	1	1	6
Cat 8	Low-Dose	3	2	3	2	1	1	3
Cat 9	Low-Dose	2	1	2	1	1	1	6
P-value		0.36	0.68	0.36	0.87	0.75	1	0.11

Supplemental Table S7. Histopathologic findings for treatment and HCM control cats. Histopathologic scores from HCM control and DR-rapamycin-treated cats are presented. Results from a Kruskal-Wallis test followed by Dunn's multiple comparisons correction are reported; no statistically significant variables between and within groups were identified. $^*P<0.05$.

Abbreviations: HCM = hypertrophic cardiomyopathy, DR = delayed release, LVEF = left ventricular endocardial fibrosis, MD = myocardial disarray, CH = cellular hypertrophy, IF = interstitial fibrosis, CVR = coronary vessel remodeling, CVN = coronary vessel narrowing, NSPC = number of perimysial collage.

Subject	Treatment Group	Screening RPSA LA/Ao	Day 56 RPSA LA/Ao		Screening RPLA LA	Day 56 RPLA LA		Screening IVSd Max	Day 56 IVSd Max		Screening LVPWDd Max	Day 56 LVPWDd Max		Screening LVIDd Sx	Day 56 LVIDd Sx	
Cat 1	HCM		1.48			1.43			6.46			5.57			1.42	
Cat 2	HCM		1.18			1.25			7.10			6.17			1.29	
Cat 3	HCM		1.31			1.46			6.55			5.70			1.34	
Cat 4	High-Dose	1.87	1.5	1	1.59	1.43	1	6.93	7.18	0.5	6.49	5.2	1	1.47	1.73	0.8
Cat 5	High-Dose	1.34	1.58		1.31	1.69		6.04	6.92		7.71	8.24		1.27	1.3	
Cat 6	High-Dose	1.36	1.52		1.31	1.06		7.11	9.97		6.41	7.24		1.26	1.09	
Cat 7	Low-Dose	1.1	1.34	0.8	1.28	1.08	0.8	6.67	6.61	0.8	4.42	4.7	0.8	1.43	1.28	0.5
Cat 8	Low-Dose	1.42	1.44		1.34	1.94		8.54	7.3		8.13	7.85		1.5	1.26	
Cat 9	Low-Dose	1.59	1.41		1.22	1.24		4.74	5.71		6.05	5.04		1.01	1.08	
Pooled Statistics		1.45(1.1-1.87)	1.47(1.34-1.58)	0.8	1.34(1.22-1.59)	1.41(1.06-1.94)	0.8	6.67(4.74-8.54)	7.28(5.71-9.97)	0.8	6.54(4.42-8.13)	6.38(4.7-8.24)	0.7	1.32(1.01-1.5)	1.29(1.08-1.73)	0.8

Subject	Screening LVIDs Sx	Day 56 LVIDs Sx		Screening FS%	Day 56 FS%		Screening LVOT maxPG	Day 56 LVOT maxPG		Screening LAF	Day 56 LAF		Screening MWT	Day 56 MWT	
Cat 1		0.40			71.5			5.10			57.0			6.46	
Cat 2		0.33			74.6			7.73			31.59			7.10	
Cat 3		0.73			45.2			1.59			70.2			6.55	
Cat 4	0.72	0.91	0.5	51	47.4	0.3	2.36	2.41	0.3	56.07	52.66	0.8	6.93	7.18	0.3
Cat 5	0.42	0.56		67	56.9		2.33	3.22		42.2	70.58		7.71	8.24	
Cat 6	0.43	0.41		65.5	62		3.08	3.55		74.55	38.82		7.11	7.24	
Cat 7	0.52	0.69	1	63.4	46.2	1	2.8	1.28	0.3	83.38	37.98	0.5	6.67	6.61	0.3
Cat 8	0.47	0.33		68.3	73.7		4.5	4.23		43.23	58.67		8.54	7.85	
Cat 9	0.49	0.48		50.8	55.8		3.19	0.75		75.44	34.59		6.05	5.71	
Pooled Statistics	0.51(0.42-0.72)	0.56(0.33-0.91)	0.5	61(50.8-68.3)	57(46.2-73.7)	0.6	3.04(2.33-4.5)	2.57(0.75-4.23)	0.7	62.48(42.2-83.38)	48.88(34.59-70.58)	0.3	7.17(6.05-8.54)	7.14(5.71-8.24)	1

Supplemental Table S8. Echocardiographic findings for treatment cats. Results from echocardiographic measures of HCM cats receiving low- or high-dose DR rapamycin are presented. Results from a Wilcoxon matched-pairs signed rank test between pre- (Screening) and post-treatment (Day 56) variables are presented for individual treatment groups (low- or high-dose) as well as for pooled treatment groups (low- + high-dose); no statistically significant variables between timepoints were found in any of the comparisons. Raw values from the control HCM group are provided; descriptive statistics and statistical comparisons for the control group were not performed as no Day 56 is available.

Abbreviations: RPSA = right parasternal short-axis, RPLA = right parasternal long-axis, LA/Ao = left atrial to aortic root diameter, LA = left atrial diameter (cm), IVSd = interventricular septum diameter in diastole (mm), LVPWDd Max = maximum left ventricular posterior wall diameter in diastole (mm), LVIDd = left ventricular internal diameter in diastole (cm), LVIDs = left ventricular internal diameter in systole (cm), Sx = short-axis, FS% = percent fractional shortening, LVOT maxPG = left ventricular outflow tract maximum pressure gradient (mmHg), LAF = left auricular flow velocity (cm/s), MWT = maximal wall thickness (mm).

Comparison	**Downregulated**	**Upregulated**
Low-Dose* vs HCM	*ECRG4, P2RX1, TAAR1*<br>(n=3)	*DCLK1, KERA, MYL1, VTN*<br>(n=4)
High-Dose* vs HCM	*AS3MT, FAM177B, HSD17B8, PPDPFL*<br>(n=4)	*CNGA1, FREM1, GRAP2, ITGB8, KIT*<br>(n=5)
All Doses* vs HCM	-<br>(n=0)	*FBLN5, FREM1, ITGB8*<br>(n=3)
Low-* vs High-Dose	-<br>(n=0)	*DIPK1A*<br>(n=1)

Supplemental Table S9. Shared down- and upregulated DEGs between LV and IVS tissues. Total number of shared DEGs between LV and IVS tissues are presented for each group comparison. *Down-/upregulation was assigned respective to the asterisk-marked groups in the comparison column.

Abbreviations: HCM = hypertrophic cardiomyopathy, DEG = differentially expressed gene(s), LV = left ventricle, IVS = interventricular septum.

	LV		IVS	
Comparison	**Underabundant**	**Overabundant**	**Underabundant**	**Overabundant**
Low-Dose* vs HCM	AARSD1, **ACOT4**, **AFM**, AK1, ALDH1A1, APBB1, **APOA1**, APOA1, **APOA4**, **AS3MT**, C3, C3, **C4BPA**, C4BPA, **C5**, CALCRL, CCS, **CCSER1**, CDV3, CES2, CES5A, CKB, **CKM**, CLPP, **CLU**, CNN1, COL28A1, COQ10B, COQ3, **CP**, CPN2, CRP, **CTSD**, CUTA, DDO, DNAJA1, EMILIN2, EPHX2, **F13A1**, F2, FAHD1, **FBXO6**, FGA, FGB, FGG, FUOM, GALM, **GPD1**, GPLD1, GPR108, **GPT**, **GSTK1**, GSTP1, GSTP1, HECTD1, **HP**, **HSPBP1**, HYI, **IGHM**, IGHM, **IGLL5**, IGLL5, IGLL5, **ITIH2**, **JCHAIN**, **KNG1**, **LDH**, LOC101089505, LOC101089505, **LOC111558267**, MCEE, **MDH1**, MDH1, **ME3**, MIA3, MMAB, MRPL12, MSRB3, MYBPC3, NCOR2, NIF3L1, NOL3, NUDCD2, OSBPL6, PDIA2, **PHOSPHO1**, PHYKPL, PITPNB, **PON1**, **PPT1**, PRG3, **PSAP**, PYURF, RAB3IL1, **S100A1**, **SDHAF2**, **SERPINB2**, **SERPIND1**, SERPING1, SHC1, SLC43A1, SYNRG, TKFC, **TMEM161A**, TRAPPC6B, TSEN15<br>(n=106)<br>**(n=37)**	**ABCA8**, **ABCC9**, **ACOT8**, ACSF3, **ACTR1B**, ADCK1, **AGK**, **AGPAT5**, AHCYL2, **AIMP1**, **ALPK3**, AMACR, **ANKRD1**, **ANKRD2**, **ANKS4B**, AP1B1, **AP1G1**, APEX1, **AQP1**, **ARPC5**, **ASB15**, ASPN, ATP2B4, **ATP2C1**, **ATP6V0A1**, **BAG6**, **BANF1**, **BCKDK**, BDH1, **BPHL**, CACNA2D1, **CAMK2A**, CAMK2A, **CANX**, CAPG, CAV3, **CAVIN4**, CCDC141, CCZ1, **CD34**, **CD44**, **CD59**, **CD9**, **CKAP4**, **CLPX**, CMAS, **COBL**, **COL15A1**, **COL18A1**, **COPB1**, **COX17**, **CYB5R3**, DAD1, **DAG1**, **DCAKD**, DDRGK1, DDX3X, DHX36, DHX9, **DMRTC2**, **DNAJB4**, DNAJB6, **DNAJC10**, **DNAJC11**, **DPYSL3**, **DSC2**, DSE, **DSG2**, **DST**, **DYNC1I2**, **EEA1**, **EFHD2**, **EFTUD2**, EHD4, EIF3B, EIF3H, EIF3I, **EIF4A1**, EIF4A3, **ELAVL1**, **ELOA**, EMILIN1, **ENAH**, EPB41, **EPB41L2**, EPB41L3, EPN1, ESYT1, **FAH**, **FAM114A1**, **FAM210A**, **FARSB**, **FBLN5**, FBXO42, **FHL1**, **FHOD3**, **FLII**, **FLNC**, **FLOT1**, **FXR2**, G3BP1, **GNB4**, GNPAT, GPHN, **H1-0**, **H1-4**, **H2AX**, **H2B**, **H3**, **H3F3A**, HACD1, **HDAC2**, **HNRNPA3**, **HNRNPL**, **HNRNPR**, **HNRNPUL2**, HP1BP3, HRAS, **HRC**, **HSPB8**, **HSPG2**, **HYOU1**, **IDH3B**, INPP5A, ITIH3, ITM2B, **KDSR**, **KPNA6**, KRT6, **KTN1**, **LAMA2**, **LAMA4**, LAMA5, LAMB1, LIMS1, LIPA, LMNA, **LMNB1**, **LMO7**, **LNPEP**, LOC101083645, LRP1, **LRRC39**, **LRRC47**, LUC7L3, **LYPLA1**, **MACF1**, MACROH2A1, **MAP7D1**, MAPT, **MARS1**, **MCAM**, **METTL7A**, MFAP4, MLF1, **MPV17**, **MRPL39**, MSN, **MTFR1L**, MTX1, **MVP**, **MYADM**, MYL1, **MYL4**, MYO18A, MYO1B, MYOM1, **NARS1**, **NDEL1**, NDRG1, **NDUFA7**, NES, **NEXN**, **NID2**, **NONO**, **NRAP**, **NUCB2**, NUDT21, **NUMA1**, **OMA1**, **PACSIN3**, PALLD, **PALM2AKAP2**, **PARP1**, **PARVA**, PDK3, **PDLIM3**, **PGRMC2**, **PHLDB2**, PLGRKT, **PLN**, **POSTN**, PPM1B, PPM1K, **PPP2R5A**, PPP2R5C, PPP2R5D, **PRKCSH**, **PRMT9**, **PRNP**, PROB1, **PRPF8**, **PTGFRN**, PTGIS, **PTP4A2**, **PXN**, RAB4, **RAP2C**, RBM39, **RBMXL1**, **RNF213**, **RPL10A**, **RPL13**, **RPL13A**, **RPL14**, **RPL15**, **RPL17**, **RPL22**, **RPL23**, **RPL24**, **RPL27A**, RPL30, **RPL32**, **RPL4**, **RPL8**, **RPS15**, **RPS18**, **RPS21**, **RPS23**, **RPS24**, **RPS25**, **RPS6**, RPS6KA3, **RRAD**, **RRBP1**, RRM2B, **RUVBL2**, **S100A10**, **SDCBP**, **SEMA3C**, **SERPINF1**, **SERPINH1**, **SF3B1**, SGCD, SGTA, **SIRT2**, **SLC2A1**, SLC44A2, SLIRP, **SMIM20**, SNRPC, **SNRPD1**, SNRPE, **SORBS1**, **SORBS2**, **SPG7**, SPNS1, **SRPRA**, SRPRB, **SRSF1**, SSR4, STAT3, **STBD1**, STXBP1, **SUCLG2**, **SUN2**, **SYNPO**, **SYNPO2L**, **TGFBI**, **THBS4**, **TIMM29**, **TLN2**, **TMED10**, **TMED2**, TMPO, TMX4, **TNXB**, **TOMM20**, TOMM22, **TRIM54**, TSPAN9, **TTN**, TUBB6, TWF2, **TXNDC5**, **U2AF1**, USP15, **VCAN**, VWA8, WFS1, **XIRP1**, **XIRP2**, ZC3H15<br>(n=293)<br>**(n=193)**	ABCC4, ACOT4, AFM, ALDH1A1, ALDH3A2, APOA1, APOA1, APOA4, **AS3MT**, ATPAF1, C3, C5, CES2, CES5A, CLPTM1, **CLU**, COL28A1, CP, CPN2, CRP, **CTSD**, CTSZ, ENO2, EPHX2, F13A1, **FUOM**, GAPDHS, GLS, GNPDA1, **GPD1L**, GSTK1, GSTP1, GSTP1, **HBS1L**, HP, HYI, IGHM, **IGLL5**, IGLL5, IMPA1, ITGB1BP2, **ITIH2**, JCHAIN, KHK, **KNG1**, LDHA, LYRM7, MAOA, MDH1, MDH1, MIA3, MT-ND1, **MYBPC3**, NAPRT, NDRG2, NDUFA7, NOL3, NPEPPS, NUDT7, OR, PACS1, PEPD, PKM, PON1, **PSAP**, QDPR, **S100A1**, S100A13, **SCAPER**, SERPINB1, **SERPINB2**, SERPIND1, SERPING1, SMPX, SPART, THAP4, TPMT, TUB<br>(n=78)<br>**(n=14)**	ABCC9, ABCF1, ACIN1, ACTG1, AFDN, AGK, AGRN, ANKRD2, **ANP32E**, **ANXA7**, AP1G1, **AP2M1**, AP3D1, **APEX1**, **ARF5**, **ARVCF**, ATP1A4, ATP2B4, ATXN10, **BANF1**, BCKDHA, BCKDK, BDH1, BPHL, BTF3, C8G, CALM, CAMK2A, CAMK2A, **CAMK2B**, CAVIN4, CD38, CD59, **CD81**, CD9, **CHCHD2**, CHMP2A, CKAP5, **CKM**, **CLPX**, CMPK1, CNBP, **COBL**, **COL15A1**, COL4A1, COL4A2, COL6A2, COL6A3, COMMD9, COMTD1, **CPSF6**, CRYL1, **CSNK2B**, **CSPG4**, CTNNA1, **CTNNA2**, CTNND1, **CTPS1**, CUL1, CUL4A, DCAF8, **DDRGK1**, DNAJB4, DNAJC11, DNAJC3, DNAJC8, DPYSL3, **DYNC1LI2**, ECHDC3, EFEMP1, EIF3H, EIF3I, EIF3K, EIF4A3, ENAH, ENDOG, **EPB41L3**, **ERP29**, FAAH, FABP5, FAF2, FAM136A, FASN, **FBLIM1**, FBLN5, FBP1, **FHL1**, **FLII**, FLNC, FXYD1, GMFB, GNB1, GOLT1A, GRB2, **GTF2I**, H1-0, **H1-10**, **H1-2**, **H1-4**, H2A, H2AX, **H2B**, **H3F3A**, HDDC3, **HMGB1**, **HNRNPA1**, **HNRBNPA3**, HNRNPAB, HNRNPDL, HNRNPR, **HNRNPU**, HNRNPUL1, **HNRNPUL2**, HOMER1, **HP1BP3**, **HSPB3**, HSPB7, **HSPG2**, **HYOU1**, **IDH3B**, **ILF2**, IPO11, IPO13, **IST1**, ITGA7, **ITGB1**, ITIH3, **JPH2**, **KANK2**, **KIF13B**, **KPNA1**, LAMA2, LAMA4, LAMA5, LAMB1, LAMC1, LASP1, LBP, LDB3, **LIMS1**, LIPA, LMAN1, **LMNB1**, LMNB2, LMO7, LOC101083645, LOC101098145, **LOC102899273**, LOC102899673, **LOC115945193**, LPIN2, LPP, **MACF1**, **MACROH2A1**, MAPRE1, **MAPT**, MECP2, **METTL7A**, MFAP5, MLF1, MLIP, MOCS1, MRPL4, MRPL49, MRPS23, MRPS9, MSI2, MT-CO1, MTARC2, **MTFR1L**, **MVP**, **MYADM**, **MYO18A**, **MYO1B**, MYO1D, **NDEL1**, NDFIP1, NDRG1, **NDUFS5**, NDUFV3, **NEXN**, NID2, **NONO**, NPEPL1, **NRAP**, NUDT21, **NUP155**, NUP205, NXN, **OBSL1**, PALLD, PARP1, **PARVA**, PDLIM1, PEX19, **PI4KA**, PLP1, POPDC2, **PPIB**, PPM1K, PPP1R12A, PPP2R5A, **PPP6R2**, PSMD4, **PTBP1**, **PTGFRN**, **PXN**, RAB21, **RAB35**, RAP2C, RBMXL1, **RHOA**, **RPL10**, RPL10A, RPL13, **RPL14**, **RPL15**, RPL18, RPL19, **RPL22**, RPL23, RPL23A, **RPL24**, RPL27, RPL27A, **RPL30**, RPL31, RPL38, RPL39, RPL4, RPL6, RPL7, **RPL7A**, **RPS11**, **RPS12**, RPS12, RPS13, RPS15A, RPS18, RPS21, **RPS23**, **RPS24**, **RPS28**, **RPS3A**, RPS5, **RPS6**, RPS6KA3, RPS7, RPS8, **RRAD**, RRAGC, S100A11, SBSPON, SCN7A, **SERBP1**, SLC25A22, **SLC25A3**, SLC2A1, SNAP23, **SNRNP200**, SNRNP70, SNRPA1, **SNRPD1**, SNRPE, **SORBS1**, SORBS2, SORT1, **SRI**, **SRSF1**, SSPN, ST13, **STRN3**, SUGCT, **SUN2**, **SVIL**, **SYNCRIP**, SYNPO, **SYNPO2L**, **TBRG4**, TEKT3, TGFBI, **TIMMDC1**, TINAGL1, TLN2, TMEM120A, TMEM223, **TNPO1**, TOMM20, TRA2B, **TRIM54**, TRIM63, TUBB, **U2AF1**, **UBE2G2**, UBE3C, UPF1, **USP7**, VNN1, VTA1, XIRP1, XIRP2, YBX1, **YBX3**, YES1<br>(n=304)<br>**(n=113)**
High-Dose* vs HCM	ACAA2, ACADS, **ACOT4**, **AFM**, AFP, ALDOC, **APOA1**, APOA1, **APOA4**, APOB, **AS3MT**, ATPAF1, BST1, **C4BPA**, **C5**, CA2, **CCSER1**, CD1H11orf54, **CKM**, CLPTM1, **CLU**, **CP**, CPT2, **CTSD**, DHDH, **F13A1**, FASN, FBP2, **FBXO6**, FHL2, GALE, GCLM, **GPD1**, GPD1L, **GPT**, GST, **GSTK1**, GSTO1, GYPA, HIBCH, **HP**, **HSPBP1**, IGFALS, **IGHM**, IGHM, **IGLL5**, IGLL5, IGLL5, ITIH1, **ITIH2**, **JCHAIN**, KIF12, **KNG1**, **LDH**, LDHD, LGALS3BP, **LOC111558267**, **MDH1**, MDH1, **ME3**, MRPS2, NCEH1, NIT2, NNMT, PGPEP1, **PHOSPHO1**, PHYH, **PON1**, **PPT1**, **PSAP**, PYCARD, RAC3, **S100A1**, **SDHAF2**, **SERPINB2**, **SERPIND1**, STK39, TATDN1, TF, **TMEM161A**, TMX3, TPM2, TRAF5, TRIM72, UAP1, ZBED8<br>(n=86)<br>**(n=37)**	**ABCA8**, **ABCC9**, ABCD1, ABLIM1, ACBD3, **ACOT8**, ACSL3, **ACTR1B**, ADSL, **AGK**, **AGPAT5**, AGRN, AHCYL, **AIMP1**, ALDH, ALDH18A1, **ALPK3**, **ANKRD1**, **ANKRD2**, **ANKS4B**, ANXA7, **AP1G1**, **AQP1**, ARHGAP10, ARMC10, ARMCX3, ARPC1A, ARPC2, ARPC3, **ARPC5**, **ASB15**, **ATP2C1**, **ATP6V0A1**, B2M, BAG3, **BAG6**, **BANF1**, **BCKDK**, **BPHL**, BTF3L4, **CAMK2A**, **CANX**, CAP2, CAST, CAVIN3, **CAVIN4**, CBX1, CC2D1A, CCDC47, **CD34**, CD38, **CD44**, **CD59**, CD81, **CD9**, CDK5RAP3, CHRD, **CKAP4**, **CLPX**, CLTB, CLTCL1, **COBL**, **COL15A1**, **COL18A1**, COL4A1, COMMD4, COMTD1, **COPB1**, COPG1, CORO1C, **COX17**, CRYAB, CTNNA1, CTNNA2, CTNND1, CYB5A, **CYB5R3**, **DAG1**, **DCAKD**, DCTN1, DCTN2, DES, **DMRTC2**, DNAJA1, **DNAJB4**, **DNAJC10**, **DNAJC11**, **DPYSL3**, **DSC2**, **DSG2**, **DST**, **DYNC1I2**, DYNC1LI2, ECHDC3, **EEA1**, EFHD1, **EFHD2**, **EFTUD2**, EIF3D, EIF3F, EIF3G, **EIF4A1**, **ELAVL1**, **ELOA**, **ENAH**, ENDOG, **EPB41L2**, ERP29, EXOSC7, FAF2, **FAH**, **FAM114A1**, **FAM210A**, **FARSB**, **FBLN5**, **FHL1**, **FHOD3**, FIS1, **FLII**, **FLNC**, **FLOT1**, FUNDC2, FUS, **FXR2**, GNB1, **GNB4**, GNG12, GRSF1, GTF2I, **H1-0**, H1-10, **H1-4**, H2A, **H2AX**, **H2B**, **H3**, **H3F3A**, H4, HBA1, **HDAC2**, HNRNPA1, **HNRNPA3**, HNRNPAB, HNRNPF, **HNRNPL**, HNRNPM, **HNRNPR**, **HNRNPUL2**, **HRC**, HSP90B1, HSPA2, HSPA5, HSPB3, **HSPB8**, **HSPG2**, **HYOU1**, IARS1, **IDH3B**, ILF2, ITGA7, **KDSR**, KHDRBS1, KIF13B, **KPNA6**, KRT19, **KTN1**, **LAMA2**, **LAMA4**, LAMB2, LAMTOR1, LASP1, LDB3, LGALS3, LMATOR3, **LMNB1**, **LMO7**, **LNPEP**, LPGAT1, LPP, **LRRC39**, **LRRC47**, **LYPLA1**, **MACF1**, MAP1B, **MAP7D1**, **MARS1**, **MCAM**, **METTL7A**, MFF, MIX23, MLIP, **MPV17**, **MRPL39**, MRPS34, MSI2, **MTFR1L**, **MVP**, **MYADM**, **MYL4**, **NARS1**, **NDEL1**, **NDUFA7**, NDUFAF2, NDUFV3, **NEXN**, NIBAN1, **NID2**, **NONO**, NPEPL1, NPM1, **NRAP**, **NUCB2**, **NUMA1**, **OMA1**, OSTF1, **PACSIN3**, **PALM2AKAP2**, **PARP1**, **PARVA**, PCYOX1, PDLIM1, **PDLIM3**, PFN2, **PGRMC2**, **PHLDB2**, PLEC, **PLN**, PNPLA6, POGLUT3, **POSTN**, PPIB, **PPP2R5A**, **PRKCSH**, **PRMT9**, **PRNP**, PRODH, **PRPF8**, PSMC3, **PTGFRN**, PTGS1, **PTP4A2**, **PXN**, RAB21, RAB31, RAB5C, RAB7A, RANBP1, **RAP2C**, **RBMXL1**, RCC1, **RNF213**, RPL10, **RPL10A**, RPL12, **RPL13**, **RPL13A**, **RPL14**, **RPL15**, **RPL17**, **RPL22**, **RPL23**, **RPL24**, RPL26L1, RPL27, **RPL27A**, RPL28, **RPL32**, RPL35A, **RPL4**, RPL7, RPL7A, **RPL8**, RPL9, RPS11, RPS12, RPS13, **RPS15**, RPS15A, **RPS18**, RPS18, **RPS21**, **RPS23**, **RPS24**, **RPS25**, RPS27A, RPS28, RPS3, RPS3A, **RPS6**, RPS7, RPS8, **RRAD**, **RRBP1**, RTN4, **RUVBL2**, **S100A10**, SAE1, SAFB, SAR1A, **SDCBP**, SEC22B, **SEMA3C**, SEPTIN9, SERBP1, **SERPINF1**, **SERPINH1**, **SF3B1**, SFPQ, **SIRT2**, **SLC2A1**, **SMIM20**, SNRNP70, **SNRPD1**, **SORBS1**, SORBS1, **SORBS2**, **SPG7**, SRI, **SRPRA**, **SRSF1**, ST13, **STBD1**, STYXL2, SUB1, **SUCLG2**, **SUN2**, SURF4, SVIL, SYNCRIP, **SYNPO**, **SYNPO2L**, TBCA, **TGFBI**, TGM2, **THBS4**, **TIMM29**, **TLN2**, **TMED10**, **TMED2**, TMEM11, TNKS1BP1, **TNXB**, **TOMM20**, **TRIM54**, TRIM63, TRIP6, TSTD1, **TTN**, **TXNDC5**, **U2AF1**, UBE2L3, UPF1, USP28, VAPA, VASP, VAT1, **VCAN**, VPS26A, WASF3, **XIRP1**, **XIRP2**, YBX1, ZYX<br>(n=363)<br>**(n=193)**	ACADVL, ACOX3, **AS3MT**, CA3, **CLU**, CPT2, **CTSD**, DPYS, FABP3, **FUOM**, **GPD1L**, GSTO1, GUSB, **HBS1L**, IGLL5, **IGLL5**, **ITIH2**, **KNG1**, **MYBPC3**, NCEH1, **PSAP**, **S100A1**, **SCAPER**, **SERPINB2**, SETD7, SLC43A1, UCHL1<br>(n=27)<br>**(n=14)**	ADSL, ALYREF, ANKRD1, **ANP32E**, **ANXA7**, **AP2M1**, **APEX1**, API5, **ARF5**, ARPC2, **ARVCF**, ATG7, ATOX1, **BANF1**, BCAS2, BZW1, CA14, **CAMK2B**, CASP3, **CD81**, CDIPT, CELF1, CERS1, **CHCHD2**, CKAP4, **CKM**, **CLPX**, **COBL**, COL14A1, **COL15A1**, COL18A1, COPB1, COPB2, COPG1, **CPSF6**, **CSNK2B**, **CSPG4**, **CTNNA2**, **CTPS1**, DAG1, **DDRGK1**, DHX9, DNAJA1, DSC2, DSG2, **DYNC1LI2**, EIF3B, EIF3D, EIF3J, EPB41L2, **EPB41L3**, EPB41L3, **ERP29**, FARSB, **FBLIM1**, **FHL1**, FILIP1, **FLII**, FLOT1, FYN, **GTF2I**, **H1-10**, **H1-2**, **H1-4**, **H2B**, **H3F3A**, **HMGB1**, HNMT, **HNRNPA1**, **HNRNPA3**, HNRNPF, HNRNPH3, HNRNPL, HNRNPM, **HNRNPU**, **HNRNPUL2**, **HP1BP3**, HSD17B6, **HSPB3**, **HSPG2**, **HYOU1**, **IDH3B**, **ILF2**, **IST1**, **ITGB1**, **JPH2**, **KANK2**, **KIF13B**, **KPNA1**, KRAS, LARS1, LGALS3, **LIMS1**, **LMNB1**, **LOC102899273**, **LOC115945193**, LYPLA1, **MACF1**, **MACROH2A1**, **MAPT**, MCAM, **METTL7A**, MRPL39, MRTO4, **MTFR1L**, **MVP**, **MYADM**, **MYO18A**, **MYO1B**, NARS1, NCKAP1, NCL, **NDEL1**, NDUFAF2, **NDUFS5**, **NEXN**, **NONO**, **NRAP**, **NUP155**, NUP93, **OBSL1**, **PARVA**, PCYOX1, PDXK, **PI4KA**, PLGRKT, POGLUT3, **PPIB**, PPM1F, **PPP6R2**, PPP6R3, PRKAR2B, PRPF8, **PTBP1**, **PTGFRN**, PURB, PXDN, **PXN**, PZP, **RAB35**, RAB4, RBM39, RBM8A, **RHOA**, **RPL10**, **RPL14**, **RPL15**, **RPL22**, **RPL24**, **RPL30**, **RPL7A**, **RPS11**, **RPS12**, **RPS23**, **RPS24**, **RPS28**, **RPS3A**, **RPS6**, **RRAD**, RTN3, RTN4, RUVBL2, SAE1, SARS2, SDCBP, SEC22B, SEC23A, SEC31A, SEC61A1, SEPTIN1, **SERBP1**, SH3GL1, SIDT2, **SLC25A3**, **SNRNP200**, **SNRPD1**, SNX6, **SORBS1**, SPCS2, **SRI**, **SRSF1**, SSB, STBD1, STOML2, **STRN3**, SUCLG2, **SUN2**, **SVIL**, **SYNCRIP**, **SYNPO2L**, TARDBP, TBCB, **TBRG4**, THNSL1, **TIMMDC1**, TIPRL, TMED2, **TNPO1**, TRIM28, **TRIM54**, TRIP10, **U2AF1**, **UBE2G2**, UCHL5, UFC1, **USP7**, VLDLR, VPS4B, WDR82, XPO7, **YBX3**<br>(n=211)<br>**(n=113)**
All Doses* vs HCM	AARSD1, ABI1, AFM, ALDH1A1, APOA1, APOA1, APOA4, APOB, AS3MT, ATPAF1, BST1, C4BPA, C5, CALCRL, CCSER1, CD1H11orf54, CES2, CES5A, CKM, CLPTM1, CLU, COL28A1, CP, CPN2, CRP, CTSD, DDO, DHDH, EMILIN2, F13A1, F2, FAHD1, FBXO6, FUOM, GALE, GAPDHS, GCLM, GPD1, GPD1L, GPLD1, GPT, GST, GSTK1, GSTP1, GSTP1, GYPA, HP, HSPBP1, HYI, IGFALS, IGHM, IGHM, IGLL5, IGLL5, IGLL5, ILF3, ITIH1, ITIH2, JCHAIN, KIF12, KLKB1, KNG1, LDH, LGALS3BP, LIMCH1, LOC101089505, LOC101089505, LOC111558267, MDH1, MDH1, ME3, MRPL12, MRPS2, MYBPC3, NCOR2, NNMT, NUDCD2, PDIA2, PHOSPHO1, PHYH, PITPNB, PON1, PPT1, PSAP, PSMG4, PSPC1, RPIA, S100A1, SDHAF2, SERPINB2, SERPIND1, SERPING1, SMAD, SPTBN2, STK39,	ABCA8, ABCC9, ABCD1, ABI3BP, ABLIM1, ABLIM1, ACBD3, ACOT8, ACSF3, ACTR1B, AGK, AGPAT5, AGRN, AHCYL2, AIMP1, ALPK3, ANKRD1, ANKRD2, ANKS4B, ANXA7, AP1B1, AP1G1, APEX1, AQP1, ARHGAP10, ARMC10, ARPC1A, ARPC3, ARPC5, ASB15, ASPN, ATP2B4, ATP2C1, ATP6V0A1, B2M, BAG3, BAG6, BANF1, BCKDK, BPHL, BTF3L4, CA3, CAD, CAMK2A, CAMK2A, CANX, CAPG, CAST, CAV3, CAVIN4, CBX1, CCDC141, CCDC47, CD34, CD44, CD59, CD81, CD9, CHRD, CKAP4, CLPX, CMAS, CNTN4, COBL, COL15A1, COL18A1, COL4A1, COL4A2, COL6A1, COL6A2, COL6A3, COMTD1, COPB1, COX17, CRYAB, CSPG4, CTNNA1, CTNNA2, CYB5A, CYB5R3, DAG1, DCAKD, DCTN1, DDRGK1, DDX17, DDX3X, DES, DHX36, DHX9, DMRTC2, DNAJB4, DNAJB6, DNAJC10, DNAJC11, DNAJC5, DNAJC8, DPYSL3, DSC2, DSE, DSG2, DST, DYNC1I2, DYNC1LI2, ECHDC3, EEA1, EFEMP1, EFHD1, EFHD2, EFTUD2, EIF2S1, EIF2S2, EIF3F, EIF3G, EIF3H, EIF3I, EIF4A1, EIF4B, ELAVL1, ELOA, EMILIN1, EMP1, ENAH, EPB41, EPB41L2, EPB41L3, EPN1, EXOSC7, FAH, FAM114A1, FAM210A, FAM98B, FARSB, FBLN5, FBN1, FBXO42, FHL1, FHOD3, FLII, FLNC, FLOT1, FSD2, FUNDC2, FUS, FXR2, GNB1, GNB4, GNG12, GRK, H1-0, H1-10, H1-4, H2A, H2AX, H2B, H3, H3F3A, HACD1, HBA1, HDAC2, HEBP1, HMGB1, HNRNPA1, HNRNPA3, HNRNPAB, HNRNPF, HNRNPL, HNRNPR, HNRNPUL2, HP1BP3, HRAS, HRC, HSPA2, HSPB3, HSPB8, HSPG2, HYOU1, IDH3B, ILF2, INPP5A, ITGA7, ITM2B, KDSR, KHDRBS1, KIF13B, KPNA6, KRT19, KRT6, KTN1, LAMA2, LAMA4, LAMA5, LAMB1, LAMB2, LAMTOR1, LDB3, LDB3, LGALS3, LIPA, LMATOR3, LMNB1, LMO7, LNPEP, LOC101083645, LOC101097198, LRP1, LRRC39, LRRC47, LUC7L2, LUC7L3, LYPLA1, MACF1, MACROH2A1, MAP1B, MAP7D1, MAPT, MARS1, MAT2A, MCAM, METTL26, METTL7A, MIX23, MLF1, MLIP, MOCS1, MPV17, MROH1, MRPL16, MRPL39, MSI2, MSN, MTFR1L, MTX1, MVP, MYADM, MYL1, MYL4, MYO1B, MYO1D, MYOM1, NARS1, NDEL1, NDUFA7, NDUFAF2, NDUFB3, NDUFV3, NES, NEXN, NFIA, NIBAN1, NID2, NONO, NPM1, NRAP, NT5C1A, NUCB2, NUMA1, OBSL1, OMA1, PACSIN3, PALM2AKAP2, PARP1, PARVA, PCYOX1, PDK3, PDK4, PDLIM3, PEX10, PFN2, PGRMC2, PHLDB2, PI16, PIGT, PIR, PLGRKT, PLN, PLXNA1, PNPLA6, PNPLA7, POGLUT3, POSTN, PPIB, PPM1B, PPP2R5A, PRKCSH, PRMT9, PRNP, PROB1, PRODH, PRPF8, PSMB, PTBP1, PTGFRN, PTGIS, PTGS1, PTP4A2, PTPMT1, PXN, RAB21, RAB5C, RAB7A, RANBP1, RAP2C, RBM39, RBMXL1, RBPMS2, RNF213, RO60, ROMO1, RPL10, RPL10A, RPL12, RPL13, RPL13A, RPL14, RPL15, RPL17;, RPL18, RPL22, RPL23, RPL23A, RPL24, RPL27,	ABCC4, AFM, APOA1, APOA1, APOA4, AS3MT, ATPAF1, CES5A, CLPTM1, CLU, COL28A1, CP, CTSD, DPYS, EPHX2, F13A1, FSD2, FUOM, GDF10, GPD1L, GSTK1, GSTP1, GUSB, HBS1L, HP, IGHM, IGLL5, IGLL5, ITIH2, JCHAIN, KHK, KNG1, LYRM7, MDH1, MVB12B, MYBPC3, NNMT, NUDT7, PNPO, PON1, PSAP, S100A1, SCAPER, SERPINB2, SETD7<br>(n=45)	ABCC9, ABCF1, ABLIM1, ACIN1, ACTG1, AFDN, AGK, AGRN, AKAP12, ALYREF, AMT, ANKRD1, ANKRD2, ANP32E, ANXA7, AP2A2, AP2M1, AP3D1, APEX1, ARF5, ARVCF, ATG7, ATL3, ATOX1, ATP2B4, ATXN10, ATXN2, BANF1, BCAS2, BCKDK, BPHL, C8G, CA14, CA3, CAMK2A, CAMK2A, CAMK2B, CAPG, CAPRIN1, CARS2, CAVIN4, CD38, CD44, CD59, CD81, CD9, CDV3, CELF1, CERS1, CHCHD2, CHMP2A, CKAP4, CKAP5, CKM, CLPX, CMPK1, COBL, COL15A1, COL18A1, COL1A1, COL4A1, COL4A2, COL6A1, COL6A2, COMTD1, COPB1, CORO1C, CPSF6, CRYL1, CSNK2B, CSPG4, CTNNA1, CTNNA2, CTNND1, CTPS1, CUL1, CUL4A, CYB5A, DAG1, DCAF8, DDRGK1, DHX9, DNAJA1, DNAJB4, DNAJC11, DNAJC3, DNAJC8, DPYSL3, DRG1, DSG2, DYNC1LI2, ECHDC3, EEF1A1, EFEMP1, EIF3B, EIF3D, EIF3J, EIF3K, EIF4A3, EMD, ENAH, ENDOG, ENO1, EPB41L2, EPB41L3, EPHX1, ERP29, ESYT1, ESYT2, ETFRF1, EWSR1, EXT2, FAAH, FAF2, FAH, FAM136A, FARP1, FARSB, FASN, FBLIM1, FBLN1, FBLN2, FBLN5, FBN1, FDX1, FHL1, FILIP1, FLII, FLOT1, FXYD1, G3BP2, GFER, GNB1, GNG12, GOLT1A, GORASP2, GPHN, GRB2, GTF2I, H1-0, H1-10, H1-2, H1-4, H2A, H2AX, H2B, H3F3A, HACD1, HDAC2, HDDC3, HDLBP, HECTD1, HMGB1, HNMT, HNRNPA1, HNRNPA1, HNRNPA3, HNRNPAB, HNRNPH3, HNRNPM, HNRNPR, HNRNPU, HNRNPUL1, HNRNPUL2, HOMER1, HP1BP3, HSD17B11, HSD17B6, HSPB3, HSPG2, HYOU1, IDH3B, ILF2, ILF3, INPPL1, IPO11, IPO13, IST1, ITGA7, ITGB1, ITIH3, JPH2, KANK2, KIF13B, KPNA1, KRAS, KRT10, KTN1, LAMA4, LAMA5, LAMB1, LARS1, LASP1, LBP, LDB3, LGALS3, LIMS1, LMAN1, LMAN2, LMNA, LMNB1, LMNB2, LMO7, LOC101083645, LOC102899273, LOC115945193, LPIN2, LPP, LSM3, LYPLA1, MACF1, MACROH2A1, MAP1B, MAP4, MAPRE1, MAPT, MCAM, MECP2, METTL7A, MFAP5, MLF1, MLIP, MOCS1, MRPL4, MRPL49, MRPS26, MRPS30, MRPS5, MRPS9, MRTO4, MSI2, MSN, MSRB3, MT-CO1, MTFR1L, MVP, MYADM, MYH13, MYL12A, MYO18A, MYO18B, MYO1B, MYO1D, NARS1, NCAM1, NCKAP1, NDEL1, NDRG1, NDUFAF2, NDUFB1, NDUFS5, NDUFV3, NEK7, NES, NEXN, NFIA, NIBAN1, NID2, NONO, NOP58, NRAP, NT5DC1, NUDT21, NUP155, NUP205, NUP93, NXN, OBSL1, ODR4, OMA1, PALLD, PARP1, PARVA, PCBD1, PCYOX1, PDHA1, PDLIM3, PFAS, PI4KA, PLGRKT, PLN, PLP1, PNMT, POGLUT3, POLR2H, POR, POSTN, PPIB, PPM1B, PPP1R12B, PPP2R5A, PPP6R2, PPP6R3, PRDX4, PRNP, PRPF8, PTBP1, PTGFRN, PURB, PXDN, PXN, PYCR3, PZP, QCR6, RAB11B, RAB35, RAB3IL1, RAB4, RAB5A, RAP2C, RBM39, RBM8A, RBMXL1, RHOA, RPL10, RPL10A, RPL14, RPL15, RPL18, RPL19, RPL22, RPL23A, RPL24, RPL26L1, RPL27A, RPL30, RPL31, RPL38, RPL39, RPL6, RPL7, RPL7A, RPS11, RPS12, RPS12, RPS15, RPS15A, RPS21,

	TBCB, TMEM161A, TSEN15, TWF1 (n=99)	RPL27A, RPL28, RPL30, RPL31, RPL32, RPL35A, RPL36, RPL39, RPL4, RPL7A, RPL8, RPS11, RPS12, RPS13, RPS15, RPS15A, RPS18, RPS18, RPS20, RPS21, RPS23, RPS24, RPS25, RPS27A, RPS6, RPS6KA3, RPS8, RRAD, RRBP1, RTN4, RUVBL2, S100A10, SAFB, SAR1A, SDCBP, SEC22B, SEMA3C, SEPTIN9, SERBP1, SERPINF1, SERPINH1, SF1, SF3B1, SGCD, SGTA, SH3GLB1, SIRT2, SLC2A1, SLC44A2, SLIRP, SMIM20, SNRNP70, SNRPC, SNRPD1, SNRPE, SORBS1, SORBS1, SORBS2, SORT1, SPARCL1, SPG7, SRPRA, SRSF1, SSPN, SSR4, STBD1, SUCLG2, SUN2, SVIL, SYNCRIP, SYNPO, SYNPO2L, TARS1, TBCA, TGFBI, TGM2, THBS4, TIMM29, TINAGL1, TLN2, TMED10, TMED2, TMEM11, TMX4, TNKS1BP1, TNXB, TOMM20, TOMM22, TRIM54, TRIM63, TRIP6, TSPAN9, TTN, TUBB, TUBB6, TXNDC5, U2AF1, UBE2L3, USP28, VASP, VCAN, VNN1, WASL, WDR61, WFS1, XIRP1, XIRP2, YBX1, ZMPSTE24, ZYX (n=432)		RPS23, RPS24, RPS28, RPS3A, RPS5, RPS6, RPS6KA3, RPS8, RRAD, RRAGC, RRBP1, RSF1, RTN3, RUVBL2, S100A11, SAFB, SARS2, SBSPON, SCN7A, SDCBP, SEC31A, SEC61A1, SELENOO, SEMA3C, SEPTIN1, SEPTIN8, SERBP1, SERPINC1, SF1, SFXN3, SH3GL1, SHC1, SIDT2, SLC20A2, SLC25A22, SLC25A3, SLC2A1, SLC30A9, SLC9A3R1, SMC1A, SMYD2, SNAP23, SNRNP200, SNRNP70, SNRPA1, SNRPD1, SNRPD3, SNRPE, SNX6, SOD3, SORBS1, SORBS2, SORT1, SPCS2, SRI, SRSF1, SSPN, ST13, STAT3, STBD1, STOML2, STRN3, SUGCT, SUN1, SUN2, SVIL, SYNCRIP, SYNE1, SYNPO, SYNPO2L, TARDBP, TBCB, TBRG4, TGFBI, THBD, THNSL1, TIMM29, TIMMDC1, TINAGL1, TIPRL, TLN2, TMED2, TMEM120A, TMEM201, TMPO, TNKS1BP1, TNPO1, TNPO2, TNXB, TOMM20, TOMM7, TRA2B, TRIM28, TRIM54, TRIM63, TRIP6, TUBB, TXNDC5, U2AF1, UAP1L1, UBA7, UBE2G2, UBXN1, UCHL5, UPF1, USP13, USP28, USP7, VAPB, VASP, VCAN, VDAC3, VLDLR, VNN1, VPS36, VTA1, WDR82, XIRP1, XIRP2, XPO7, YBX1, YBX3 (n=460)
Low-* vs High-Dose	- (n=0)	FBP2, USP15 (n=2)	NADPH (n=1)	AFP, PPM1K, ZBED8 (n=3)

Supplemental Table S10. Total global DAPs of LV and IVS tissues. Global LV and IVS DAPs of all study comparisons are reported. Bolded protein symbols represent shared DAPs between low-dose vs HCM and high-dose vs HCM comparisons per tissue type. All under- (log2FoldChange<-1) and overabundant (log2FoldChange>1) DAPs reported here were statistically significant (P-value$_{adjusted}$<0.05). *Under-/overabundance was assigned respective to the asterisk-marked groups in the comparison column.

Abbreviations: HCM = hypertrophic cardiomyopathy, DAP = differentially abundant peptide(s), LV = left ventricle, IVS = interventricular septum.

	Plasma		Urine	
Comparison	**Underabundant**	**Overabundant**	**Underabundant**	**Overabundant**
*Low-Dose (pre) vs Low-Dose (post)**	- (n=0)	- (n=0)	ALDOB, AQP2, ATP1B1, ATP5F1B, B3GNT2, CDH11, COL18A1, CRYZ, FES, H2B, HSPA5, IL1RAP, ISLR, LOC101091370, LOC101098453, MYL1, NIT2, NUTF2, PGK1, PODXL, PPIA, PRSS53, PRSS8, RAB7A, SLC5A10, TREML1 (n=26)	ASGR1, FSTL3, NPC1L1, PRNP, VASN (n=5)
*High-Dose (pre) vs High-Dose (post)**	- (n=0)	- (n=0)	- (n=0)	- (n=0)
*All Doses (pre) vs All Doses (post)**	ACE2, CXC, IGFBP7, LOC101080826, THBS1, TIMP2, VWF (n=7)	APOA1, APOA4, APOC4, VNN1 (n=4)	AGA, AQP2, ARSA, B3GNT2, CD300LG, CD63, CHMP2B, CILP, CRYZ, EFNB2, FABP3, FES, GALC, GBA3, H2B, HAAO, IDH1, IGFBP1, IL1RAP, IL4R, ISLR, KCNJ15, LBP, LOC101091370, LRRC19, MTA2, NEU1, NUTF2, OLFM1, PBLD, PLA2G15, PMM2, PODXL, PPIA, PPIB, RAC1, RHBG, RRAS2, SELENBP1, SHISA7, SLC25A3, SLC3A1, SLC5A10, SORT1, SPON1, TMEM123, TREML1, TTN, TUBB, TUBB4B (n=50)	AFM, ALB, ANGPTL2, ANGPTL3, ANXA6, APBB1, APOH, ASGR1, C4A, C4BPA, C4BPA, CD27, CD55, CFI, DSC1, ELANE, EPS8, FABP5, FBLN1, GC, GOLM1, GPR108, GSN, HRG, ICOSLG, ITIH4, KRT10, KRT18, KRT2, LACTB2, NPC1L1, PEAR1, PLG, PZP, SIAE, STC1, TF, VASN, WFDC2 (n=39)

Supplemental Table S11. Total global DAPs of plasma and urine samples. Global plasma and urine DAPs of all study comparisons are reported. All under- (log2FoldChange<-1) and overabundant (log2FoldChange>1) DAPs reported here were statistically significant (*P*-$value_{adjusted}<0.05$). *Under-/overabundance was assigned respective to the asterisk-marked groups in the comparison column. Abbreviations: HCM = hypertrophic cardiomyopathy, DAP = differentially abundant peptide(s).

Method	Enrichment Analysis			Specimen	Comparison	Regulation
LCMS	**GO CC**	**GO BP**	**KEGG**	LV	Low-Dose vs HCM	UA
LCMS	**GO CC**	**GO BP**	**KEGG**	LV	Low-Dose vs HCM	OA
LCMS	**GO CC**	**GO BP**	**KEGG**	LV	High-Dose vs HCM	UA
LCMS	**GO CC**	**GO BP**	**KEGG**	LV	High-Dose vs HCM	OA
LCMS	**GO CC**	**GO BP**	**KEGG**	LV	All Doses vs HCM	UA
LCMS	**GO CC**	**GO BP**	**KEGG**	LV	All Doses vs HCM	OA
LCMS	GO CC	GO BP	KEGG	LV	Low- vs High-Dose	UA
LCMS	GO CC	GO BP	KEGG	LV	Low- vs High-Dose	OA
LCMS	**GO CC**	**GO BP**	**KEGG**	IVS	Low-Dose vs HCM	UA
LCMS	**GO CC**	**GO BP**	**KEGG**	IVS	Low-Dose vs HCM	OA
LCMS	GO CC	GO BP	**KEGG**	IVS	High-Dose vs HCM	UA
LCMS	**GO CC**	**GO BP**	**KEGG**	IVS	High-Dose vs HCM	OA
LCMS	**GO CC**	**GO BP**	**KEGG**	IVS	All Doses vs HCM	UA
LCMS	**GO CC**	**GO BP**	**KEGG**	IVS	All Doses vs HCM	OA
LCMS	GO CC	GO BP	KEGG	IVS	Low- vs High-Dose	UA
LCMS	GO CC	GO BP	KEGG	IVS	Low- vs High-Dose	OA
LCMS	GO CC	GO BP	KEGG	Plasma	Low-Dose Pre vs Post	UA
LCMS	GO CC	GO BP	KEGG	Plasma	Low-Dose Pre vs Post	OA
LCMS	GO CC	GO BP	KEGG	Plasma	High-Dose Pre vs Post	UA
LCMS	GO CC	GO BP	KEGG	Plasma	High-Dose Pre vs Post	OA
LCMS	GO CC	GO BP	KEGG	Plasma	All Doses Pre vs Post	UA
LCMS	**GO CC**	**GO BP**	**KEGG**	Plasma	All Doses Pre vs Post	OA
LCMS	GO CC	GO BP	KEGG	Urine	Low-Dose Pre vs Post	UA
LCMS	**GO CC**	GO BP	KEGG	Urine	Low-Dose Pre vs Post	OA
LCMS	GO CC	GO BP	KEGG	Urine	High-Dose Pre vs Post	UA
LCMS	GO CC	GO BP	KEGG	Urine	High-Dose Pre vs Post	OA
LCMS	GO CC	GO BP	KEGG	Urine	All Doses Pre vs Post	UA

LCMS	**GO CC**	GO BP	KEGG	Urine	All Doses Pre vs Post	OA
RNA-Seq	**GO CC**	**GO BP**	**KEGG**	LV	Low-Dose vs HCM	DR
RNA-Seq	**GO CC**	**GO BP**	**KEGG**	LV	Low-Dose vs HCM	UR
RNA-Seq	**GO CC**	**GO BP**	**KEGG**	LV	High-Dose vs HCM	DR
RNA-Seq	GO CC	**GO BP**	KEGG	LV	High-Dose vs HCM	UR
RNA-Seq	GO CC	GO BP	KEGG	LV	All Doses vs HCM	DR
RNA-Seq	**GO CC**	**GO BP**	KEGG	LV	All Doses vs HCM	UR
RNA-Seq	**GO CC**	**GO BP**	**KEGG**	LV	Low- vs High-Dose	DR
RNA-Seq	**GO CC**	**GO BP**	KEGG	LV	Low- vs High-Dose	UR
RNA-Seq	GO CC	GO BP	**KEGG**	IVS	Low-Dose vs HCM	DR
RNA-Seq	**GO CC**	**GO BP**	KEGG	IVS	Low-Dose vs HCM	UR
RNA-Seq	GO CC	GO BP	KEGG	IVS	High-Dose vs HCM	DR
RNA-Seq	GO CC	**GO BP**	KEGG	IVS	High-Dose vs HCM	UR
RNA-Seq	GO CC	GO BP	KEGG	IVS	All Doses vs HCM	DR
RNA-Seq	GO CC	**GO BP**	**KEGG**	IVS	All Doses vs HCM	UR
RNA-Seq	GO CC	GO BP	KEGG	IVS	Low- vs High-Dose	DR
RNA-Seq	GO CC	GO BP	KEGG	IVS	Low- vs High-Dose	UR

Supplemental Table S12. Total GO and KEGG term analyses on proteomic and transcriptomic comparisons. All possible cellular component and biological processes GO and KEGG pathway term analyses are listed (n=132). Results from identified enriched genes/proteins for proteomic (n=39) and transcriptomic (n=23) GO and/or KEGG term analyses are bolded.

Abbreviations: LCMS = liquid mass spectrometry, GO = gene ontology, CC = cellular component, BP = biological processes, LV = left ventricle, IVS = interventricular septum, HCM = hypertrophic cardiomyopathy, UA = underabundant, OA = overabundant, DR = downregulated, UR = upregulated.

SUPPLEMENTAL FIGURES

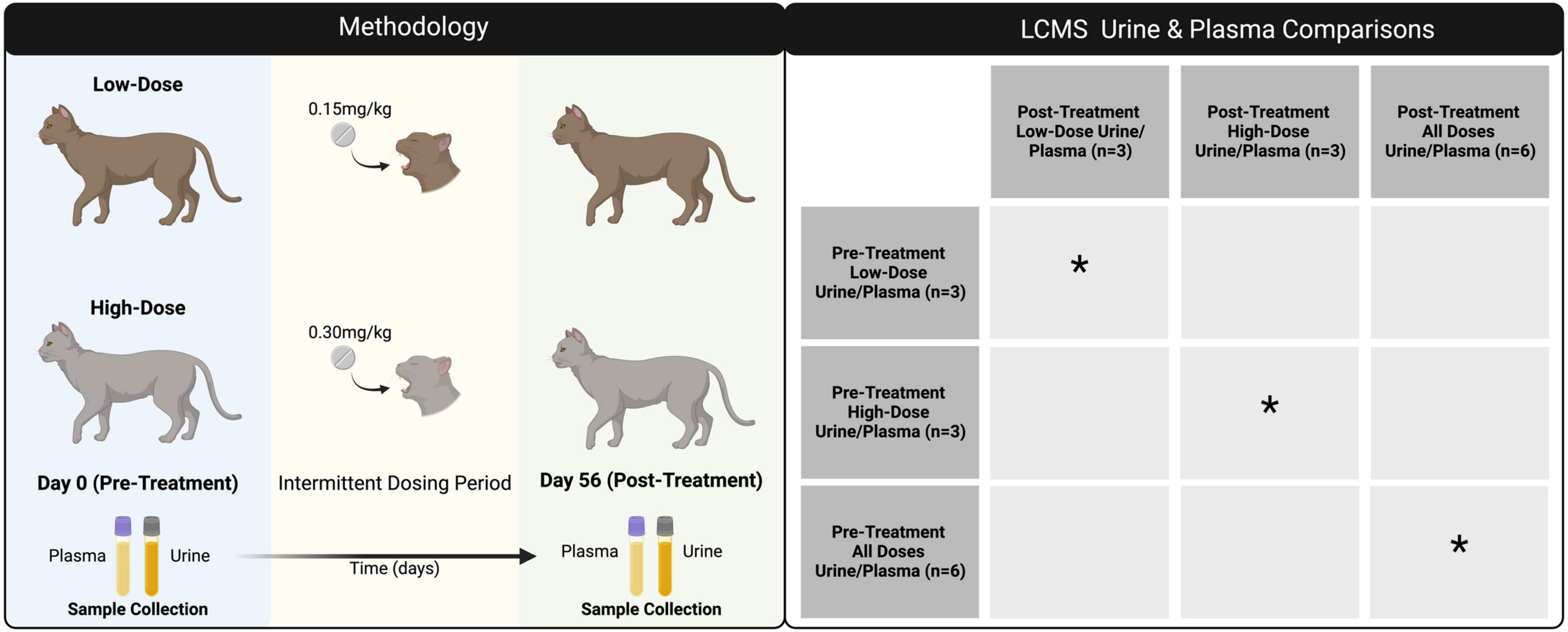

Supplemental Figure S1. Methodology and proteomic comparisons of pre- and post-treatment urine and plasma samples. Illustrative description of LCMS methods and sample collection specification are illustrated. LCMS proteomic comparisons between the pre- and post-treatment of low-, high-, and pooled all dose (low- & high-dose) groups are presented for urine and plasma samples. *Asterisks in the above chart denote comparisons that were performed in this study.

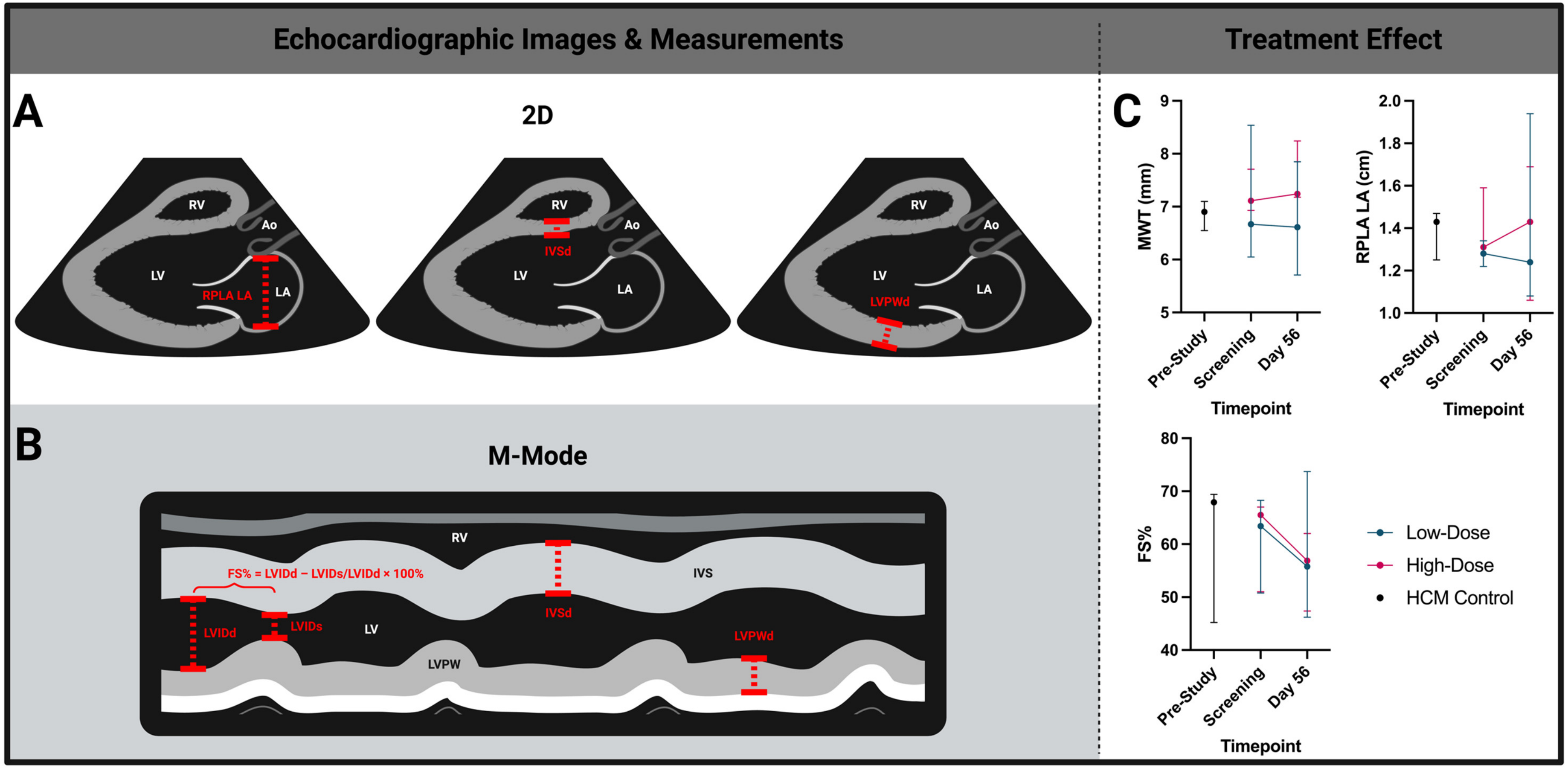

Supplemental Figure S2. Key echocardiographic measurements and treatment-related changes. Representative illustrations of echocardiographic images obtained in 2D right parasternal long-axis (A) and M-mode right parasternal short-axis (B) for the measurement of diastolic maximum wall thickness (MWT) from the IVSd or LVPWd, LA maximal diameter (RPLA LA), and percent LV fractional shortening (FS%) obtained from LV internal diameter measures in diastole and systole (LVIDd and LVIDs, respectively). Screening and Day 56 timepoint echocardiographic measurements are reported for HCM control (single timepoint pre-study) and low- and high-dose treated cats (for Screening and Day 56). In these graphs the median is shown with error bars representing the minimum and maximum values (C); note, the single echocardiographic assessment of untreated HCM controls shows that control cats were well-matched phenotypically to the treated cats.

Abbreviations: LV = left ventricle, RV = right ventricle, LA = left atrium, Ao = aorta, RPLA = right parasternal long-axis, RPLA LA = right parasternal long-axis LA diameter, IVS = interventricular septum, IVSd = diastolic interventricular septum thickness, LVPW = left ventricular posterior wall, LVPWd = diastolic left ventricular posterior wall thickness, FS% = percent fractional shortening, LVIDd = diastolic left ventricular internal diameter, LVIDs = systolic left ventricular internal diameter, MWT = maximum wall thickness, HCM = hypetrophic cardiomyopathy.

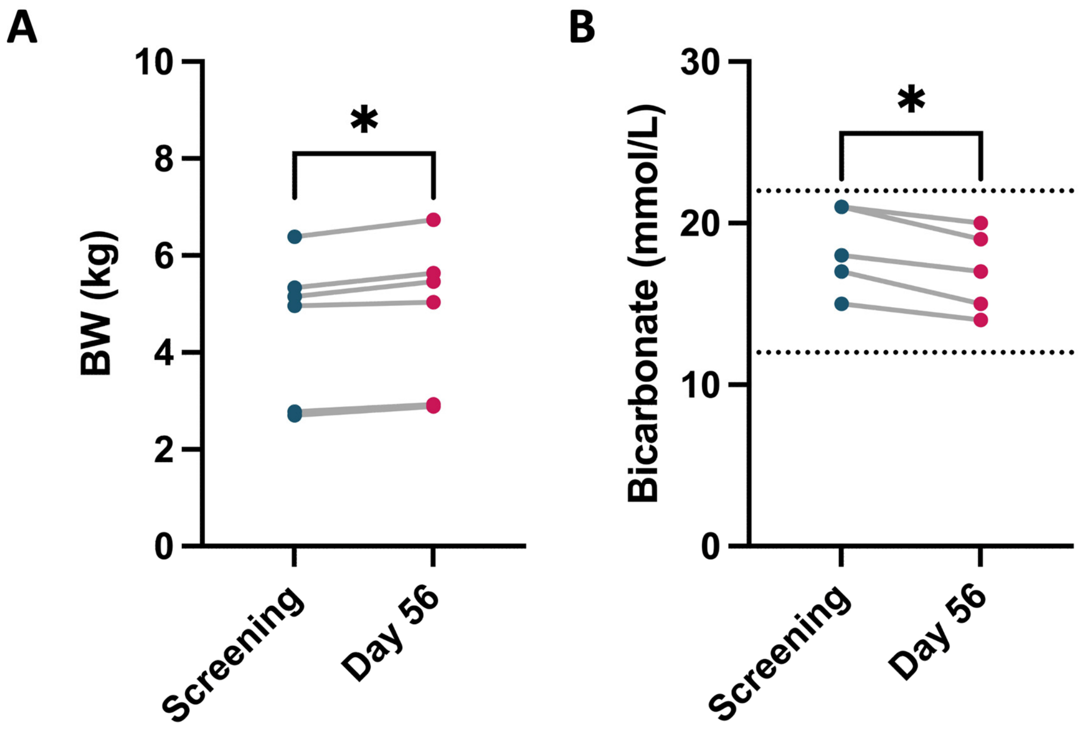

Supplemental Figure S3. Statistically significant physical exam and biochemistry variables. Data points depicting a statistically significant increase in BW (A) and a decrease in blood bicarbonate/total CO_2 levels (B) from pre- (teal) to post-treatment (pink) timepoints for pooled treatment groups (low- + high-dose) are shown ($P<0.03$ and $P<0.03$, respectively). Normal reference range values (12-22mmol/L) for blood bicarbonate/total CO_2 are plotted (dotted lines). *$P<0.05$.

Abbreviations: BW = body weight.

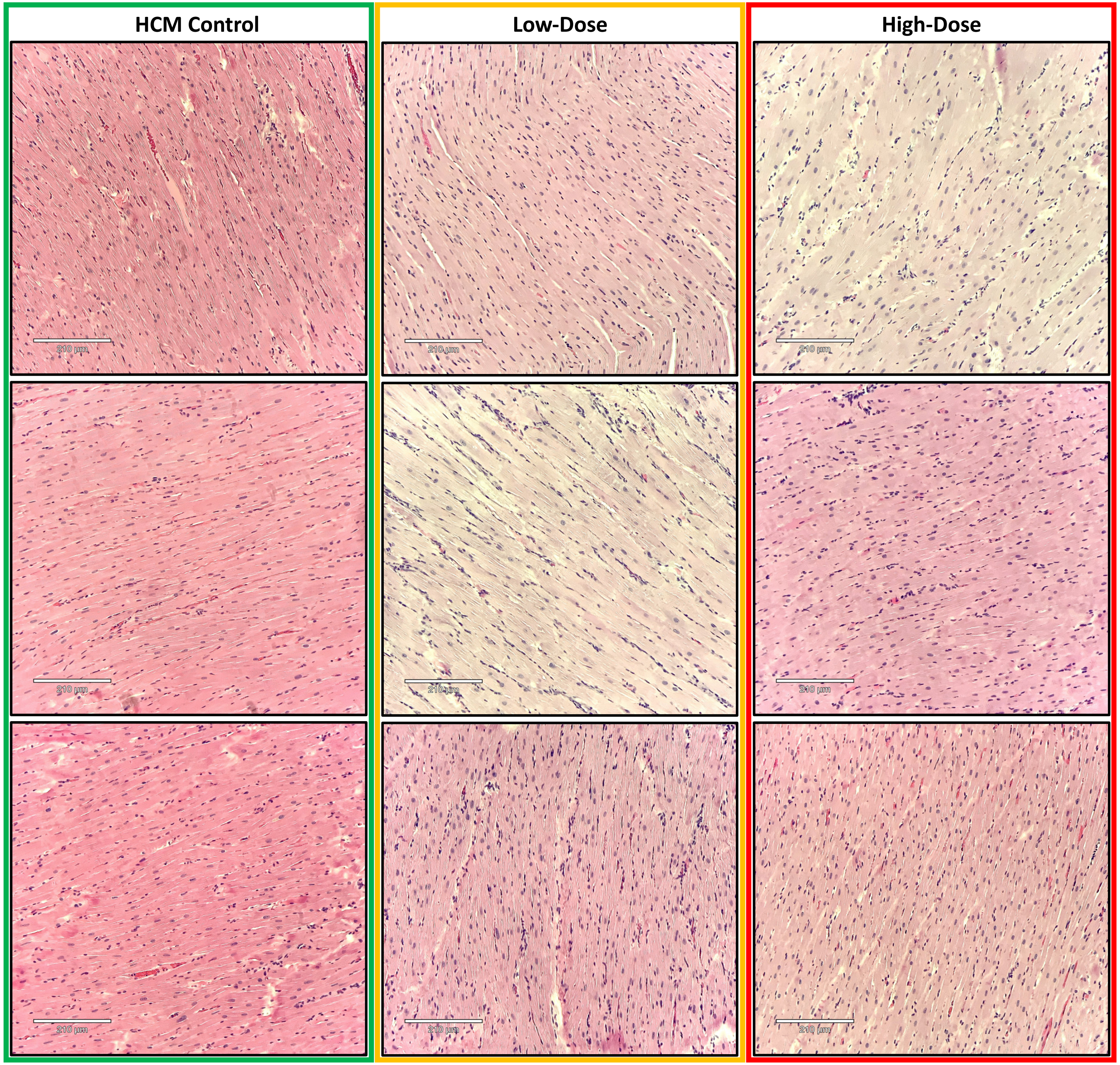
HCM Control
Low-Dose
High-Dose

Supplemental Figure S4. Representative H&E staining of all nine study cats. Histopathologic hematoxylin and eosin (H&E) slide images via light microscopy for all three cats in the HCM control (green box), all three cats in the low-dose (yellow box), and all three cats in the high-dose group (red box) are provided. Images were obtained on the posterior wall of the LV adjacent to papillary muscles. White scale bar=210µm.
Abbreviations: HCM = hypertrophic cardiomyopathy.

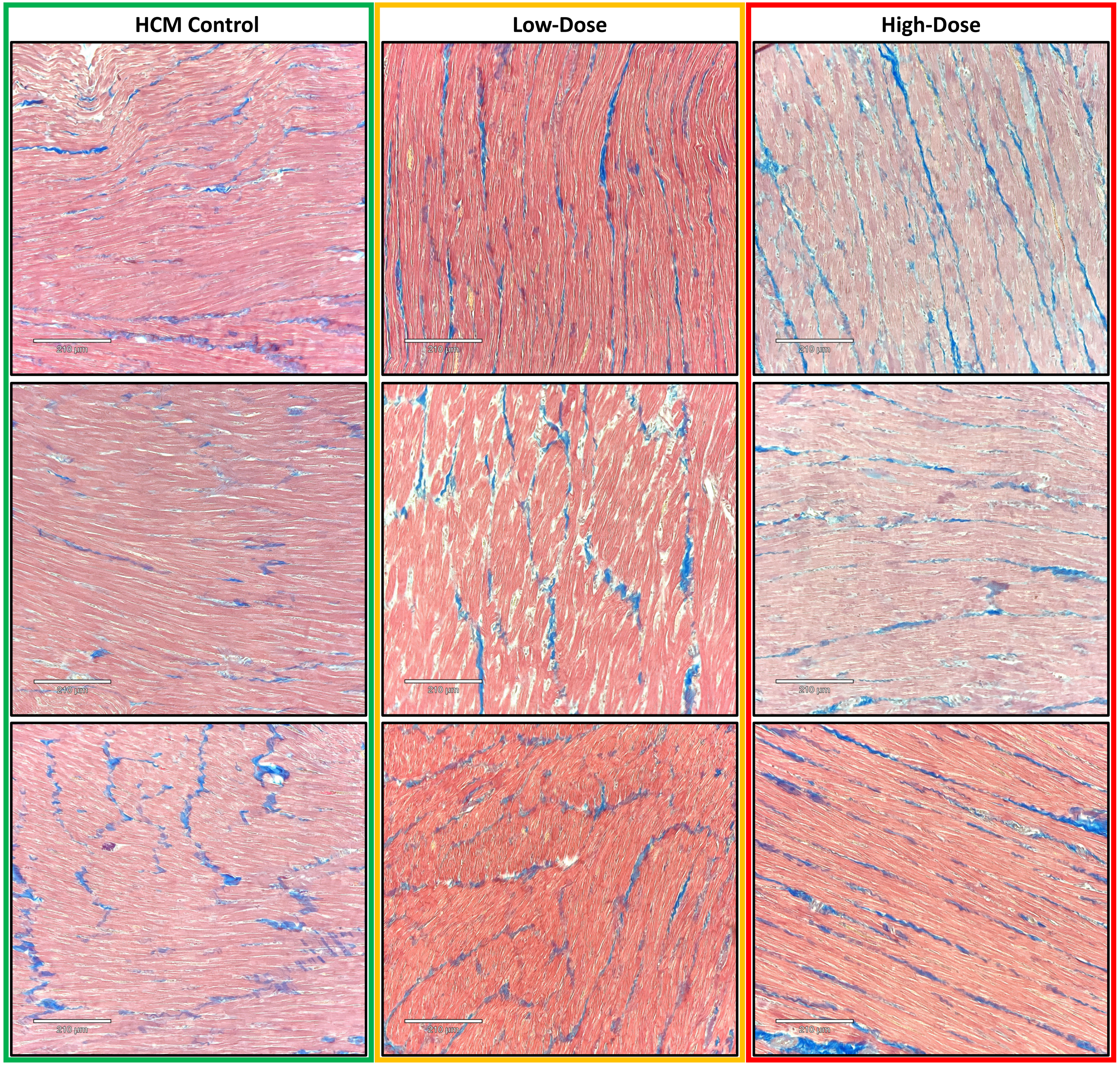

HCM Control
Low-Dose
High-Dose
210 μm

Supplemental Figure S5. Representative Masson’s trichrome staining of all nine study cats. Histopathologic Masson’s trichrome slide images via light microscopy for all three cats in the HCM control (green box), all three cats in the low-dose (yellow box), and all three cats in the high-dose group (red box) are provided. Images were obtained on the posterior wall of the LV adjacent to papillary muscles. White scale bar=210µm.

Abbreviations: HCM = hypertrophic cardiomyopathy.

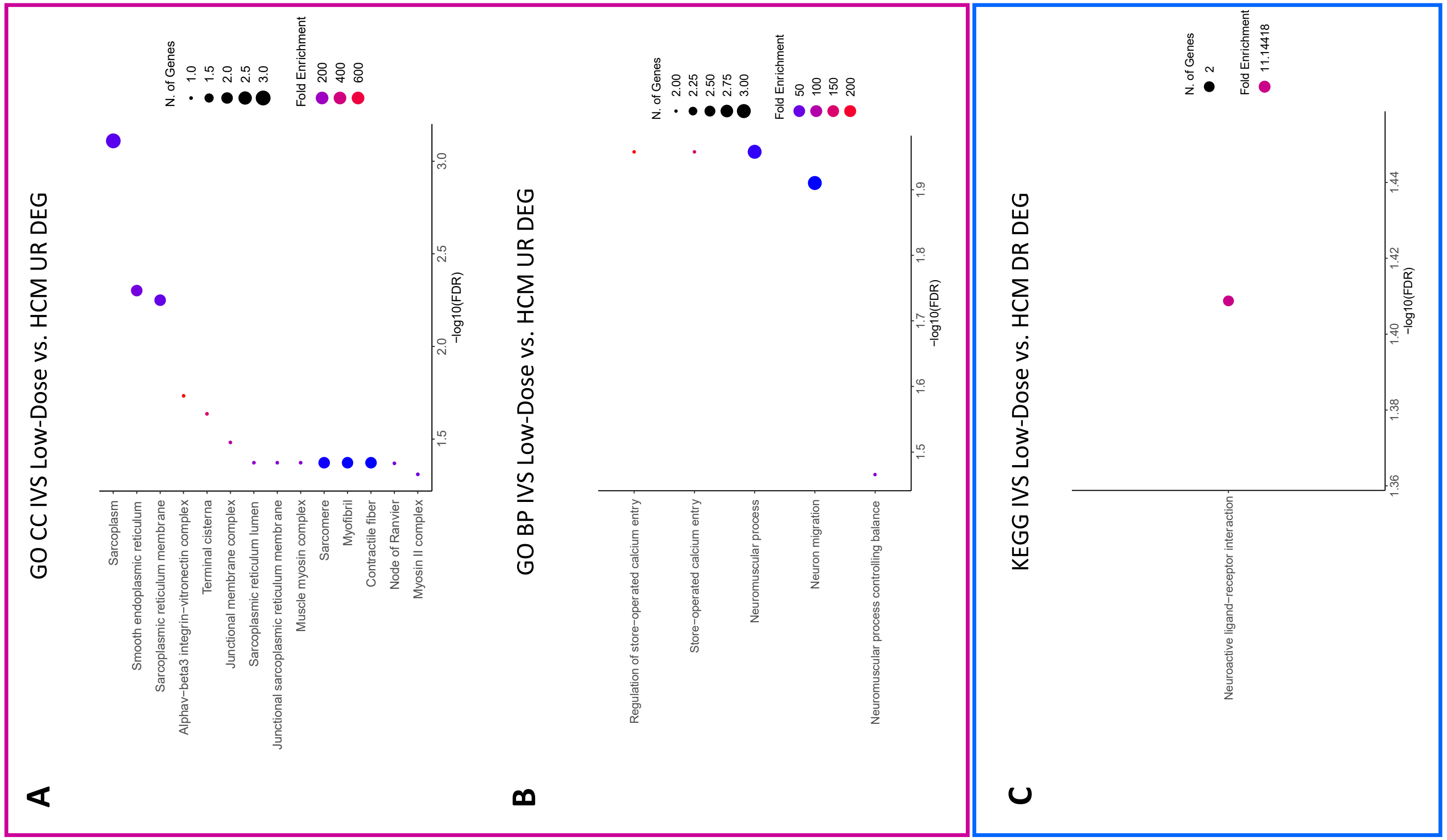

Supplemental Figure S6. Enriched terms for IVS DEGs in the low-dose vs HCM comparison. Enrichment plots for GO CC (A), GO BP (C), and KEGG pathway (C) term analyses are presented for down- (blue box) and upregulated (pink box) DEGs. The y-axis depicts identified enriched terms, whereas the x-axis depicts the terms' significance (-log10[FDR]). Terms are sorted according to statistical significance; the top-most terms on the y-axis constitute the most statistically significant of terms. Size of individual points depict the total number of genes binned to a given enriched term; high or low fold enrichment is represented by red or blue coloring, respectively. Abbreviations: HCM = hypertrophic cardiomyopathy, IVS = interventricular septum, GO = gene ontology, CC = cellular components, BP = biological processes, UR = upregulated, DR = downregulated, DEG = differentially expressed gene(s).

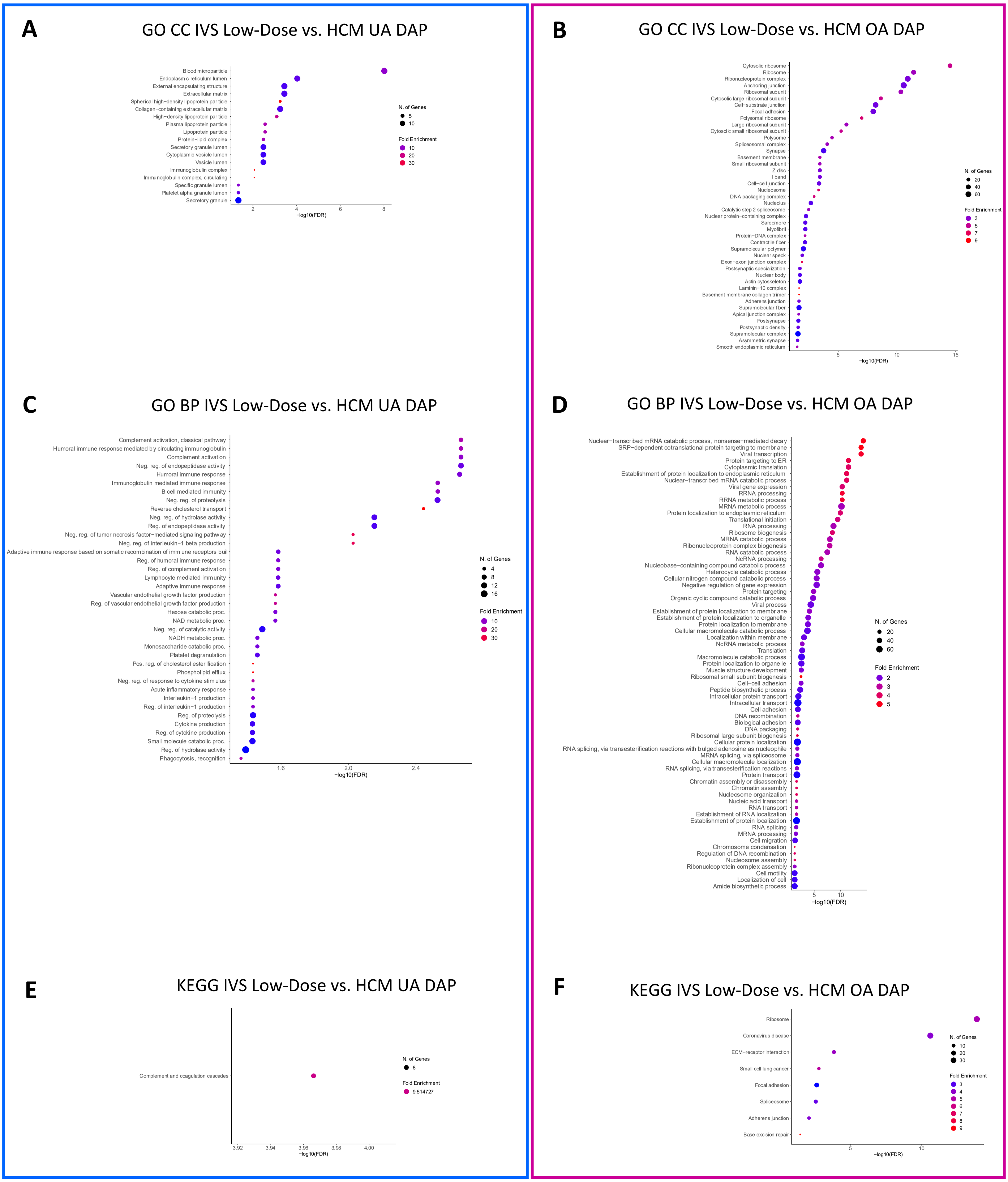
A
GO CC IVS Low-Dose vs. HCM UA DAP
Blood microparticle
Endoplasmic reticulum lumen
External encapsulating structure
Extracellular matrix
Spherical high−density lipoprotein particle
Collagen−containing extracellular matrix
High−density lipoprotein particle
Plasma lipoprotein particle
Lipoprotein particle
Protein−lipid complex
Secretory granule lumen
Cytoplasmic vesicle lumen
Vesicle lumen
Immunoglobulin complex
Immunoglobulin complex, circulating
Specific granule lumen
Platelet alpha granule lumen
Secretory granule
2
4
6
8
−log10(FDR)
N. of Genes
5
10
Fold Enrichment
10
20
30
B
GO CC IVS Low-Dose vs. HCM OA DAP
Cytosolic ribosome
Ribosome
Ribonucleoprotein complex
Anchoring junction
Ribosomal subunit
Cytosolic large ribosomal subunit
Cell−substrate junction
Focal adhesion
Polysomal ribosome
Large ribosomal subunit
Cytosolic small ribosomal subunit
Polysome
Spliceosomal complex
Synapse
Basement membrane
Small ribosomal subunit
Z disc
I band
Cell−cell junction
Nucleosome
DNA packaging complex
Nucleolus
Catalytic step 2 spliceosome
Nuclear protein−containing complex
Sarcomere
Myofibril
Protein−DNA complex
Contractile fiber
Supramolecular polymer
Nuclear speck
Exon−exon junction complex
Postsynaptic specialization
Nuclear body
Actin cytoskeleton
Laminin−10 complex
Basement membrane collagen trimer
Adherens junction
Supramolecular fiber
Apical junction complex
Postsynapse
Postsynaptic density
Supramolecular complex
Asymmetric synapse
Smooth endoplasmic reticulum
5
10
15
−log10(FDR)
N. of Genes
20
40
60
Fold Enrichment
3
5
7
9
C
GO BP IVS Low-Dose vs. HCM UA DAP
Complement activation, classical pathway
Humoral immune response mediated by circulating immunoglobulin
Complement activation
Neg. reg. of endopeptidase activity
Humoral immune response
Immunoglobulin mediated immune response
B cell mediated immunity
Neg. reg. of proteolysis
Reverse cholesterol transport
Neg. reg. of hydrolase activity
Reg. of endopeptidase activity
Neg. reg. of tumor necrosis factor−mediated signaling pathway
Neg. reg. of interleukin−1 beta production
Adaptive immune response based on somatic recombination of imm une receptors buil
Reg. of humoral immune response
Reg. of complement activation
Lymphocyte mediated immunity
Adaptive immune response
Vascular endothelial growth factor production
Reg. of vascular endothelial growth factor production
Hexose catabolic proc.
NAD metabolic proc.
Neg. reg. of catalytic activity
NADH metabolic proc.
Monosaccharide catabolic proc.
Platelet degranulation
Pos. reg. of cholesterol esterification
Phospholipid efflux
Neg. reg. of response to cytokine stimulus
Acute inflammatory response
Interleukin−1 production
Reg. of interleukin−1 production
Reg. of proteolysis
Cytokine production
Reg. of cytokine production
Small molecule catabolic proc.
Reg. of hydrolase activity
Phagocytosis, recognition
1.6
2.0
2.4
−log10(FDR)
N. of Genes
4
8
12
16
Fold Enrichment
10
20
30
D
GO BP IVS Low-Dose vs. HCM OA DAP
Nuclear−transcribed mRNA catabolic process, nonsense−mediated decay
SRP−dependent cotranslational protein targeting to membrane
Viral transcription
Protein targeting to ER
Cytoplasmic translation
Establishment of protein localization to endoplasmic reticulum
Nuclear−transcribed mRNA catabolic process
Viral gene expression
RNA processing
RRNA metabolic process
MRNA metabolic process
Protein localization to endoplasmic reticulum
Translational initiation
RNA processing
Ribosome biogenesis
MRNA catabolic process
Ribonucleoprotein complex biogenesis
RNA catabolic process
NcRNA processing
Nucleobase−containing compound catabolic process
Heterocycle catabolic process
Cellular nitrogen compound catabolic process
Negative regulation of gene expression
Protein targeting
Organic cyclic compound catabolic process
Viral process
Establishment of protein localization to membrane
Establishment of protein localization to organelle
Protein localization to membrane
Cellular macromolecule catabolic process
Localization within membrane
NcRNA metabolic process
Translation
Macromolecule catabolic process
Protein localization to organelle
Muscle structure development
Ribosomal small subunit biogenesis
Cell−cell adhesion
Peptide biosynthetic process
Intracellular protein transport
Intracellular transport
Cell adhesion
DNA recombination
Biological adhesion
DNA packaging
Ribosomal large subunit biogenesis
Cellular protein localization
RNA splicing, via transesterification reactions with bulged adenosine as nucleophile
MRNA splicing, via spliceosome
Cellular macromolecule localization
RNA splicing, via transesterification reactions
Protein transport
Chromatin assembly or disassembly
Chromatin assembly
Nucleosome organization
Nucleic acid transport
RNA transport
Establishment of RNA localization
Establishment of protein localization
RNA splicing
MRNA processing
Cell migration
Chromosome condensation
Regulation of DNA recombination
Nucleosome assembly
Ribonucleoprotein complex assembly
Cell motility
Localization of cell
Amide biosynthetic process
5
10
−log10(FDR)
N. of Genes
20
40
60
Fold Enrichment
2
3
4
5
E
KEGG IVS Low-Dose vs. HCM UA DAP
Complement and coagulation cascades
3.92
3.94
3.96
3.98
4.00
−log10(FDR)
N. of Genes
8
Fold Enrichment
9.514727
F
KEGG IVS Low-Dose vs. HCM OA DAP
Ribosome
Coronavirus disease
ECM−receptor interaction
Small cell lung cancer
Focal adhesion
Spliceosome
Adherens junction
Base excision repair
5
10
−log10(FDR)
N. of Genes
10
20
30
Fold Enrichment
3
4
5
6
7
8
9

Supplemental Figure S7. Enriched terms for IVS DAPs in the low-dose vs HCM comparison. Enrichment plots for GO CC (A & B), GO BP (C & D), and KEGG pathway (E & F) term analyses are presented for under- (blue box) and overabundant (pink box) DAPs. The y-axis depicts identified enriched terms, whereas the x-axis depicts the terms' significance (-log10[FDR]). Terms are sorted according to statistical significance; the top-most terms on the y-axis constitute the most statistically significant of terms. Size of individual points depict the total number of proteins binned to a given enriched term; high or low fold enrichment is represented by red or blue coloring, respectively.

Abbreviations: HCM = hypertrophic cardiomyopathy, IVS = interventricular septum, GO = gene ontology, CC = cellular components, BP = biological processes, UA = underabundant, OA = overabundant, DAP = differentially abundant peptide(s).

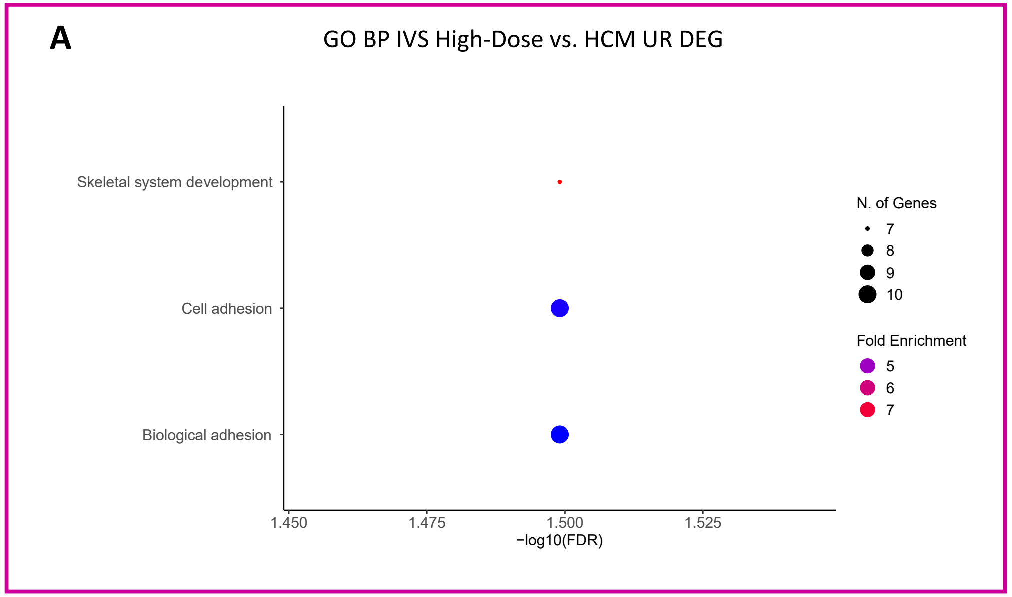

Supplemental Figure S8. Enriched terms for IVS DEGs in the high-dose vs HCM comparison. Enrichment plot for GO CC (A) term analysis are presented for upregulated (pink box) DEGs. The y-axis depicts identified enriched terms, whereas the x-axis depicts the terms' significance (-log10[FDR]). Terms are sorted according to statistical significance; the top-most terms on the y-axis constitute the most statistically significant of terms. Size of individual points depict the total number of genes binned to a given enriched term; high or low fold enrichment is represented by red or blue coloring, respectively.

Abbreviations: HCM = hypertrophic cardiomyopathy, IVS = interventricular septum, GO = gene ontology, BP = biological processes, UR = upregulated, DEG = differentially expressed gene(s).

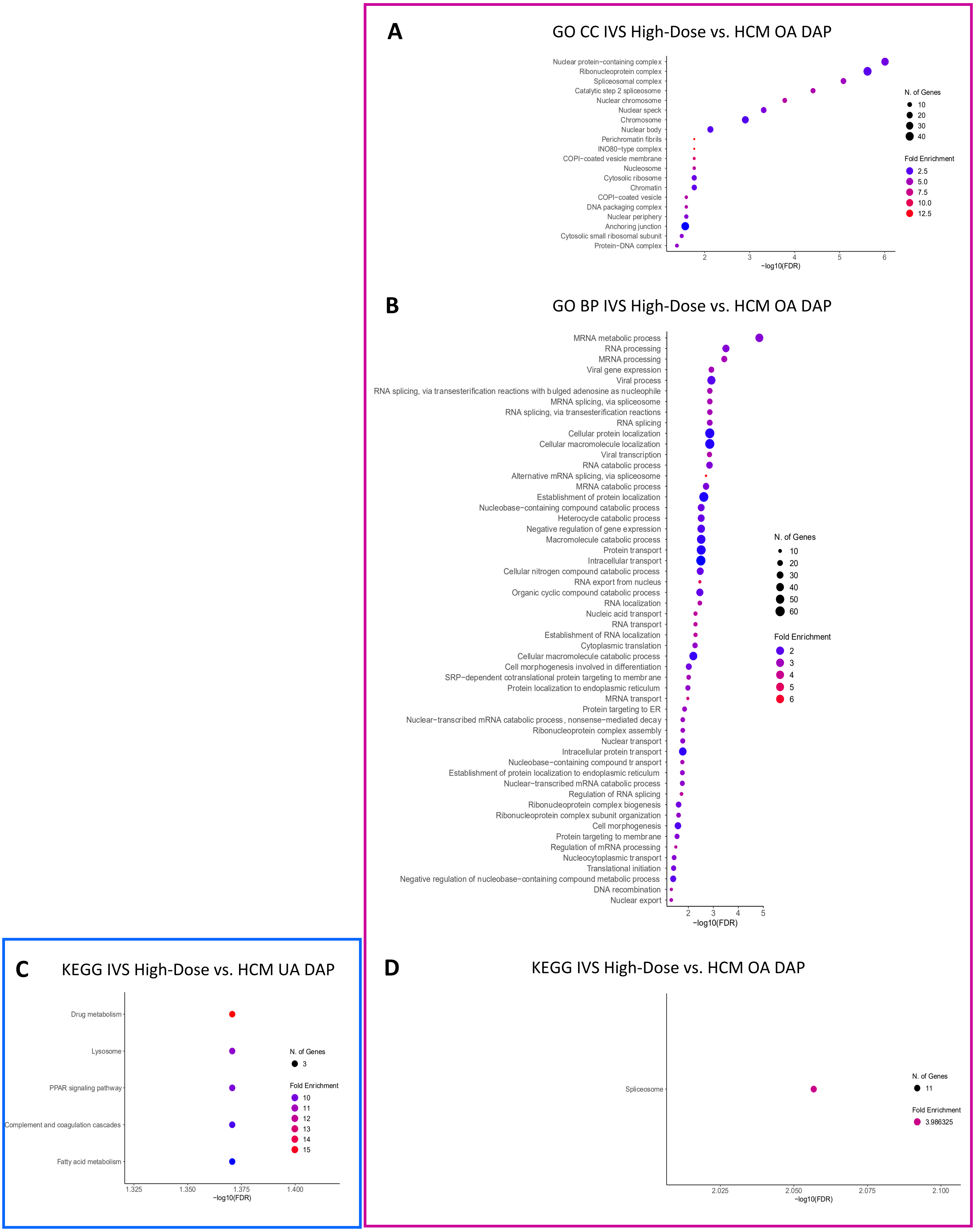
A
GO CC IVS High-Dose vs. HCM OA DAP
Nuclear protein−containing complex
Ribonucleoprotein complex
Spliceosomal complex
Catalytic step 2 spliceosome
Nuclear chromosome
Nuclear speck
Chromosome
Nuclear body
Perichromatin fibrils
INO80−type complex
COPI−coated vesicle membrane
Nucleosome
Cytosolic ribosome
Chromatin
COPI−coated vesicle
DNA packaging complex
Nuclear periphery
Anchoring junction
Cytosolic small ribosomal subunit
Protein−DNA complex
−log10(FDR)
N. of Genes
10
20
30
40
Fold Enrichment
2.5
5.0
7.5
10.0
12.5
B
GO BP IVS High-Dose vs. HCM OA DAP
MRNA metabolic process
RNA processing
MRNA processing
Viral gene expression
Viral process
RNA splicing, via transesterification reactions with bulged adenosine as nucleophile
MRNA splicing, via spliceosome
RNA splicing, via transesterification reactions
RNA splicing
Cellular protein localization
Cellular macromolecule localization
Viral transcription
RNA catabolic process
Alternative mRNA splicing, via spliceosome
MRNA catabolic process
Establishment of protein localization
Nucleobase−containing compound catabolic process
Heterocycle catabolic process
Negative regulation of gene expression
Macromolecule catabolic process
Protein transport
Intracellular transport
Cellular nitrogen compound catabolic process
RNA export from nucleus
Organic cyclic compound catabolic process
RNA localization
Nucleic acid transport
RNA transport
Establishment of RNA localization
Cytoplasmic translation
Cellular macromolecule catabolic process
Cell morphogenesis involved in differentiation
SRP−dependent cotranslational protein targeting to membrane
Protein localization to endoplasmic reticulum
MRNA transport
Protein targeting to ER
Nuclear−transcribed mRNA catabolic process, nonsense−mediated decay
Ribonucleoprotein complex assembly
Nuclear transport
Intracellular protein transport
Nucleobase−containing compound transport
Establishment of protein localization to endoplasmic reticulum
Nuclear−transcribed mRNA catabolic process
Regulation of RNA splicing
Ribonucleoprotein complex biogenesis
Ribonucleoprotein complex subunit organization
Cell morphogenesis
Protein targeting to membrane
Regulation of mRNA processing
Nucleocytoplasmic transport
Translational initiation
Negative regulation of nucleobase−containing compound metabolic process
DNA recombination
Nuclear export
−log10(FDR)
N. of Genes
10
20
30
40
50
60
Fold Enrichment
2
3
4
5
6
C
KEGG IVS High-Dose vs. HCM UA DAP
Drug metabolism
Lysosome
PPAR signaling pathway
Complement and coagulation cascades
Fatty acid metabolism
1.325
1.350
1.375
1.400
−log10(FDR)
N. of Genes
3
Fold Enrichment
10
11
12
13
14
15
D
KEGG IVS High-Dose vs. HCM OA DAP
Spliceosome
2.025
2.050
2.075
2.100
−log10(FDR)
N. of Genes
11
Fold Enrichment
3.986325

Supplemental Figure S9. Enriched terms for IVS DAPs in the high-dose vs HCM comparison. Enrichment plots for GO CC (A), GO BP (C), and KEGG pathway (C & D) term analyses are presented for under- (blue box) and overabundant (pink box) DAPs. The y-axis depicts identified enriched terms, whereas the x-axis depicts the terms' significance (-log10[FDR]). Terms are sorted according to statistical significance; the top-most terms on the y-axis constitute the most statistically significant of terms. Size of individual points depict the total number of proteins binned to a given enriched term; high or low fold enrichment is represented by red or blue coloring, respectively.

Abbreviations: HCM = hypertrophic cardiomyopathy, IVS = interventricular septum, GO = gene ontology, CC = cellular components, BP = biological processes, UA = underabundant, OA = overabundant, DAP = differentially abundant peptide(s).

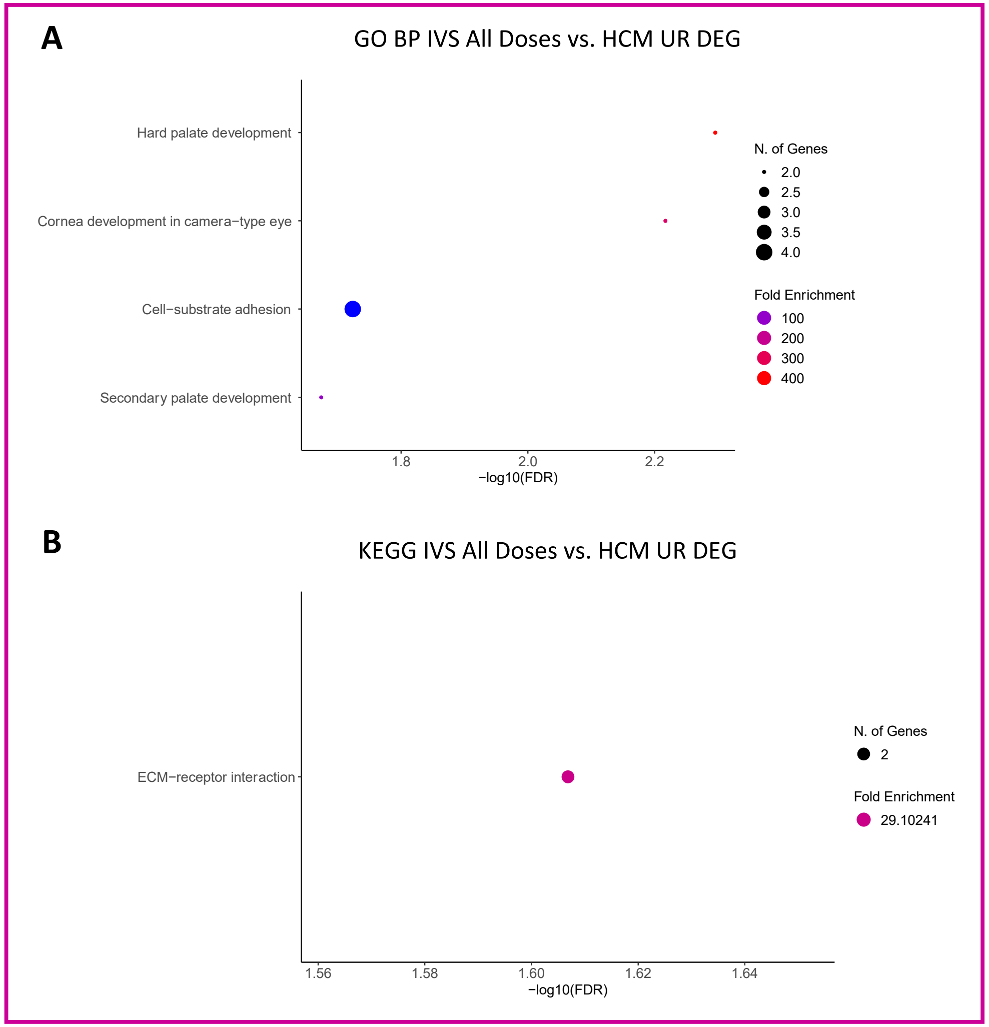

Supplemental Figure S10. Enriched terms for IVS DEGs in the pooled all dose vs HCM comparison. Enrichment plot for GO BP (A) and KEGG pathway (B) term analyses are presented for upregulated (pink box) DEGs. The y-axis depicts identified enriched terms, whereas the x-axis depicts the terms' significance (-log10[FDR]). Terms are sorted according to statistical significance; the top-most terms on the y-axis constitute the most statistically significant of terms. Size of individual points depict the total number of genes binned to a given enriched term; high or low fold enrichment is represented by red or blue coloring, respectively.

Abbreviations: HCM = hypertrophic cardiomyopathy, IVS = interventricular septum, GO = gene ontology, BP = biological processes, UR = upregulated, DEG = differentially expressed gene(s).

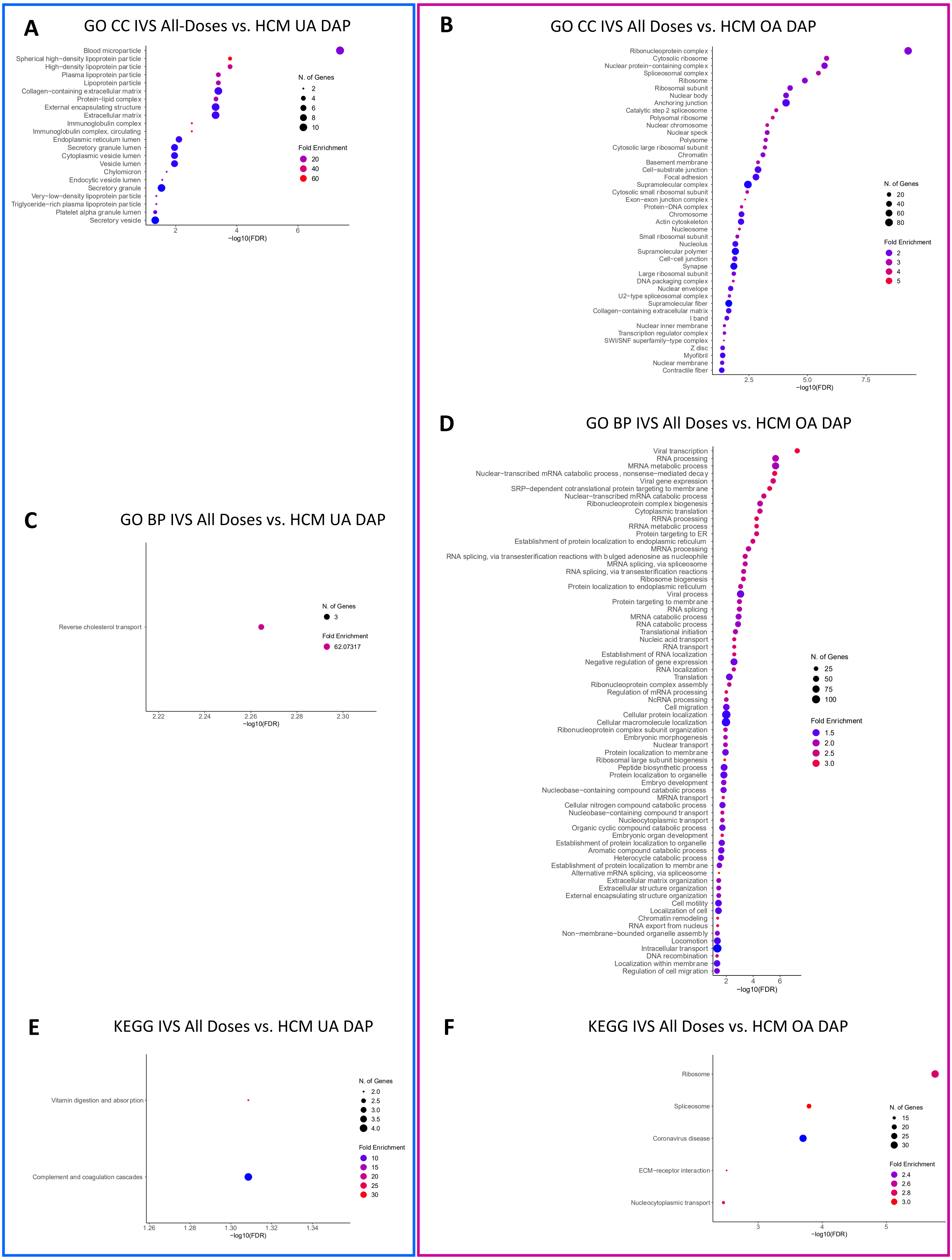
A
GO CC IVS All-Doses vs. HCM UA DAP
Blood microparticle
Spherical high−density lipoprotein particle
High−density lipoprotein particle
Plasma lipoprotein particle
Lipoprotein particle
Collagen−containing extracellular matrix
Protein−lipid complex
External encapsulating structure
Extracellular matrix
Immunoglobulin complex
Immunoglobulin complex, circulating
Endoplasmic reticulum lumen
Secretory granule lumen
Cytoplasmic vesicle lumen
Vesicle lumen
Chylomicron
Endocytic vesicle lumen
Secretory granule
Very−low−density lipoprotein particle
Triglyceride−rich plasma lipoprotein particle
Platelet alpha granule lumen
Secretory vesicle
2
4
6
−log10(FDR)
N. of Genes
2
4
6
8
10
Fold Enrichment
20
40
60
B
GO CC IVS All Doses vs. HCM OA DAP
Ribonucleoprotein complex
Cytosolic ribosome
Nuclear protein−containing complex
Spliceosomal complex
Ribosome
Ribosomal subunit
Nuclear body
Anchoring junction
Catalytic step 2 spliceosome
Polysomal ribosome
Nuclear chromosome
Nuclear speck
Polysome
Cytosolic large ribosomal subunit
Chromatin
Basement membrane
Cell−substrate junction
Focal adhesion
Supramolecular complex
Cytosolic small ribosomal subunit
Exon−exon junction complex
Protein−DNA complex
Chromosome
Actin cytoskeleton
Nucleosome
Small ribosomal subunit
Nucleolus
Supramolecular polymer
Cell−cell junction
Synapse
Large ribosomal subunit
DNA packaging complex
Nuclear envelope
U2−type spliceosomal complex
Supramolecular fiber
Collagen−containing extracellular matrix
I band
Nuclear inner membrane
Transcription regulator complex
SWI/SNF superfamily−type complex
Z disc
Myofibril
Nuclear membrane
Contractile fiber
2.5
5.0
7.5
−log10(FDR)
N. of Genes
20
40
60
80
Fold Enrichment
2
3
4
5
C
GO BP IVS All Doses vs. HCM UA DAP
Reverse cholesterol transport
2.22
2.24
2.26
2.28
2.30
−log10(FDR)
N. of Genes
3
Fold Enrichment
62.07317
D
GO BP IVS All Doses vs. HCM OA DAP
Viral transcription
RNA processing
MRNA metabolic process
Nuclear−transcribed mRNA catabolic process, nonsense−mediated decay
Viral gene expression
SRP−dependent cotranslational protein targeting to membrane
Nuclear−transcribed mRNA catabolic process
Ribonucleoprotein complex biogenesis
Cytoplasmic translation
RRNA processing
RRNA metabolic process
Protein targeting to ER
Establishment of protein localization to endoplasmic reticulum
MRNA processing
RNA splicing, via transesterification reactions with bulged adenosine as nucleophile
MRNA splicing, via spliceosome
RNA splicing, via transesterification reactions
Ribosome biogenesis
Protein localization to endoplasmic reticulum
Viral process
Protein targeting to membrane
RNA splicing
MRNA catabolic process
RNA catabolic process
Translational initiation
Nucleic acid transport
RNA transport
Establishment of RNA localization
Negative regulation of gene expression
RNA localization
Translation
Ribonucleoprotein complex assembly
Regulation of mRNA processing
NcRNA processing
Cell migration
Cellular protein localization
Cellular macromolecule localization
Ribonucleoprotein complex subunit organization
Embryonic morphogenesis
Nuclear transport
Protein localization to membrane
Ribosomal large subunit biogenesis
Peptide biosynthetic process
Protein localization to organelle
Embryo development
Nucleobase−containing compound catabolic process
MRNA transport
Cellular nitrogen compound catabolic process
Nucleobase−containing compound transport
Nucleocytoplasmic transport
Organic cyclic compound catabolic process
Embryonic organ development
Establishment of protein localization to organelle
Aromatic compound catabolic process
Heterocycle catabolic process
Establishment of protein localization to membrane
Alternative mRNA splicing, via spliceosome
Extracellular matrix organization
Extracellular structure organization
External encapsulating structure organization
Cell motility
Localization of cell
Chromatin remodeling
RNA export from nucleus
Non−membrane−bounded organelle assembly
Locomotion
Intracellular transport
DNA recombination
Localization within membrane
Regulation of cell migration
2
4
6
−log10(FDR)
N. of Genes
25
50
75
100
Fold Enrichment
1.5
2.0
2.5
3.0
E
KEGG IVS All Doses vs. HCM UA DAP
Vitamin digestion and absorption
Complement and coagulation cascades
1.26
1.28
1.30
1.32
1.34
−log10(FDR)
N. of Genes
2.0
2.5
3.0
3.5
4.0
Fold Enrichment
10
15
20
25
30
F
KEGG IVS All Doses vs. HCM OA DAP
Ribosome
Spliceosome
Coronavirus disease
ECM−receptor interaction
Nucleocytoplasmic transport
3
4
5
−log10(FDR)
N. of Genes
15
20
25
30
Fold Enrichment
2.4
2.6
2.8
3.0

Supplemental Figure S11. Enriched terms for IVS DAPs in the pooled all doses vs HCM comparison. Enrichment plots for GO CC (A & B), GO BP (C & D), and KEGG pathway (E & F) term analyses are presented for under- (blue box) and overabundant (pink box) DAPs. The y-axis depicts identified enriched terms, whereas the x-axis depicts the terms' significance (-log10[FDR]). Terms are sorted according to statistical significance; the top-most terms on the y-axis constitute the most statistically significant of terms. Size of individual points depict the total number of proteins binned to a given enriched term; high or low fold enrichment is represented by red or blue coloring, respectively.

Abbreviations: HCM = hypertrophic cardiomyopathy, IVS = interventricular septum, GO = gene ontology, CC = cellular components, BP = biological processes, UA = underabundant, OA = overabundant, DAP = differentially abundant peptide(s).

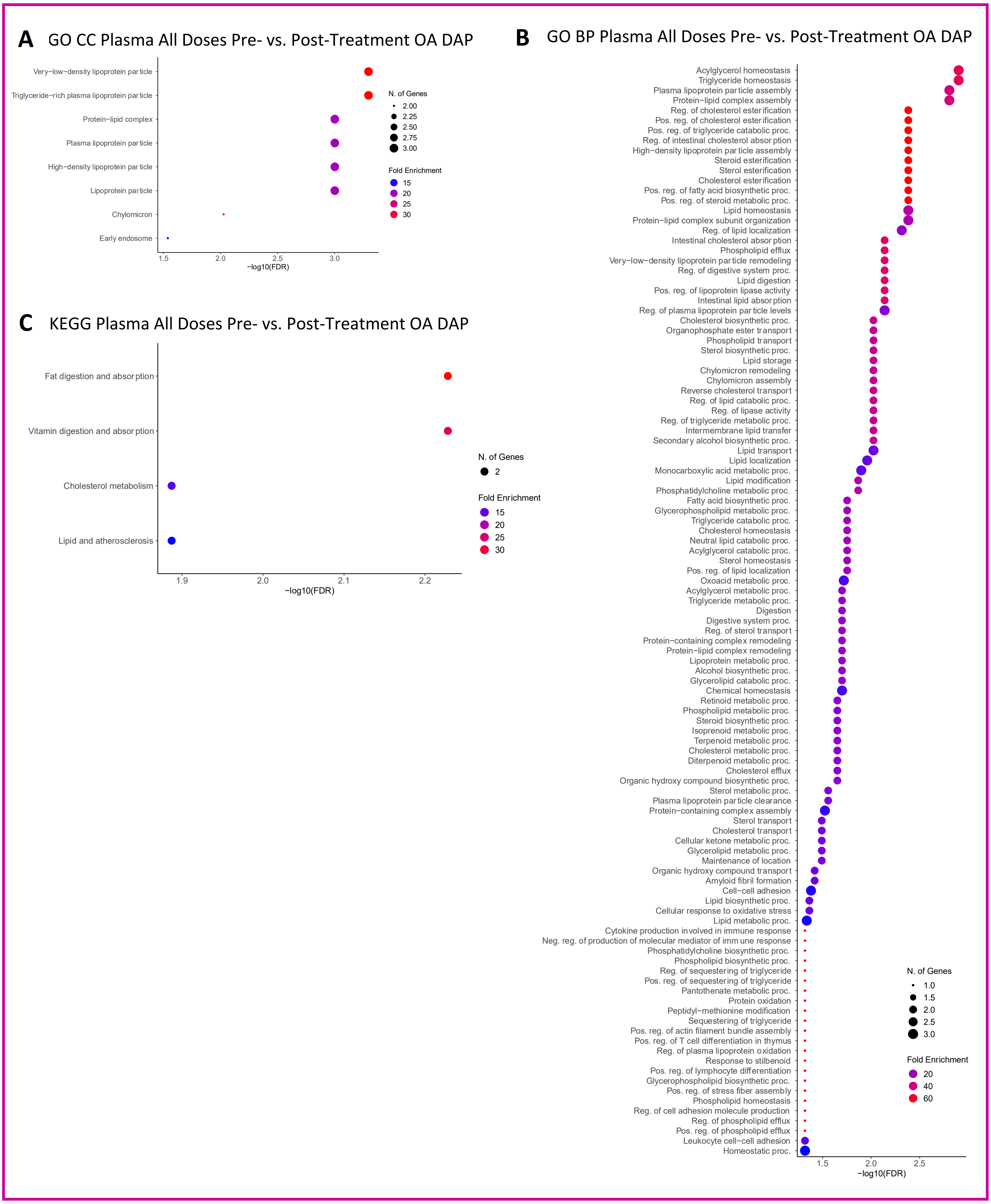
A GO CC Plasma All Doses Pre- vs. Post-Treatment OA DAP
Very−low−density lipoprotein particle
Triglyceride−rich plasma lipoprotein particle
Protein−lipid complex
Plasma lipoprotein particle
High−density lipoprotein particle
Lipoprotein particle
Chylomicron
Early endosome
1.5
2.0
2.5
3.0
−log10(FDR)
N. of Genes
2.00
2.25
2.50
2.75
3.00
Fold Enrichment
15
20
25
30
B GO BP Plasma All Doses Pre- vs. Post-Treatment OA DAP
Acylglycerol homeostasis
Triglyceride homeostasis
Plasma lipoprotein particle assembly
Protein−lipid complex assembly
Reg. of cholesterol esterification
Pos. reg. of cholesterol esterification
Pos. reg. of triglyceride catabolic proc.
Reg. of intestinal cholesterol absorption
High−density lipoprotein particle assembly
Steroid esterification
Sterol esterification
Cholesterol esterification
Pos. reg. of fatty acid biosynthetic proc.
Pos. reg. of steroid metabolic proc.
Lipid homeostasis
Protein−lipid complex subunit organization
Reg. of lipid localization
Intestinal cholesterol absorption
Phospholipid efflux
Very−low−density lipoprotein particle remodeling
Reg. of digestive system proc.
Lipid digestion
Pos. reg. of lipoprotein lipase activity
Intestinal lipid absorption
Reg. of plasma lipoprotein particle levels
Cholesterol biosynthetic proc.
Organophosphate ester transport
Phospholipid transport
Sterol biosynthetic proc.
Lipid storage
Chylomicron remodeling
Chylomicron assembly
Reverse cholesterol transport
Reg. of lipid catabolic proc.
Reg. of lipase activity
Reg. of triglyceride metabolic proc.
Intermembrane lipid transfer
Secondary alcohol biosynthetic proc.
Lipid transport
Lipid localization
Monocarboxylic acid metabolic proc.
Lipid modification
Phosphatidylcholine metabolic proc.
Fatty acid biosynthetic proc.
Glycerophospholipid metabolic proc.
Triglyceride catabolic proc.
Cholesterol homeostasis
Neutral lipid catabolic proc.
Acylglycerol catabolic proc.
Sterol homeostasis
Pos. reg. of lipid localization
Oxoacid metabolic proc.
Acylglycerol metabolic proc.
Triglyceride metabolic proc.
Digestion
Digestive system proc.
Reg. of sterol transport
Protein−containing complex remodeling
Protein−lipid complex remodeling
Lipoprotein metabolic proc.
Alcohol biosynthetic proc.
Glycerolipid catabolic proc.
Chemical homeostasis
Retinoid metabolic proc.
Phospholipid metabolic proc.
Steroid biosynthetic proc.
Isoprenoid metabolic proc.
Terpenoid metabolic proc.
Cholesterol metabolic proc.
Diterpenoid metabolic proc.
Cholesterol efflux
Organic hydroxy compound biosynthetic proc.
Sterol metabolic proc.
Plasma lipoprotein particle clearance
Protein−containing complex assembly
Sterol transport
Cholesterol transport
Cellular ketone metabolic proc.
Glycerolipid metabolic proc.
Maintenance of location
Organic hydroxy compound transport
Amyloid fibril formation
Cell−cell adhesion
Lipid biosynthetic proc.
Cellular response to oxidative stress
Lipid metabolic proc.
Cytokine production involved in immune response
Neg. reg. of production of molecular mediator of immune response
Phosphatidylcholine biosynthetic proc.
Phospholipid biosynthetic proc.
Reg. of sequestering of triglyceride
Pos. reg. of sequestering of triglyceride
Pantothenate metabolic proc.
Protein oxidation
Peptidyl−methionine modification
Sequestering of triglyceride
Pos. reg. of actin filament bundle assembly
Pos. reg. of T cell differentiation in thymus
Reg. of plasma lipoprotein oxidation
Response to stilbenoid
Pos. reg. of lymphocyte differentiation
Glycerophospholipid biosynthetic proc.
Pos. reg. of stress fiber assembly
Phospholipid homeostasis
Reg. of cell adhesion molecule production
Reg. of phospholipid efflux
Pos. reg. of phospholipid efflux
Leukocyte cell−cell adhesion
Homeostatic proc.
1.5
2.0
2.5
−log10(FDR)
N. of Genes
1.0
1.5
2.0
2.5
3.0
Fold Enrichment
20
40
60
C KEGG Plasma All Doses Pre- vs. Post-Treatment OA DAP
Fat digestion and absorption
Vitamin digestion and absorption
Cholesterol metabolism
Lipid and atherosclerosis
1.9
2.0
2.1
2.2
−log10(FDR)
N. of Genes
2
Fold Enrichment
15
20
25
30

Supplemental Figure S12. Enriched terms for plasma pre- and post-treatment DAPs in the pooled all doses group. Enrichment plots for GO CC (A), GO BP (B), and KEGG pathway (C) term analyses are presented for overabundant (pink box) DAPs. The y-axis depicts identified enriched terms, whereas the x-axis depicts the terms' significance (-log10[FDR]). Terms are sorted according to statistical significance; the top-most terms on the y-axis constitute the most statistically significant of terms. Size of individual points depict the total number of proteins binned to a given enriched term; high or low fold enrichment is represented by red or blue coloring, respectively. Abbreviations: GO = gene ontology, CC = cellular components, BP = biological processes, OA = overabundant, DAP = differentially abundant peptide(s).

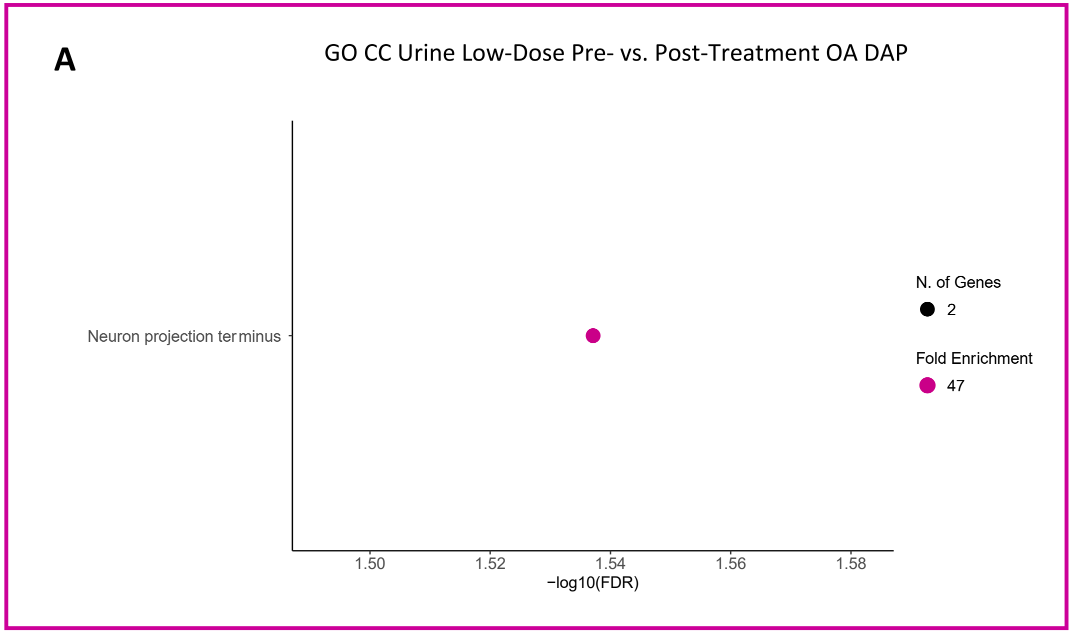

Supplemental Figure S13. Enriched term for urine pre- and post-treatment DAPs in the low-dose group. Enrichment plot for the GO CC (A) term analysis is presented for overabundant (pink box) DAPs. The y-axis depicts identified enriched terms, whereas the x-axis depicts the terms' significance (-log10[FDR]). Size of individual points depict the total number of proteins binned to the given enriched term; high or low fold enrichment is represented by red or blue coloring, respectively.
Abbreviations: GO = gene ontology, CC = cellular components, OA = overabundant, DAP = differentially abundant peptide(s).

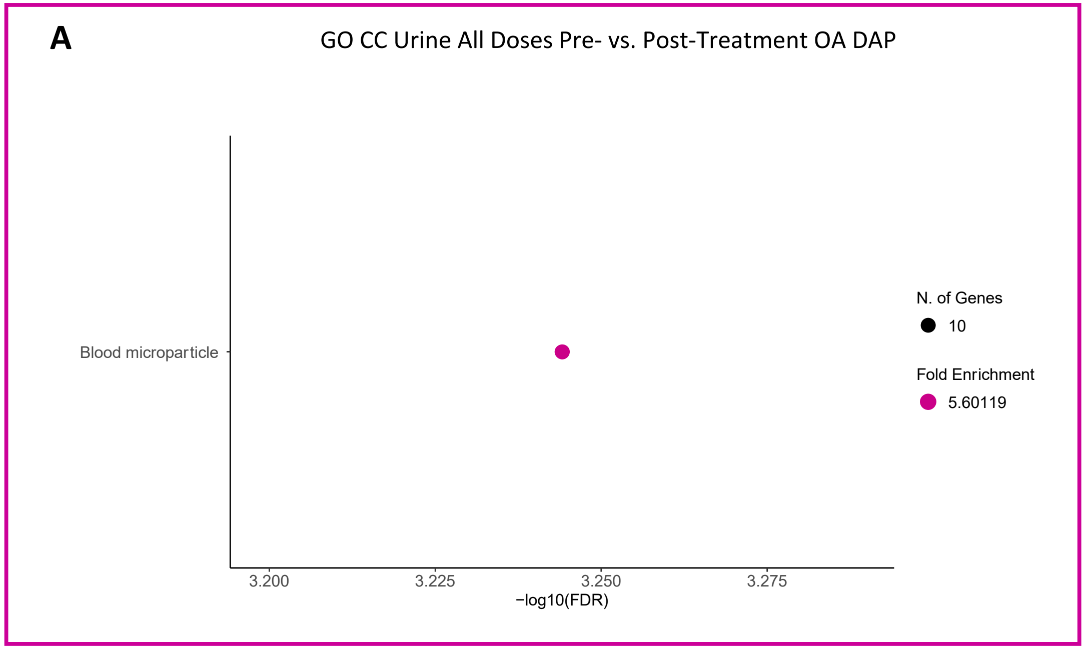

Supplemental Figure S14. Enriched term for urine pre- and post-treatment DAPs in the pooled all doses group. Enrichment plot for the GO CC (A) term analysis is presented for overabundant (pink box) DAPs. The y-axis depicts identified enriched terms, whereas the x-axis depicts the terms' significance (-log10[FDR]). Terms are sorted according to statistical significance; the top-most terms on the y-axis constitute the most statistically significant of terms. Size of individual points depict the total number of proteins binned to the given enriched term; high or low fold enrichment is represented by red or blue coloring, respectively.
Abbreviations: GO = gene ontology, CC = cellular components, OA = overabundant, DAP = differentially abundant peptide(s).
